# G protein-coupled receptors in the hypothalamic paraventricular and supraoptic nuclei – serpentine gateways to neuroendocrine homeostasis

**DOI:** 10.1016/j.yfrne.2011.07.002

**Published:** 2012-01

**Authors:** Georgina G.J. Hazell, Charles C. Hindmarch, George R. Pope, James A. Roper, Stafford L. Lightman, David Murphy, Anne-Marie O’Carroll, Stephen J. Lolait

**Affiliations:** Henry Wellcome Laboratories for Integrative Neuroscience and Endocrinology, Dorothy Hodgkin Building, School of Clinical Sciences, University of Bristol, Whitson Street, Bristol BS1 3NY, UK

**Keywords:** G protein-coupled receptor, Paraventricular nucleus, Supraoptic nucleus, Vasopressin, Oxytocin, Corticotropin-releasing factor, Hypothalamo-neurohypophysial system (HNS), Hypothalamus–pituitary–adrenal (HPA) axis

## Abstract

G protein-coupled receptors (GPCRs) are the largest family of transmembrane receptors in the mammalian genome. They are activated by a multitude of different ligands that elicit rapid intracellular responses to regulate cell function. Unsurprisingly, a large proportion of therapeutic agents target these receptors. The paraventricular nucleus (PVN) and supraoptic nucleus (SON) of the hypothalamus are important mediators in homeostatic control. Many modulators of PVN/SON activity, including neurotransmitters and hormones act via GPCRs – in fact over 100 non-chemosensory GPCRs have been detected in either the PVN or SON. This review provides a comprehensive summary of the expression of GPCRs within the PVN/SON, including data from recent transcriptomic studies that potentially expand the repertoire of GPCRs that may have functional roles in these hypothalamic nuclei. We also present some aspects of the regulation and known roles of GPCRs in PVN/SON, which are likely complemented by the activity of ‘orphan’ GPCRs.

## Graphical abstract

**Graphical abstract:**

**Figure:**
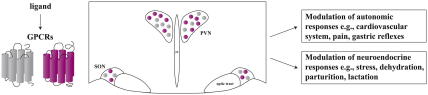

**Highlights:**

► The paraventricular and supraoptic nuclei of the hypothalamus are regulators of homeostasis. ► Over one hundred G protein-coupled receptors are expressed in each of these nuclei. ► The receptors have many functions including modulating neuropeptide synthesis and release. ► 20–30% of the receptors are ‘orphans’ whose endogenous ligand and function is unknown.

## 1 Introduction

The hypothalamo-neurohypophysial system (HNS) responds to dehydration by increasing vasopressin (VP) and oxytocin (OT) gene transcription and translation, and releasing large amounts of VP and OT into the systemic circulation. Similarly, acute and chronic stress, pregnancy, and lactation are all associated with phenotypic changes in the paraventricular (PVN) and/or supraoptic (SON) nuclei of the hypothalamus that include elevations in VP, OT and/or corticotropin-releasing factor (CRF) gene expression [2,42]. Alterations in the pattern and/or level of modulating inputs (e.g., receptor-driven signals) that impinge on the PVN and SON have important functional implications in the control of the HNS and the hypothalamic–pituitary–adrenal (HPA) axis response to stress. The activity of such inputs may drive changes in the PVN/SON VP and OT signature associated with a number of neuroendocrinological disturbances [314], and contribute to the dysregulation of the HPA axis implicated in many conditions including the classical psychosomatic disorders, cardiovascular disease, diabetes and affective disorders such as depression. Receptor function in the PVN/SON may also be altered in immunologically-related disturbances such as immunosuppression and autoimmune, allergic and inflammatory states [48,262].

By virtue of their expression in the PVN and SON, many receptors are key targets for regulating hypothalamic–HNS and -HPA axis activity. There are four major classes of receptors in the central nervous system (CNS) – (1) the ionotropic receptors such as the excitatory glutamate (e.g., N-methyl-d-aspartate (NMDA)) or inhibitory GABA_A_ receptors that create a membrane pore to allow the flow of ions, and have a very rapid response time; (2) receptor tyrosine kinases such as tyrosine kinase receptor type B (TrkB) and the epidermal growth factor receptor (EGFR), which upon stimulation activate intracellular signaling networks like the mitogen-activated protein kinase/extracellular signal-regulated kinase (MAPK/ERK) pathway; (3) nuclear receptors including glucocorticoid, sex steroid and thyroid hormone receptors that regulate transcriptional activation; and (4) G protein-coupled receptors (GPCRs), or seven transmembrane (TM) receptors, occasionally termed heptahelical or ‘serpentine’ receptors. GPCRs constitute the largest superfamily of transmembrane signaling molecules, estimated to comprise about 1900 members (not including pseudogenes) in the rat and mouse genomes, and at least 800 members in the human genome [99]. The proportion of one-to-one GPCR orthologues is approximately 60% between rats and humans, primarily due to divergence in chemosensory receptors that are activated by sensory signals of external origin such as odors, pheromones or tastes. Olfactory receptors make up about 60% of all GPCRs in the rat and mouse genome and 50% in the human genome [99]. Most other GPCRs are activated by a diverse array of endogenous, extracellular (and perhaps intracellular) signals that include photons, biogenic amines, neuropeptides, amino acids, ions, hormones, chemokines, lipid-derived mediators and proteases. Upon ligand binding, GPCRs primarily transduce these signals via the heterotrimeric G proteins into rapid intracellular responses that regulate cell function (e.g., increases in protein kinase C (PKC) and/or protein kinase A (PKA) activity, intracellular Ca^2+^ and cyclic AMP (cAMP)). It is estimated that 80% of all known hormones and neurotransmitters activate cellular signal transduction mechanisms via GPCRs [21], and a substantial portion (estimates vary between 30% and 60%) of current pharmaceutical agents directly or indirectly act on these receptors [130,205] – angiotensin II and aminergic (adrenoceptor, dopamine, 5-hydroxytryptamine (5-HT)) receptor subtypes feature as prominent drug targets [137]. There are still 120–130 non-chemosensory ‘orphan’ GPCRs for which the corresponding ligands have not yet been identified [99,111].

In this review we will highlight efforts to examine GPCR expression and regulation in the rat PVN and SON by receptor autoradiography (ARG), immunohistochemistry (IHC) and *in situ* hybridization histochemistry (ISHH) methods in conjunction with more recent transcriptomic approaches (e.g., DNA microarrays) to present an overall estimate of the GPCR repertoire expressed in the PVN and SON. We have collated data on all of the known non-chemosensory GPCRs documented in the on-line International Union of Basic and Clinical Pharmacology Committee on Receptor Nomenclature and Drug Classification database (NC-IUPHAR; http://www.iuphar-db.org/) [111] including orphan GPCRs. These data will be discussed in the context of the regulation and function (and possible redundancy) of GPCRs in the PVN and SON as revealed by current pharmacological/physiological approaches in rats, the species in which the vast majority of studies on the HNS and HPA axis have been conducted. With respect to GPCR localization in the PVN and SON we have endeavored to cite as many of the original reference sources as possible – we apologize in advance if we have inadvertently omitted some citations. The architecture of the PVN/SON, GPCR structure/function studies, and the function of many neurotransmitters/neurohormones in the PVN and/or SON have been extensively reviewed and we shall refer to these papers throughout. In this review we shall use the GPCR subfamily nomenclature (e.g., 5-HT_1A_; rather than rat gene name (e.g., Htr1a)) as per NC-IUPHAR recommendations [111].

## 2 Anatomy and function of the rat PVN and SON

The hypothalamus is essential for maintaining homeostatic equilibrium, integrating signals from other brain regions to regulate an assortment of functions including temperature regulation, appetite and fertility. Within the hypothalamus the PVN and SON are two of the most exhaustively studied nuclei and are fundamental in the control of fluid homeostasis, lactation, cardiovascular regulation, feeding behavior, nociception, behavior and the response to stress. The PVN is located either side of the third ventricle, and can be subdivided into five parvocellular (pPVN) (periventricular, anterior, medial, dorsal and lateral parts) and three magnocellular (mPVN) (anterior, medial and posterior parts) divisions [295,316]. The main neuronal populations in the mPVN and pPVN subdivisions are intermingled with interneurons and supporting cells such as glia. The SON straddles the lateral border of the optic chiasm and contains a ‘homogeneous’ population of magnocellular neurons [255]. The large magnocellular neurons in the PVN and SON secrete mainly VP and OT as part of the HNS whereas the smaller parvocellular neurons elaborate primarily CRF, VP and OT as part of the HPA axis and/or regulate autonomic activity. Elegant, detailed studies on the mapping of the spatial organization of major neuroendocrine and non-neuroendocrine neurons in the rat PVN have revealed that although neuroendocrine neuron clusters display a unique distribution pattern, there is extensive overlap between different phenotypes [295]. For instance, there is some intermixing of magnocellular and parvocellular neurons particularly at the mPVN/pPVN border [295], and isolated magnocellular cells in the pPVN have been noted [75]. Moreover, there is evidence that the HNS and HPA axis may functionally overlap but the extent of this interaction is not fully understood [75].

Magnocellular neurons of both the PVN and SON project via the internal zone of the median eminence to the posterior pituitary, and upon appropriate stimulation secrete VP and/or OT into the peripheral blood. Magnocellular VP is released mainly in response to dehydration, hypovolemia and hypotension, while magnocellular OT is primarily involved in the milk ejection reflex during lactation, and uterine contraction at the later stages of parturition [98,255,265,311]. Parvocellular neurons project from the periventricular, anterior, and medial (dorsal portion) parts of the PVN to the external zone of the median eminence, and release their peptides into the hypophysial portal system, a series of blood vessels that bathe the anterior lobe of the pituitary. In response to stressful stimuli, CRF and VP from the dorsomedial pPVN stimulate the release of adrenocorticotropin releasing hormone (ACTH) from the corticotrope cells of the anterior pituitary, which in turn induces the secretion of cortisol (corticosterone (CORT) in rodents) from the adrenal glands. CORT exerts a negative feedback action on the pituitary, PVN and other brain regions such as the hippocampus to restrict the dramatic initial release of ACTH and CORT [7]. OT can either potentiate or inhibit ACTH and/or CORT responses by binding to the pituitary VP V_1B_ receptor or by an action on central OT receptors, respectively [273,354]. In addition, OT stimulates the release of luteinising hormone from gonadotropes, and prolactin from lactotropes in the anterior pituitary [98]. Other parvocellular neuroendocrine cells include those that express growth hormone-releasing hormone (GHRH), somatostatin, dopamine and thyrotropin-releasing hormone (TRH) [295]. Parvocellular neurons from the dorsal, lateral, and medial (ventral portion) regions of the PVN also project to other regions of the brain, in particular to the brain stem and spinal cord [316]. Here the parvocellular neurotransmitters/neuropeptides modulate somatomotor-behavioral and autonomic circuitry; for example, CRF axons terminate in regions such as the locus coeruleus, where the peptide is reported to interact with noradrenergic neurons [72,332], VP neurons project to autonomic nuclei in the brainstem and spinal cord, and are involved in cardiovascular control [20,259,300], while parvocellular OT released in the hindbrain/spinal cord influences nociception, gastric reflexes, cardiovascular responses, yawning and penile erection [98,163,204,259]. Moreover, dendritically released neuropeptides from magnocellular perikarya may act locally or diffuse away from the PVN/SON, contributing to the central OT and VP pool [204]. It has been proposed that centrally released OT and VP from dendrites and/or parvocellular projections modulates behavior e.g., maternal and affiliative behavior, sexual behavior, and social recognition [107,204,309].

The PVN and SON are subject to regulation by many brain regions including the hindbrain/brainstem, limbic regions, lamina terminalis system, and other hypothalamic nuclei, and also from chemicals/hormones such as estrogen and CORT that can pass the blood brain barrier [63,114,223,298,316]. As such the PVN/SON is modulated by a considerable array of neurotransmitters and hormones. For example, neurons of the PVN/SON are immersed in glutamatergic and GABAergic terminals that provide major stimulatory and inhibitory tone, respectively. Input from the hindbrain to the PVN and SON includes catecholamine and serotonergic afferents from the ventral medulla, and catecholamine projections from the nucleus of the solitary tract and locus coeruleus, all of which may co-express additional neurotransmitters or neuropeptides [298,316,331]. The PVN and SON also receive efferents from the subfornical organ, and those projecting to the PVN have been shown to contain angiotensin II [82], while neuropeptide Y (NPY)- and pro-opiomelanocortin (POMC)-expressing neurons projecting to the PVN from the arcuate nucleus [283] are essential for the complex control of feeding behavior [285]. Further, parvocellular and magnocellular neurons of the PVN/SON coexpress many neuroactive substances (e.g., CRF, galanin, cholecystokinin, enkephalin, and vasoactive intestinal peptide (VIP)) that may have a paracrine/autocrine action on PVN/SON neurons [39,47]. Importantly, in response to various physiological conditions the PVN and SON exhibit a considerable degree of morphological (e.g., glial cell remodeling [115]) and functional plasticity. This can manifest itself by changes in neuronal excitability [84,322] that may be accompanied by enhanced co-expression of certain neuropeptides (e.g., VP in CRF pPVN neurons after stress exposure [2,39]) and/or altered neuro-transmitter/peptide release [42,202].

**Fig. 1:**
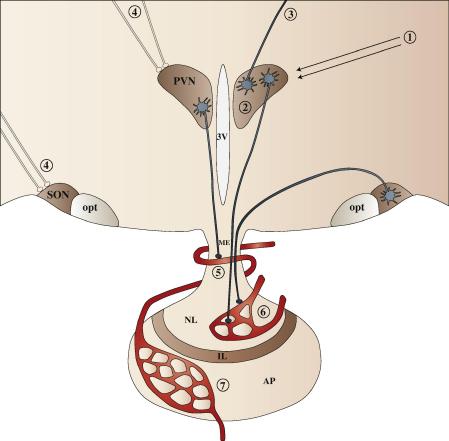
Schematic diagram representing the possible roles of GPCRs in modulating PVN/SON activity. (1) Hormonal signals from peripheral blood may regulate PVN/SON activity directly (substances such as neuropeptide Y and orexin A [153,154] that can pass through the blood brain barrier activating GPCRs), or indirectly (e.g., activation of GPCRs in the circumventricular organs by humoral factors like cytokines, or neuropeptides such as angiotensin II [92] can regulate neurotransmitter/neuropeptide-expressing neurons projecting to the PVN/SON). (2) Local release of neurotransmitters from within PVN/SON (e.g., from dendrites) that act on GPCRs may have potential autocrine/paracrine effects on PVN/SON neurons e.g., priming of OT neurons by dendritically released OT in parturition/lactation [204]. In addition, neurotransmitters (e.g., melatonin, GnRH [335]) released from alternate (ventricular bordering) brain regions may reach the PVN via the ventricular system, and act to regulate neuronal activity via GPCRs. Similarly, dendritically released OT and VP from PVN neurons may permeate into the cerebrospinal fluid of the third ventricle and diffuse to, and act on, GPCRs in distant brain regions [335]. (3) GPCRs may modulate the activity of neurons that project away from the PVN/SON (e.g., parvocellular PVN projections to the hindbrain), acting directly on perikarya within the PVN/SON and/or at the nerve terminals in different brain regions. (4) GPCRs may be present on/or near the nerve terminals of interneurons (e.g., GABAergic and glutamatergic) within the PVN/SON and/or neurons originating from other regions (e.g., alternate areas of the hypothalamus, hippocampus, amygdala, brainstem), that synapse with PVN/SON soma, possibly regulating postsynaptic neurotransmitter release or acting directly to stimulate/inhibit PVN/SON neuronal activity. (5) GPCRs present in the external zone of the median eminence could modulate the secretion of CRF/OT/VP from parvocellular neurons into the portal blood stream, and GPCRs in the pituitary could have a direct effect on hormone release e.g., regulate VP/OT release from the neural lobe (6) and ACTH (amongst other neuroendocrine hormones) from the anterior lobe (7). PVN, paraventricular nucleus; SON, supraoptic nucleus; ME, median eminence; NL, neural lobe; IL, intermediate lobe; AP, anterior pituitary; opt, optic tract; 3 V, third ventricle.

The defined cytoarchitecture of the rat PVN and SON and the features of the neurons contained within and projecting from these nuclei (e.g., large size of magnocellular cells; accessibility to experimental manipulation with reference to the SON in particular; physiologically defined outputs) make these brain regions excellent models to investigate GPCR function. As shown in Fig. 1, GPCRs can modulate PVN/SON activity at a number of levels.

## 3 Structure and function of GPCRs

In the CNS, GPCR ligands function mainly as slow neuromodulators rather than the fast, small molecule neurotransmitters such as glutamate and GABA acting on ionotropic receptors. Traditionally GPCRs are regarded as plasma membrane-bound receptors, although some are not highly expressed on the cell surface (e.g., human gonadotropin releasing hormone (GnRH) receptor [83] and the putative estrogen receptor GPER [266]), and increasing evidence suggests that functional GPCRs may be found in intracellular compartments such as the endoplasmic reticulum, golgi apparatus, nuclear membrane and even inside the nucleus itself [26,43,51,264,266]. The structure/function relationship of GPCRs has been covered extensively and we refer the reader to a number of excellent recent reviews (e.g., [59,128,274]). At a basic level all GPCRs have a common structure of seven membrane-spanning domains, flanked by an extracellular amino terminus and an intracellular carboxyl terminus. The transmembrane (TM) domains are formed by seven hydrophobic α-helices linked by alternating extracellular and intracellular loops. Much of what we know about GPCR ligand-binding pockets and G protein-coupling domains comes from site-directed mutagenesis and chimeric receptor studies assisted on occasions by computational modeling (e.g., [95,349,351]). In general, this large body of work has revealed that the extracellular and TM domains are responsible for ligand binding, while intracellular domains dock and activate G proteins, and anchor the receptor to the membrane. The intracellular domains are also targets for post-translational modification such as phosphorylation which play a critical role in GPCR ‘memory’ resulting in reduced (desensitization) or augmented (sensitization) responses [90]. GPCR desensitization in response to agonist stimulation is common to nearly all GPCRs, and typically involves GPCR kinase (GRK)- or other kinase-induced phosphorylation of the activated GPCR, and recruitment of β-arrestins to uncouple the receptor from its associated G protein, and targeting of the GPCR for endocytosis by linking it to ‘adaptor’ molecules such as clathrin [90,326]. The amino terminus invariably contains N-linked glycosylation sites involved in intracellular receptor trafficking, membrane expression and ligand binding [350], and the carboxyl terminus hosts sites for palmitoylation to facilitate interaction with the membrane, and together with phosphorylation sites have roles in receptor dimerization and internalization, and intracellular signaling [134]. GPCRs can be grouped into four main classes based on shared sequence motifs: (1) Class A (rhodopsin-like), the largest GPCR class that includes the earliest GPCRs cloned (β_2_-adrenoceptor [69], acetylcholine M_1_ [170]) and the aminergic, olfactory and majority of neuropeptide GPCRs; (2) Class B (secretin-like), comprising calcitonin, glucagon, CRF and parathyroid hormone receptors that have a characteristic long amino-terminus tail containing three conserved disulfide bonds; (3) Class C (metabotropic glutamate-like) with an amino terminus consisting of two lobe-like structures that resemble that of a ‘venus flytrap’ (e.g., metabotropic glutamate, calcium-sensing, and GABA_B_ receptors); and (4) Frizzled/Smoothened receptors, which are the sole members of the fourth group. Frizzled receptors have a large amino terminus, and are important in embryonic development and adult tissue homeostasis, while Smoothened receptors contribute to the hedgehog signaling system, and are involved in embryogenesis and tumorigenesis [111,246].

For many years the prototypical reference for GPCR organization in the lipid bilayer has been based on rhodopsin and its high-resolution X-ray crystallographic structure [248]. The successful crystallography of hormone-binding GPCRs is a significant breakthrough in GPCR research that has required the recombinant generation of high levels of GPCR protein, enhancing their stability (e.g., using stabilizing ligands) and structural modifications to encourage crystal formation. The structures of a number of Class A GPCRs (e.g., β_1_- and β_2_-adrenoceptors [52,263]; adenosine A_2A_ receptor [142] and the minimally active conformation of opsin (the ligand-free form of rhodopsin) [249]) have now been elucidated, confirming that essentially all Class A (and by extension Classes B and C) GPCRs possess seven membrane-spanning helical domains arranged in a bundle with a cytoplasmic eighth helix immediately following TM7. One insight from the small number of X-ray crystallography studies to date is that each subfamily of GPCRs possesses its own unique mode of natural ligand binding reflecting their unique structure. In the rat genome there are a total of 356 non-chemosensory GPCRs of which 132 are classified as orphans [111].

GPCRs are coupled to G proteins that are comprised of three subunits: Gα, Gβ and Gγ. Stimulation from physiological, environmental or experimental signals provokes a conformational change in the receptor-7TM structure, catalyzing the replacement of GDP for GTP on the Gα subunit. Subsequently, Gα detaches from Gβγ to create two separate components that can activate a multitude of intracellular signaling pathways e.g., Gα may increase adenylyl cyclase activity, whilst Gβγ can independently act to stimulate phospholipases and MAPK/ERKs, and activate ion channels. Heterotrimeric G proteins are encoded by a family of related genes that comprises 21 Gα, 5 Gβ and 12 Gγ mammalian genes, giving rise to a variety of G protein combinations [70]. They are categorized into four main groups according to the structure and function of the α subunit: Gα_s_, Gα_q/11_, Gα_i_, and Gα_12/13_ [246]. Gα_s_ typically activates adenylate cyclases that catalyze the production of cAMP from ATP, stimulating PKA activity. Gα_q/11_ couples to and activate phospholipase Cβ (PLCβ), and increases intracellular Ca^2+^ and PKC activity. Gα_i_ often inhibits adenylate cyclase activity, impeding cAMP synthesis, as well as activating G protein-coupled potassium channels. The remaining group of G proteins is the Gα_12/13_ family that regulate the small G protein Rho through Rho-specific guanine nucleotide exchange factors [254].

A typical feature of GPCR signaling is that by activating a cascade of signal transduction mediators the signals can be amplified. Cross-talk between GPCRs, or GPCRs and other proteins at the cell surface (e.g., via oligomerization) and in the cytoplasm (e.g., via convergent signaling pathways such as G_s_/G_q_/G_i−_ and receptor tyrosine kinase-activation of MAPK–ERKs) can modify GPCR-mediated signaling. In addition, GPCRs vary in their specificity for activating/coupling to the G protein subtypes with some activating only one Gα subtype while others are more promiscuous and couple to a number of Gα proteins to activate multiple intracellular signaling cascades. The ability of GPCRs to activate more than one class of G proteins can depend on receptor density, the nature of the ligand (different responses to two ligands can confer ‘functional selectivity’), tissue distribution, and on its localization within specialized compartments of the plasma membrane which may depend on whether the GPCR is active in a monomeric or oligomeric form [158,219,355,371]. One facet of GPCR intracellular signaling that should not be overlooked is that some GPCRs can activate both G protein-dependent and G protein-independent pathways. For example, angiotensin II AT_1A_ receptor-triggered transactivation of the EGFR, and β-arrestin-dependent and -independent AT_1A_ receptor internalization can take place independently of G protein activation [81]. In addition, AT_1A_-mediated activation of ERK features both G_q_- and β-arrestin-dependent pathways [181], while β_2_-adrenoceptor stimulation of the MAPK–ERK pathway is G_s_-coupled and Gα-independent/tyrosine kinase Src-dependent at low and high concentrations of stimulating agonist, respectively [313]. Overall it is apparent that GPCRs dynamically interact with numerous associated proteins as part of a tightly regulated signaling network, and this interaction in different tissues reflects the types of signaling components within a given cell and the receptor’s physiological role.

## 4 GPCR expression in the PVN and SON

Over 90% of non-chemosensory GPCRs are expressed in the mouse brain with a large proportion (82% of those examined by RT-PCR) expressed in the hypothalamus [334]. The profiles of the vast majority of GPCRs are unique, and when combined with brain region-specific intracellular signaling component expression (see Section 4.1 below), yield thousands of GPCR signaling combinations for the modulation of physiological processes. Some GPCRs even appear to be relatively confined to the CNS, although it is rare to find evidence of CNS exclusivity if techniques such as reverse-transcription (RT)-PCR or EST profiling (e.g., see http://www.ncbi.nlm.nih.gov/UniGene/) are used.

After a flourish of research in the 1980s/early 1990s localizing GPCRs by receptor autoradiography (ARG), more recent developments in immunohistochemistry (IHC), *in situ* hybridization histochemistry (ISHH), transcriptome approaches such as DNA microarrays and *ex vivo/in vivo* electrophysiological methods have greatly contributed to our understanding of the regulation of the PVN/SON activity by GPCR-based signaling. The PVN and SON are highly vascularized and blood vessel elements and ‘supporting’ cells such as glial cells express GPCRs. In the following sections we focus on the expression of GPCRs in neurons of the rat PVN and SON, although it should be emphasized that this has not been conclusively demonstrated in all studies, e.g., receptor ARG and DNA microarray experiments.

### 4.1 Intracellular signaling components in the PVN/SON

We would expect that cells in the PVN and SON are equipped with the appropriate sets of receptors and various intracellular signaling components to sense and respond to perturbations in homeostasis. Regulation of both GPCR signaling molecules and GPCRs themselves (see Section 5) will contribute to the adaptive responses of the PVN and SON. The distribution of various GPCR cytoplasmic signaling components in the PVN/SON has not been extensively studied, although the function of various G proteins and other intracellular signal transduction mediators involved in GPCR-mediated effects has been implicated in a number of studies. Immunoreactive (ir)-Gβ1–5 and γ3 are expressed at low levels in the rat PVN and the expression of the various β subunits is increased by repeated restraint [178,191]. While ISHH studies suggest that there are very low levels of PKC subunit mRNAs in the rat PVN and SON [32], other studies point to the expression of ir-PKC-δ in neuronal cell bodies in the PVN/SON, and ir-PKC-ϕ in PVN fibers [138], and phosphatidylcholine specific phospholipase C-mediated VP release from the hypothalamus *in vitro* appears to involve PKC activation [343]. Osmotic stimulation increases Gα_i_ and Gα_s_ mRNAs in the magnocellular PVN and SON, and cAMP in the SON [368], while Gα_q_ appears to participate in high-salt induced VP secretion in Dahl salt-sensitive rats [336]. Of the nine adenylate cyclase isoforms only type 2 appears to be strongly expressed in the PVN and SON [227]. Elevated cAMP within PVN/SON neurons may stimulate cAMP response elements in gene promoters to alter neuropeptide (or GPCR) gene transcription, exemplified by studies showing cAMP-driven CRF and VP gene expression in the PVN [9,42,139,356]. The PVN and SON also express mRNAs for numerous members of the regulators for G protein signaling (RGS) family including RGS4, 5, 7, 8 and 9 [101] – these proteins modulate the function of the Gα and Gβ subunits, and the gene expression of at least one member (RGS4) in the PVN has been shown to be downregulated by repeated stress [237]. Other studies suggest that the spatial distribution of some signaling molecules within the PVN may be functionally relevant, e.g., RGS4 and Gα_q/11_ mRNAs are found in both pPVN and mPVN neurons while RGS7 gene expression is confined to the mPVN [292].

Gene expression profiling [124] considerably extends early studies [194] cataloguing some GPCR-related signaling molecules in the PVN and SON. A plethora of gene transcripts relevant to GPCR signal transduction has been revealed (see Supplementary Tables 1 and 2), including those encoding the relatively abundantly expressed Ca^2+^-binding calmodulins, endocytosis adaptor molecules dynamin and clathrin, various RGS and G proteins, and a number of PKC, phospholipase C and D, and cAMP isoforms. While the presence and anatomical distribution of the majority of these transcripts has not been validated by other criteria (e.g., IHC, ISHH, RT-PCR), the data indicates that the PVN and SON express a considerable network of intracellular signaling proteins that could potentially be enlisted upon GPCR activation.

### 4.2 Detection of GPCR proteins by receptor autoradiography (ARG)

**Fig. 2:**
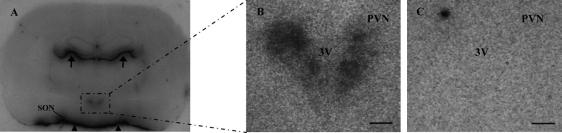
Receptor autoradiographical localization of the apelin APJ receptor with the APJ agonist ^125^I-(Pyr^1^)apelin-13 in 20 μm sections of adult male Sprague–Dawley rat brain. APJ ARG was performed with modifications of a previously described procedure [156]. Sections were incubated with 0.5 nM ^125^I-(Pyr^1^)apelin-13 (Perkin Elmer, Cambridgeshire, UK) alone (A,B) or in the presence (C) of 1 μM cold ligand ((Pyr^1^)-apelin-13; Bachem, Germany), and exposed to emulsion-coated X-ray film (Amersham Hyperfilm ^3^H) for 25 days which was then developed manually as per the manufacturer’s instructions. APJ binding sites in brain structures coincides with the mRNA distribution (see Fig. 4H in [243]) – binding in the SON is obscured by the intense labeling of the basal (free) surface of the hypothalamic diencephalon (arrowheads). Arrows point to APJ binding in the dorsal surface of the thalamus. B and C are a magnification of the PVN – labeling (B) present in both the magnocellular and medial parvocellular PVN is displaced by excess cold ligand (C). Scale bars, 200 μm.

The advent of molecular biological techniques that resolved the genetic fingerprint of GPCRs led to the popular use of GPCR antibodies – generated from predicted protein sequences of cloned GPCR DNA sequences – to visualize GPCR protein expression by techniques such as IHC. Prior to this receptor ARG was a popular tool to delineate GPCR binding sites in brain and peripheral tissues since it provided the ability to anatomically resolve receptor protein expression and to quantitate receptor levels. The method can give higher (cellular) resolution if tissue sections are apposed against emulsion-coated coverslips [365] rather than against X-ray film. A major consideration when using receptor ARG is that not all pharmacologically defined binding sites necessarily represent physiologically active receptors – in a famous ‘caveat’ to those undertaking receptor studies, Cuatrecasas and Hollenberg [62] described how iodinated insulin appears to bind with high affinity to non-biological surfaces like talc with characteristics – except ‘biological activity’ – that are commonly attributed to specific hormone–receptor interactions. Moreover, while radiolabeled ligands may bind ‘functional’ (capable of binding an agonist) GPCRs they may not bind to the entire receptor pool e.g., they may only bind to high affinity binding sites, receptor–G protein interactions critical for agonist binding may be disrupted during the receptor ARG procedure, and ‘immature’ GPCRs that have not been post-translationally modified and/or possess the requisite tertiary structure, or degraded GPCRs may not bind the ligand. Other limitations of the technique include the masking of binding sites by endogenous ligand, although this is usually minimized by buffer pre-washes prior to ligand incubation. Receptor binding studies on tissue homogenates (infrequently if ever used for GPCR expression in the PVN/SON) or receptor ARG are critically dependent on the specificity and selectivity of the radiolabeled ligand employed – high affinity radioligands selective for a particular GPCR subclass are not always available. Specific binding is defined as for receptor binding assays on tissue homogenates, and includes diminution of bound radioactivity by the addition of excess cold ligand and establishing a pharmacological profile using closely- and distantly-related compounds. Knockout mice (providing the distribution of GPCRs in rat and mouse are the same) are an invaluable addition in validating radioligands for a specific receptor. Detection of low amounts of protein also depends on the sensitivity and specific activity of the radioligand, e.g., iodinated versus tritiated ligands can be used for shorter exposure times against film but offer lower resolution. An example of receptor ARG for the apelin APJ receptor is shown in Fig. 2. In this particular case there is almost a perfect overlap between APJ binding sites and APJ mRNA as shown by ISHH [243] – such a strong correlation between receptor protein and mRNA is not always the case since GPCR mRNA is present primarily in cell bodies whereas the corresponding protein may be present at distant sites, e.g., on projecting axon terminals. IHC and ultrastructural studies are mandatory to address the potential mismatch between GPCR protein and GPCR mRNA in the brain.

The list of the 25 GPCR subfamilies detected in the PVN/SON by receptor ARG is shown in Supplementary Table 3. The number is likely incomplete since not all the literature covering GPCR receptor ARG in the brain encompasses the pertinent hypothalamic levels, and even when the relevant brain levels have been included in some studies it is often difficult to ascertain if binding is above background levels. Critically receptor ARG (and other receptor protein or RNA detection techniques) does not directly inform about GPCR function. This can be addressed in part by ‘functional’ ARG with [^35^S]GTPγS to map region-specific, GPCR ligand-dependent activation of G proteins [112,303]. Although it has not used extensively in the PVN and SON, [^35^S]GTPγS binding ARG has demonstrated ‘active’ neuropeptide Y_1_ and Y_2_ [286], and cannabinoid CB_1_ [121] binding sites in the PVN. Positron emission topography (PET) is an alternative imaging technique to visualize GPCRs non-invasively in the PVN and SON *in vivo*; while the technique is relatively low resolution and there is a dearth of suitable GPCR ligands for such studies, there are a few publications (e.g., 5-HT_1A_ receptors in the rat PVN [12]) indicating that this approach may be a useful adjunct to receptor ARG studies in the future.

Receptor ARG rarely has the sensitivity or resolution of IHC. Moreover, in the absence of selective ligands to define a GPCR family in the PVN/SON, IHC and/or ISHH with subtype-selective antibodies and DNA/RNA probes, respectively, can elaborate a specific GPCR receptor subtype.

### 4.3 Immunohistochemistry (IHC) to visualize GPCR expression

Since GPCR-specific and -selective ligands are not available for all GPCRs, antibodies have been a popular option to detect many GPCRs. IHC employing primary GPCR antibodies traced with secondary antibodies to permit fluorescent or chromogenic detection of ir-proteins is a valuable technique to localize GPCR expression in sections of the PVN and SON, offering a far greater lateral and axial resolution than receptor ARG. A major consideration in all GPCR protein and mRNA detection techniques is specificity. The GPCR field is awash with reports of GPCR antibodies that don’t ‘work’ between laboratories, those that have stopped working after new batches were purchased, and those that give no staining. For antibodies in particular and the IHC method in general, the evaluation of specificity has provoked numerous comments in the past with many concluding that antibody specificity is a difficult criterion to fulfil [315]. There are well-established controls for IHC procedures, including the absence of staining when the antibody is pre-absorbed with the immunizing antigen, although this only proves that the antibody bound the added antigen and not that the antibody is ‘specific’ for the GPCR, and the presence by Western blotting of the appropriate GPCR molecular sizes which may correspond to post-translationally modified and/or oligomeric forms. However, other points related to antibody use and storage (e.g., possibility of ‘carrier’ antibodies contributing to staining patterns; tendency of antibodies to form aggregates at 4 °C; potential instability of immunoglobulin fractions or affinity-purified antisera; prolonged storage times between fixation, sectioning and staining; inefficient blocking of immunoglobulin Fc receptors (which are present in the PVN/SON [124]) – e.g., see [348] tend to be under-appreciated and often overlooked, and can lead to increased non-specific, or variable or complete absence of specific staining. Alterations in IHC staining patterns between different antibody batches (either from different animals or different bleeds from the same animal) can often be explained by the inherent characteristics of the normal immune response, e.g., decreasing antibodies titers, or high-affinity antibodies present in an early bleed may be replaced by high-avidity antibodies (perhaps with a lower relative concentrations of specific versus ‘less-specific’ immunoglobulins) as the immune response proceeds. The majority of GPCR antibodies for IHC are raised to short, synthetic GPCR peptides (‘haptens’) usually coupled to a carrier (e.g., keyhole limpet hemocyanin or sometimes bovine serum albumin) to enhance the anti-hapten antibody response, or less frequently to partially purified native or recombinant GPCRs. Invariably the antibodies are a polyclonal mixture (monoclonal antibodies have only been used occasionally (e.g., see [275,288,370]) and directed to regions that are most divergent between different GPCR subclasses, N- or C-terminus moieties being the most attractive targets. Most GPCRs are post-translationally modified [53,326,350], a crucial point in GPCR antibody production since regions that can be potentially glycosylated, phosphorylated or acylated *in vivo* may mask an epitope to hinder antibody recognition. On the flip side, phosphospecific GPCR antibodies can be made (e.g., [330]). Antibodies can also conceivably differentially react to ligand-activated versus unoccupied GPCR conformations, and antibodies raised against denatured GPCR proteins may not recognize the ‘native ‘ (usually fixed) GPCR in tissue sections.

For GPCRs, serious specificity concerns have been raised in a number of articles contesting the reliability of many GPCR antibodies for IHC (e.g., [224]). In contrast the specificity of antibodies to neuropeptides and other cellular constituents are rarely indicted to the same degree, commensurate with the diverse, largely structurally non-conserved nature of GPCR ligands compared with the often, high amino acid homology between different GPCR subtypes. A recent review of studies using antibodies against 19 α_1_- and β_1_-adrenoceptor, acetylcholine, dopamine and galanin receptor subtypes for immunoblotting and IHC concluded that apparent lack of specificity of GPCR antibodies appears to be the rule rather than the exception [224]. Some sensible suggestions for improving GPCR antibody validation have been made [149,224]. These include the reduction of immunostaining following GPCR knockdown using RNA interference (although a lack of knowledge of GPCR mRNA and protein turnover may make this problematic – see Section 5 below) and obtaining similar staining patterns with antibodies against different GPCR epitopes, although it is rare to find studies using two or more antibodies to detect GPCRs by IHC in the PVN and SON (exceptions include the dopamine D_4_ [67] and glutamate mGlu_1_ [161] GPCRs). Similarly, the absence of GPCR immunostaining in GPCR knockouts has also been advocated as a desired IHC control [224]. Assuming that an antibody is truly GPCR-specific in both rats and mice, and there are no species differences in the GPCR distribution between these animals, the absence of immunostaining in tissues from a knockout animal in which the entire GPCR protein coding sequence has been eliminated should serve as an excellent ‘negative’ control in IHC on rat tissues. However, if the knockout targeting construct does not include the relevant protein region to which the antibody was raised, it is possible that the antibody could react to a protein translated in-frame from the targeting construct *in vivo*, and lack of staining is not a foregone conclusion. While we do not necessarily share the outlook that the specificity of most GPCRs is suspect, a review of the literature emphasizes that caution is warranted, especially when using some commercially prepared antibodies [105,256]. We have not endeavored to evaluate the specificity of antibodies used to detect GPCRs in the PVN and SON. However the expression of many GPCRs detected by IHC (see Supplementary Table 4) has been validated by other methods (which also have their own specificity issues).

Individual GPCR numbers per cell are usually quite low in the brain, with lower estimates ranging from 100 to 300 receptors per cell (very low copy number) to upwards of 2000–6000 receptors per cell (around physiological levels for many GPCRs – e.g., see [152] and references therein)). By way of comparison, cells engineered to express recombinant GPCRs can achieve levels of greater than 100,000 receptors in each cell. The threshold of detection for a ‘good’ antibody in IHC is probably in the order of 10–1000 receptors per cell depending on the staining and microscopical method used (e.g., see [54,73]). The detection of ir-GPCRs in cell bodies, axons, dendrites and terminals, and in intracellular organelles such as endosomes, endoplasmic reticulum and the nucleus by IHC with conventional light microscopy can be facilitated by the use of high-resolution optical imaging techniques like confocal microscopy. GPCRs are highly mobile and traffic between different subcellular compartments in the PVN and SON, and are probably dendritically sorted as in other brain regions [269]. For example, IHC has revealed that the tachykinin NK_3_ receptor translocates to the nucleus of VP and non-VP PVN neurons in a stimulus-dependent manner, where it may play a role in transcriptional regulation [110,131].

CNS GPCRs are not particularly abundant proteins and their signals (and non-specific staining) can be enhanced by using modified IHC protocols incorporating tyramide signal amplification (TSA) [25]. Even with improvements in IHC detection, however, it is often difficult to discern whether GPCR staining is associated with the cell surface in detergent (e.g., Triton X-100)-treated sections of fixed tissue, although there are some examples of uniform or punctate staining closely apposed to the plasma membrane (e.g., tachykinin NK_3_ [131]; PTH2 parathyroid hormone [341] receptors). In most cases in the PVN and SON ir-GPCR staining is quite nondescript and apparently found mainly intracellularly, which has important functional implications for some GPCRs that are thought to be active inside the cell (e.g., the putative estrogen receptor GPER [266]). For the majority of GPCRs, an intracellular versus plasma membrane distinction may ‘simply’ reflect the detection of mature GPCRs in the endocytic pathway and/or immature GPCR pools (presumably functionally inactive) yet to be presented to the plasma membrane. In a few instances light microscopic studies have been reinforced by higher magnification immuno-electron microcopy, e.g., in the PVN and SON ir-GABA_B1_ is mainly associated with the endoplasmic reticulum, golgi apparatus and large membrane-bound vesicles, while a small amount of staining is found close to the plasma membrane [268]. The possible functional relevance of ir-GPCR localization in PVN and SON neurons is supported by other studies, e.g., staining for the CB_1_ cannabinoid receptor, a GPCR that inhibits the release of excitatory and inhibitory neurotransmitters in the brain [253], is clearly present in GABAergic terminals and fibers surrounding oxytocinergic PVN neurons [45]. The CB_1_ receptor appears to be synthesized in the PVN and SON [217] but other GPCRs such as the prostanoid EP_3_ receptor [234] appear to be confined to fiber terminals presumably as part of afferent projections to the PVN/SON. So IHC can give some idea of the pre/post-synaptical localization of GPCRs in the PVN and SON.

**Fig. 3:**
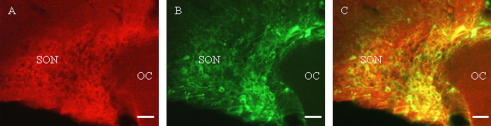
Double label immunofluorescence for GPER and VP in the adult. male Sprague–Dawley rat SON. Images for GPER (A; red) and VP (B; green) immunoreactivity were merged (C; overlap yellow). 50–70% and 40–60% of VP and OT magnocellular neurons, respectively, express ir-GPER [116]. Antibodies and method are described previously [116]. OC, optic chiasm; SON, supraoptic nucleus. Scale bars, 50 μm.

Strong indirect evidence that GPCRs in the PVN and SON may be functionally important also comes from studies where ir-GPCRs have been localized to phenotypically-identified neurons. For example, α_1D_-adrenoceptor [280] and angiotensin AT_1A_ [245] receptors are both located in pPVN CRF-expressing neurons, the 5-HT_1A_/_2A_ [370], apelin APJ [327], chemokine CXCR4 [44], estrogen GPER [31,116] – see Fig. 3.), GABA_B1_/_B2_ [268], κ opioid [299] and tachykinin NK_3_ [110] receptors are expressed in VP and/or OT neurons, whereas the glutamate mGlu_1_ receptor has been identified in both CRF and VP neurons [165]. VP, OT and CRF (and TRH, dopamine, GHRH and somatostatin) neurons in the PVN and SON also express additional neuropeptides that could be co-regulated [39]. The presence of VP V_1A_ receptors on VP neurons [133] and OT receptors on OT neurons [215] suggests that these receptors may act in an autocrine fashion to regulate the release of their own cognate ligands. Moreover, the demonstration that some GPCRs (e.g., apelin APJ [327], estrogen GPER [116], and parathyroid hormone PTH2 [341] receptors) are present on both PVN and SON neuronal cell bodies, fibers and terminals (e.g., in the median eminence or in the posterior pituitary) suggests that GPCRs may act at different locations to alter neuropeptide or neurotransmitter synthesis and/or release (see Fig. 1). Based on its intracellular, and to a minor degree cell surface localization, the estrogen GPER receptor is an example of a GPCR that may be functionally active on or in neuronal cell bodies in the PVN and SON, dendrites, and axonal projections through the internal zone of the median eminence and posterior pituitary nerve terminals [116]. Given the breadth of ir-GPCR distributions in the PVN and SON, and the estimated number of neurons in the PVN and SON (e.g., there are about 1000 and 3000 VP neurons in the rat PVN and SON, respectively, and approximately 1250 OT neurons in both nuclei – [267], it is extremely likely that many GPCRs are co-expressed in individual neurons. In fact, the possible co-existence of two (or more) different GPCRs in the same neuron would support the concept that GPCRs may physically interact (see Section 6 below) in the PVN and SON. However, demonstrating co-expression of two or more proteins in a cell is difficult, although not impossible (see [33,235]) using antibodies raised in the same species to detect non-abundant proteins. In the SON and elsewhere in the brain GPCR co-expression appears to be the case for the two subunits (each a 7TM ‘receptor’) of the GABA_B_ receptor, GABA_B1_ and GABA_B2_ [268], which must heterodimerize for functional GABA_B_ responses [216].

There are a number of mismatches between GPCR protein and mRNA as determined by receptor ARG and/or IHC and ISHH, respectively. For example, binding studies with an iodinated glucagon GLP-1 receptor agonist detect dense labeling in the median eminence and posterior pituitary where there is no GLP-1 receptor mRNA [100,293]. Conversely, GLP-1 receptor mRNA is concentrated in the PVN where only weak binding is observed and where ir-GLP-1 fiber terminals are closely associated with OT- and CRF-expressing neurons [324]. The apparent discrepancies between GPCR protein and mRNA localizations highlight technical issues (e.g., sensitivity) and where GPCR transcription in cell bodies, translation in cell bodies and perhaps axons and dendrites, and transport along axonal and dendritic fibers may occur in the PVN and SON.

### 4.4 *In situ* hybridization histochemistry (ISHH) localization of GPCR mRNA

ISHH was introduced in 1969 [40,91,147] as a method to detect specific mRNAs within cells by hybridizing labeled RNA, cDNA, or short oligonucleotide DNA probes to target sequences in tissue samples. Employing IHC in concert with ISHH can provide converging anatomical evidence to form testable hypotheses and support data on GPCR function in the PVN and SON. High throughput ISHH as advocated for mapping high-resolution gene expression in the brain ([182] – see Allen Brain Atlas @ http://brain-map.org) is usually satisfactory for abundant genes. Apart from a few notable exceptions such as the cannabinoid CB1 receptor gene that is highly expressed in many brain regions [217], most GPCR mRNA(s) are not as abundant as those encoding ionotropic receptors and are visualized usually after weeks–months exposure against X-ray film or photographic emulsion. However, refinements in the ISHH method permit the detection of as few as 10–20 mRNA copies per cell [294], sensitive enough to visualize the majority of the rarest GPCR transcripts, and to compare changes in GPCR gene expression at the cellular level by counting silver grains or at the macroscopic level by image analysis and densitometry with reference to the appropriate autoradiographic standards (as for receptor ARG). ISHH detection sensitivity can also be enhanced by using multiple oligonucleotide probes to different regions of the designated mRNA, or by a number of amplification methods such as TSA (see Section 4.3 above).

Cloning of the mammalian GPCR cDNAs, or identification of GPCR DNA sequences using homology-based searching tools, has provided the platform to map GPCR transcript expression in the brain by ISHH. More often than not ^35^S-labeled antisense RNA probes targeting a large part of the GPCR mRNA (e.g., approx. 300–600 bp RNA probes (riboprobes) for proteins whose coding regions average about 1000–1500 bp in length) are used for optimal GPCR transcript detection: these can be labeled to a higher specific activity, and bind more strongly to target mRNA sequences, than oligonucleotide probes. The use of long riboprobes and even short oligonucelotides (typically 40–48 bp) introduces its own set of problems since hybridization to closely related GPCR subtypes may occur if probes are designed to a relatively well-conserved part of the GPCR mRNA sequence. GPCR-subtype specificity is usually increased if regions such as the 3′-untranslated (UTR) of GPCRs are targeted (providing the G/C content of the probe is not so low to preclude high stringency washes). However, specificity concerns may also be compounded if sense probes used as negative controls for antisense probe binding label the tissue of interest (one definition of ‘non-specific’ hybridization), which is not implausible since over 50% of the mammalian genome can produce transcripts from both DNA strands [155]. Evidence that the complementary DNA strand of a GPCR gene can code for another gene is provided by the study of Foletta and coworkers [85], where a sense VP V_2_ receptor probe which does not hybridize to the V_2_ receptor-expressing kidney [247], detected transcripts for a Rho GTPase activating protein in the brain. It is generally advised to use well-characterized probes (e.g., ones that has been validated by Northern blots, and gives appropriate hybridization patterns in control tissues), or more than one probe (and corresponding sense ‘control’) against a target sequence to minimize erroneous interpretations of ISHH labeling patterns. Our experience and that of many other laboratories using ISHH is that, as in the case of antibodies and IHC, there is often significant variability in the signal/noise ratios for different probes directed to the same GPCR mRNA target.

As outlined in Supplementary Table 4, a large number of GPCR mRNAs have been detected in the cell bodies of PVN and SON neurons. By and large there is general agreement on steady-state GPCR gene expression in the PVN and SON between laboratories but some exceptions are apparent in the literature. For example, while Hurbin and coworkers [132,133] detected VP V_1B_ receptor mRNA and protein expression in the mPVN and SON using short oligonucleotide probes and receptor antibodies, respectively, others found only occasional V_1B_ receptor mRNA-expressing cells in the pPVN using riboprobes directed against the 3′UTR of the receptor [366]. Studies such as these emphasize the importance of probe specificity and the limits of ISHH, and raise questions of mRNA and protein turnover (see Section 6).

**Fig. 4:**
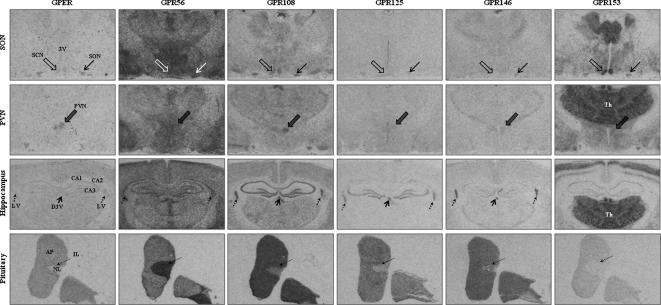
Examples of *in situ* hybridization histochemistry (ISHH) for some orphan GPCR mRNAs, and the putative estrogen receptor GPER mRNA (columns), in sections of SON/PVN/hippocampus and pituitary gland (rows) from adult male Sprague Dawley rats. Images are of 12 μm sections hybridized with ^35^S-UTP labeled riboprobes exposed to film (Amersham Hyperfilm MP) for 6 weeks. As the distribution of the rat GPER mRNA (and protein) has been well characterized [116], and is expressed at low levels in the PVN and SON, the GPER probe acted as a positive (method) control for the orphan probes. A comprehensive mRNA (or protein) distribution for most of the orphans has yet to be described. The GPER probe clearly labels the SON (thin arrow), PVN (filled thick arrow), and anterior and intermediate lobe (long dashed arrow) of the pituitary gland, while no signal is present in the neural lobe. An extremely faint signal is also observed in the SCN (unfilled thick arrow) and CA1/CA2 and CA3 hippocampal regions. The positional arrows of labeled structures shown in the GPER distribution are indicate in subsequent images for the orphan GPCRs. GPR56 transcripts appear to be ubiquitously distributed throughout the sections shown, although a more intense labeling is seen in the SON, CA1–3 and the lateral ventricles (short dashed arrow), and to a lesser extent in the PVN. The intermediate lobe and neural lobe of the pituitary are also intensely labeled with the GPR56 probe, while the anterior pituitary is moderately labeled. GPR108 mRNA is highly expressed in the SCN, SON, PVN, CA1–3 (and dentate gyrus), dorsal third and lateral ventricles, and all three lobes of the pituitary (anterior and intermediate lobe > neural lobe). GPR125 mRNA expression is observed in the dentate gyrus and CA3 of the hippocampus, and the dorsal third and lateral ventricles, with faint expression seen in the PVN/SON. The anterior lobe of the pituitary are strongly labeled with the GPR125 probe with faint, diffuse signal in the intermediate and neural lobes. GPR146 mRNA is present in the PVN/SON, and is highly expressed in the dorsal third and lateral ventricles and anterior lobe of the pituitary, with moderate/faint expression found in intermediate and neural lobes. GPR153 labeling is striking throughout the thalamus, in the SCN, and in several cortical layers, with moderate signal in the PVN/SON, and weak expression in CA1–3 of the hippocampus and anterior pituitary. Sections hybridized with sense riboprobes for all GPCRs as controls showed only background/or were absent of hybridisation signal (data not shown). 3 V, third ventricle; SCN, suprachiasmatic nucleus; PVN, paraventricular nucleus; Th, thalamus; CA1–CA3, CA1–CA3 regions of the hippocampus; D3 V, dorsal third ventricle; LV, lateral ventricle; AP, anterior pituitary; IL, intermediate lobe of the pituitary; NL, neural lobe of the pituitary. The rat orphan GPCR probes were generated by PCR using 125 ng rat genomic DNA (extracted from rat testis) as a template. PCR primers incorporating recognition sequences for restriction endonucleases (underlined) were used to generate products of approximately 300–500 bp in size. All probes were targeted to the 3′-untranslated (UTR) region (or C-terminal into 3′-UTR in the case of GPR125) of each GPCR mRNA. GPR56 primers, upstream: 5′-CCTCTGAATTCGGGGTGCACATGCATGGC-3′; downstream: 5′-CAGACAAGCTTGGAAGATGCTCAGCTCCTA-3′, corresponding to bp2376–2844 of the rat GPR56 gene (Genbank Accession number NM_152242) were used to generate a 469 bp product which yielded a 453 bp probe when digested with EcoRI–HindIII. GPR108 primers, upstream: 5′-ACTTCCCCGAGTTCAGAGATCCGCCTTC-3′; downstream: 5′-AATCAAAGCTTTATGAAGCCCAGGCTCT-3′, corresponding to bp1749–2093 of the rat GPR108 gene (NM_199399) were used to generate a 345 bp product which gave a 329 bp probe following digestion with AvaI–HindIII. GPR125 primers, upstream: 5′-CCAAGGAATTCAGCTGCAGCTGA CCTTGA-3′; downstream: 5′-TTTTTAAGCTTTGGGGAAGGGCAATTTAG -3′, corresponding to bp4198–4549 of the rat GPR125 gene (XM_223485) were used to generate a 352 bp product which gave a 336 bp probe when digested with EcoRI–HindIII. GPR146, upstream: 5′-GGGCCGAATTCCAAGGAGAGGGCCTGACCA-3′; downstream: 5′-TCCTCAAGCTTTAACACTGGTATTTGCGA-3′, corresponding to bp1202–1718 of the rat GPR146 gene (XM_573364) were used to generate a 517 bp product which yielded a 501 bp probe following digestion with EcoRI–HindIII. GPR153, upstream: 5′-CCCCAGAATTCATGCAGACGGAAGAGGC-3′; downstream: 5′-AAGGAAAGCTTGCTCAATAGAACTTGTT-3′, corresponding to 2059–2521 of the rat GPR153 gene (NM_001034855) were used to generate a 463 bp product which gave a 447 bp probe after digestion with EcoRI–HindIII. Recognition sequences for endonucleases facilitated cloning into the RNA-generating vector pGEM4z (Promega, WI, USA), and sense and antisense probes were generated using T7 and SP6 polymerases (antisense: generated with T7 polymerase following linearization with the opposing restriction endonuclease; sense: generated with SP6 polymerase following linearization with the opposing restriction endonuclease) with ^35^S-UTP and the MAXIscript *in vitro* kit (Ambion, TX, USA). The integrity of each probe was verified by DNA sequencing. Rat GPR30 probes were generated as previously described [116]. All *in situ* hybridization experiments were performed as described in detail at http://intramural.nimh.nih.gov/lcmr/snge/Protocols/ISHH/ISHH.html.

Like IHC, ISHH is also amenable to co-expression studies, whether combined with IHC or alternatively used alone to investigate the expression of two distinct transcripts in neuronal cell bodies. For example, 5-HT_2C_ [118], adrenoceptor α_1B_ [65], CRF_1_ [136] and melanocortin MC_4_ [201] receptor mRNAs are predominantly found in CRF neurons, neuromedin U NMU2 receptor mRNA is mainly present in OT neurons [260], and neuropeptide Y Y1 receptor transcripts are co-expressed with TRH mRNA in pPVN cells [160]. Of the 52 GPCRs with known ligands detected in the PVN by IHC, 34 of the corresponding mRNAs have also been detected in the same or independent studies (see Supplementary Table 4). A further 9 orphan GPCR mRNAs are also present in the PVN/SON as detected by ISHH (see Supplementary Table 7). Examples of the ISHH patterns of some of these are shown in Fig. 4. The great majority of GPCRs are expressed in both the pPVN and mPVN (e.g., 5-HT_1A/2A_ [370]; α_1D_-adrenoceptor [280]; apelin APJ [243,327]; calcium-sensing CaS [272]; CRF_1_ [136]; melanocortin MC_4_ [201]; prostanoid EP_1/4_ [244] receptors). Some GPCRs appear to be preferentially expressed in the pPVN (e.g., consistent with regulating stress or autonomic responses), or mPVN (e.g., compatible with regulating water homeostasis or reproductive status) by either IHC and/or ISHH (pPVN: 5-HT_2C_ [118]; angiotensin AT_1A_ [245]; prolactin-releasing peptide PRRP [195] receptors; mPVN: chemokine CXCR4 [44]; neuromedin U NMU2 [260]; κ opioid [299] receptors). One GPCR (neuropeptide FF/neuropeptide AF NPFF1) seems to be PVN-specific in rats [103], although ir-NPFF1 fibers found just dorsal to the SON, as in humans [102], may project to the SON [143] and be responsible for the inhibitory effects of centrally administered NPFF on hypovolemia-induced VP secretion into the blood [10].

### 4.5 Transcriptomic analysis of GPCR expression in the PVN/SON

RT-PCR-based methods have been used to delineate a partial GPCR transcriptome in a number of tissues including mouse heart [228] and brain [334]. Only the odd study has used PCR to detect the expression of an individual GPCR gene in dissected PVN/SON [58,200,291,310]. Large-scale transcriptome analysis of enriched genes, including some GPCR transcripts, has been performed in a number of mouse brain regions including striatum, frontal cortex, hippocampus and amygdala [19,97,183]. Recently DNA microarray-based transcriptomal analysis of the rat PVN, SON, subfornical organ and area postrema, and mouse SON was reported from our laboratories [124,123,122,308]. There are some limitations associated with such ‘global’ studies in rats as highlighted previously [124]. For example, manual rather than laser dissection of PVN and SON was used so a small amount of surrounding tissue such as the 3rd ventricle could have been included in the samples. In addition, most but not all GPCRs with known ligands, or orphan GPCRs are represented on the Affymetrix 230 2.0 rat genome chip interrogated – examples of some ‘missing’ GPCRs include the bombesin BB_3_ receptor and the orphan GPCRs GPR101 and GPR165. Furthermore, some rare GPCR transcripts in the PVN and SON may escape detection, or some probe sets may have failed in some or all of the replicates, thus excluding them from analysis – examples of this are the apelin APJ, estrogen GPER, and VP V_1A_ receptors which are readily detected by receptor ARG, IHC and/or ISHH in the PVN and SON. Bearing these points in mind, we have constructed a list of the GPCRs genes considered present by DNA microarrays in the PVN and SON (Supplementary Tables 4–6). The relative abundance of GPCR transcripts in both hypothalamic nuclei varies from those that are highly expressed such as various GABA_B_ subunits, and neurotensin NTS_2_ and endothelin ET_B_ receptors, to the less highly expressed purinergic P2Y_13_, adenosine A_3_, and metabotropic glutamate mGlu_4_ and mGlu_7_ receptors. About 80% of GPCR transcripts in the PVN are also present in the SON, and approximately 70% and 50% of transcripts for GPCRs with known ligands in the PVN and SON, respectively, has been validated by receptor ARG, IHC and/or ISHH. This includes some GPCR transcripts (e.g., parathyroid hormone PTH_1_ and neuropeptide Y Y_5_ in the PVN/SON) that are towards the lower limits of detection. The GPCR gene lists include 14–16 ‘new’ GPCRs with known ligands, such as adenosine A_2B_, chemokine CXCR3 and CXCR7, lysophospholipid LPA_1_ and S_1_P_1_, metabotropic glutamate mGlu_4_, purinergic P2Y_13_ and protease-activated PAR1 receptors, and 17–21 ‘new’ orphan GPCRs (see Supplementary Table 7) whose localization in the PVN and SON is unvalidated on review of the literature, and which may represent novel targets for future physiological studies. Another interesting feature of the transcriptomic data is that by virtue of multiple oligonucleotide probe sets representing some genes on the array chip, a number of GPCR splice variants appear to be present in the PVN and SON. Alternate splicing of pre-mRNAs is one mechanism for increasing diversity in the transcriptome. Although approximately half of GPCR genes are devoid of introns within their coding sequence, those that possess introns can theoretically undergo alternative splicing and this may have consequences on GPCR functions such as altered pharmacological profiles, constitutive activity and subcellular localization [214]. Examples of GPCRs that exhibit varying degrees of alternate splicing include the GABA_B1_ subunit [345], NOP opioid [361], metabotropic glutamate [240] endothelin ET_A_ [113] and parathyroid hormone PTH1 [150] receptors, all of which have potential isoforms identified by DNA microarrays in the PVN and SON. Transcriptome analysis of the PVN and SON also reveals four GABA_B1_ subunit isoforms (a, f, g, j) – and IHC and ISHH studies indicate that at least two GABA_B1_ subunits (B1a and B1b) are expressed in the PVN and SON [89,22]. There are 12 GABA_B1_ variants (a-k including c-a and c-b) in total, the majority of which are secreted forms that may confer functional differences to the GABA_B1/B2_ heterodimer [325].

It is very likely that the number of GPCR genes expressed in the PVN and SON in the DNA microarray studies outlined above is an underestimate, and would be expanded further by transcriptomic experiments on single neurons. High throughput, deep/next generation sequencing (e.g., RNASeq [339]) of single cell cDNA libraries from pPVN, mPVN and SON neurons, similar to that reported for electrophysiologically identified warm sensitive neurons from the anterior hypothalamic pre-optic area [73], would reveal GPCR splicing complexity, rare GPCR transcripts and also those GPCR genes that are co-expressed (and thus are candidates for heterodimerization) in PVN/SON neurons.

### 4.6 Numbers of GPCRs in the PVN and SON: an overview

Embracing the data from the various detection methods outlined above we have arrived at a conservative estimate of the number of GPCRs expressed in the PVN and SON (Table 1). Of the 224 known non-chemosensory GPCRs in the rat genome 101 are present in the PVN (with a further 14 from unvalidated DNA microarrays), and 80 are present in the SON (excluding another 16 from unvalidated DNA microarrays). Interestingly, of the 132 orphan non-chemosensory GPCRs in the rat genome 22 (9 validated) and 24 (9 validated) are present in the PVN and SON, respectively. The GPCRs encompass the vast majority (33 that are activated by different peptide classes from 46 GPCR families in total) of GPCR families excluding chemosensory and orphan GPCRs present in the rat genome (Table 2). The estimate includes a few instances where GPCR ligands appear to have functional effects (e.g., anaphylatoxin, formyl peptide, kisspeptin, leukotriene, melatonin, motilin, platelet-activating factor and trace amine receptors; see Supplementary Table 9) in the PVN/SON but their presence has not been confirmed by any of the detection criteria reviewed. It should also be emphasized that, as far as we are aware, none of the GPCR cDNAs/genes in the PVN and SON have been sequenced. Variations in GPCR sequences and/or potential splicing patterns may have an impact on the function of PVN/SON GPCRs.

## 5 Regulation of GPCR expression in the PVN and SON

There is ample evidence that GPCR expression can be regulated by, and contribute to changes in PVN and SON neuronal plasticity. Levels of GPCRs are determined in part by the rate of receptor protein synthesis, which can be regulated by either transcriptional or post-transcriptional mechanisms. Unless a reserve of “spare” receptors exists, alterations in cell surface or cytoplasmic GPCR levels can significantly influence receptor signaling capacity. GPCR signaling components (e.g., G proteins) themselves are also dynamically regulated [166,232], and ultimately GPCR expression and function is dependent on a host of factors that influence GPCR desensitization (e.g., following chronic activation of many GPCRs), redistribution and degradation. The role of many intracellular signaling molecules (such as GRKs and arrestins) is critical in regulating these processes. RNA regulation is also very complex, with small RNA molecules like microRNAs (miRs) and piwi-interacting RNAs linked to transcriptional silencing, and long non-coding RNAs involved in transcriptional, post-transcriptional (e.g., RNA alternate splicing, translation) and epigenetic regulation [192].

The apparent absence, or low levels of GPCR expression does not preclude the possibility that some GPCRs may be induced by perturbations of PVN and/or SON neuronal function (e.g., change in osmolality, lactation, stress) as in the case of the CRF_1_ receptor [206]. Changes in mRNA levels are usually easier to detect by ISHH compared to changes in protein as measured by IHC, but this obviously depends on when the mRNA is assayed after experimental manipulations, since GPCR mRNA turnover may vary considerably. GPCR mRNA and protein turnover has been primarily established in cell lines expressing native or cloned GPCRs and could be quite different in the PVN/SON microenvironment. Half-lives are highly variable and often cell context-dependent, ranging from around 2–20 h for both GPCR mRNA (e.g., acetylcholine m_1_ [177], α_1_-adrenoceptor [141], α_1_-adrenoceptor [278], β_2_-adrenoceptor [109], leukotriene BLT_1_ [305] receptors) and GPCR protein at the cell surface (adenosine A_1/2A/2B/3_ [164], α_2A/2B/2C_-adrenoceptor [282,353], β_2_-adrenoceptor [71], calcium-sensing CaS [46], cannabinoid CB_1_ [220] receptors). The mRNA turnover for a number of GPCRs is also decreased by agonist stimulation [57,109,141,177], emphasizing the importance of local agonist levels in the PVN and SON in regulating both GPCR mRNA and protein levels. In a few of these studies, in contrast to research on GPCR mRNA expression in the PVN and SON, nuclear run-on experiments (requiring a million cell nuclei or more) were used to confirm that changes in mRNA levels were the result of changes in GPCR gene transcription. Nuclear run on experiments provide a measure of the frequency of transcription initiation and are largely independent of the effects of RNA stability. Interestingly, other studies using hybridization of DNA microarrays with steady-state mRNA versus newly transcribed (nuclear run on) RNA have shown that approximately half of stress-regulated genes in H1299 lung carcinoma cells are due to changes in gene transcription with a similar fraction due to changes in mRNA turnover [77]. A point that may be relevant to possible GPCR co-expression and cross-talk in the PVN and SON is that the angiotensin AT_1_ receptor induces bradykinin B_2_ receptor transcription activation via the phosphorylation of cAMP response element binding protein (CREB) and assembly of p-CREB on the B_2_ receptor promoter in kidney collecting duct cells [287].

An alternative method to look at gene transcription rates, and one that is particularly amenable to tissue sections of PVN and SON, is to examine heteronuclear (hn)RNA levels. The binding of probes specific for introns in RNA-coding region of genes can be used to quantify hnRNA levels as an indirect measurement of the transcription rate of genes in response to a particular stimulus. For GPCR genes that contain multiple introns care must be exercised in choosing which introns to target because they can be excised from the nascent pre-mRNA at different rates [174]. ISSH with intron-specific probes has been successfully used to measure hnRNA changes for relatively abundant neuropeptide (e.g., VP, OT and to a lesser extent CRF [120,169,369]) mRNAs, but has not proved particularly useful to assess GPCR transcriptional activity. One exception is the dopamine D_2_ mRNA distribution in the brain where hnRNA levels are (as expected) a fraction of steady-state mRNA levels [87].

There are numerous studies showing that the expression of PVN/SON neuropeptides, in particular VP, OT and CRF, are developmentally regulated [6,16,317], and that their expression can be altered by experimental manipulations [42,2]. In comparison, reports of ontogenetic variations in GPCR expression in the PVN/SON are scarce, with the transcript or protein level, and/or function of a few GPCRs including the angiotensin II AT_1a_ (mRNA present in PVN E19 onwards [241]), neuropeptide Y_1_ (mRNA present in PVN P2 onwards coincident with a significant increase in NPY-containing fibers innervating the nucleus [106]), and melanocortin MC_4_ (mRNA present in PVN and SON at E18 and P27, respectively, approximating the appearance of melanocortin binding sites [162,193]) changing developmentally. At least one GPCR in the PVN and SON is also diurnally regulated – α_2_-adrenoceptor expression in the PVN peaks at the onset of dark (when CORT levels are highest) whereas in the SON the reverse diurnal pattern is observed [144].

There have been many studies using receptor ARG, IHC or ISHH to demonstrate alterations in GPCR expression by pharmacological or physiological manipulations. More recently, transcriptome approaches have established that dehydration alters the levels of transcripts encoding the cannabinoid CB_1_, GABA_B1j_, melanocortin MC_4_, protease-activated PAR1 and somatostatin sst_3_ receptors in the rat SON [124]. Changes in GPCR protein and mRNA levels in the PVN/SON in response to agonist or antagonist administration, or physiological perturbations such as adrenalectomy, salt-loading, dehydration, lactation, gestation and stress are commonly less than twofold, but 8–10-fold or higher increases in GPCR mRNA have been reported in some instances – e.g., for the apelin APJ receptor [242] (see Supplementary Table 8). Invariably gene or protein expression has been imaged over the entire PVN and/or SON, so any change in cell-to-cell GPCR expression is often obscured. Importantly, since the vast majority of studies investigate a single experimental time point, it is surprising to note how often it is assumed that changes in GPCR mRNA reflect changes in GPCR protein levels and perhaps receptor function. That this may not always be the case is emphasized in studies where the correlation between mRNA and protein levels has been investigated using transcriptomic- in conjunction with proteomic-approaches. For example, in kidney inner medullary duct cells a large number (approx. 1/3) of proteins that showed significant changes in abundance in kidney inner medullary collecting duct cells following challenge with dDAVP (desmopressin; a VP V_2_ receptor agonist) did not show a changes in the corresponding mRNA species (measured by interrogating DNA microarrays) [159]. While this result relies heavily on the quantitative accuracy of the methods used, it conceivably highlights an important role in post-transcriptional regulation of protein abundance, and also obviously reflects the dynamics of mRNA versus protein turnover. Impressively, given that the half-life of GPCR mRNA or protein is usually not known, a number of studies have combined receptor ARG (or in some cases IHC) with ISHH to show that alterations in GPCR mRNA levels in the PVN/SON are associated with changes in the corresponding GPCR protein. A few examples of this are the increases in angiotensin AT_1A_ receptor after antagonist administration [210,346], and cholecystokinin CCK_1_ and CCK_2_ [125,222], and galanin GAL_1_ [41] receptors following osmotic perturbations.

There are other, largely unexplored and speculative ways in which GPCRs in the PVN and SON could possibly be regulated. One such mechanism is microRNA (miR)-mediated post-transcriptional regulation. There is substantial evidence that the 3′-UTR of proteins can affect mRNA stability and is involved in regulating gene expression at the post-transcriptional level, and in the case of some GPCRs such as the opioid receptors the length of the 3′-UTR influences receptor protein level [358]. MiRs are short, single-stranded non-protein coding RNAs that tend to suppress target gene expression by binding to their complementary mRNA sequences usually in introns or exons of the 3′-UTR, and have emerged as crucial modulators of gene expression especially in synaptic plasticity. The feasibility of mIR-mediated GPCR mRNA regulation has been demonstrated by miR-23b inhibition of opioid μ receptor expression [358]. Conversely, opioid μ receptor agonists regulate miR-190 activity [372]. Scanning individual GPCRs for consensus miR binding sites that are conserved between species would be a starting point for studies on the potential role of miRs in regulating PVN/SON GPCRs. It is also becoming increasingly clear that epigenetic control of gene (especially CRF and VP) expression in the PVN is important in the HPA axis response to stress (e.g., see [74,233]). DNA methylation and histone modifications have been shown to coincide with the differential expression of the opioid μ receptors in the brain [135].

As noted previously, some GPCRs have very low expression levels (e.g., <1000 receptor copies/cell) and ultimately the demonstration of a ligand-specific function is paramount. For those GPCRs investigated, a functional response has generally been observed where GPCR binding sites, ir-protein and/or mRNA have been detected in the PVN/SON.

## 6 Functions of GPCRs in the PVN and SON

The actions of numerous neurotransmitters, neuropeptides and hormones in the PVN and SON have been well documented (e.g., [17,63,114,202,204,265,298,316]) and only some salient features will be described here. The tonic and stimulated activity of the PVN and SON is regulated by a number of excitatory and inhibitory neurotransmitters and neuromodulators, including glutamate and GABA, the main excitatory and inhibitory neurotransmitters, respectively, as well as a host of other effectors including angiotensin II, catecholamines, histamine and numerous other neuropeptides that activate GPCRs, and mediators such as humoral factors and nitric oxide (e.g., [37,38,76,140,151,190,298,331]). This regulation can occur directly in the PVN or SON via the effects of neurotransmitters/neuropeptides synthesized within the two nuclei and/or indirectly by interactions with glutamatergic or GABAergic interneurons or afferent projections from other hypothalamic or extrahypothalamic areas that innervate the PVN or SON [82,146,298]. Apparent mismatches between neurotransmitters/neuropeptides and their receptors that are prevalent in the brain [119] may not be such an issue in the PVN and SON where GPCR ligands are available from a number of sources within the nuclei or extra-PVN/SON locations.

In addition to their peptidergic or neurotransmitter phenotypes, neurons in the pPVN, and mPVN and SON have defined electrophysiological characteristics. Classically, under basal conditions OT magnocellular cells are continuously active, whereas the activity of VP magnocellular cells ranges from continuously active to robust phasic to relatively silent [36,204]. Early studies indicated that mPVN and SON neurons have similar electrophysiological properties [11,221,323] whereas pPVN cells exhibited electrophysiological heterogeneity [323]. Neurosecretory neurons concentrated in the medial pPVN have no low threshold spike (LTS) and small T-type Ca^2+^ currents while in non-neurosecretory cells in the dorsal and ventral pPVN the converse is true [207]. In magnocellular cells bursts of activity often characterize periods of enhanced neuropeptide release. For example, during suckling in the lactating rat and in pregnant animals OT magnocellular neurons discharge synchronously to release large amounts of OT into the systemic circulation which is dependent on dendritic OT release [196,204], and VP magnocellular cells increase their firing (and may switch to phasic activity) to release VP following dehydration [185,337] and hemorrhage [338].

The effects of GPCR ligands on PVN and SON neuronal function can be direct or indirect depending upon whether they are administered peripherally, centrally via the circumventricular organs, or intra-nuclei by injection or iontophoretic application. It should be borne in mind that high doses of GPCR agonists may give ‘pharmacological’ rather than physiological responses, especially when compounds are applied in the vicinity of their presumed site of action. GPCR activation in the PVN/SON has been demonstrated in a number of ways. These include increases in neuronal immediate early gene (e.g., c-*fos*) activation, changes in electrophysiological characteristics or neuropeptide mRNA or protein levels (e.g., by ISHH, ICH or content of push–pull perfusates or microdialysates), and alterations in any number of physiological end-points such as plasma VP, OT, CRF and ACTH release, water and energy homeostasis, cardiovascular parameters, nociception and behavior (see Supplementary Table 9 for some examples). The specificity of the ligand–GPCR interaction is usually demonstrated by the inhibition of responses with GPCR-selective antagonists or, as in a few cases, by immunoneutralization with neuropeptide/GPCR antibodies (e.g., for NPFF effects on VP release [364]), and more recently by RNA interference-driven gene silencing that has the added advantage over acute administration of synthetic small interfering RNAs of long-term (days–months) GPCR knockdown if viral, GPCR-specific small hairpin (sh)RNA constructs are employed. The sustainable expression of such constructs obviates some of the problems that may be encountered with long GPCR mRNA turnover rates.

### 6.1 General features of GPCR function in the PVN and SON

With amplification procedures used in various GPCR detection techniques, an important question is what level of GPCR mRNA or protein is physiologically relevant? Radioligand- or fluorescent-ligand binding assays can detect as few as about 50–100 GPCRs per cell (e.g., see [212]) which is sufficient to elicit (although higher levels are probably required to sustain) a signal transduction response in some *in vitro* systems [297]. In PVN and SON neurons *in vivo* some GPCRs may be clustered to concentrate their levels at pre- or post-synaptic sites. A number of studies underscore the differences in the sensitivity/specificity of detection techniques used between laboratories, and highlight the importance of obtaining (specific) functional GPCR responses. For example, while VP V_2_ receptor mRNA was not detected in the PVN or SON by nested PCR in one study [132], V_2_ receptor mRNA (by PCR on RNA obtained from 20 neurons), V_2_ receptor protein and apparent functional responses have recently been reported in isolated cells from the SON [281]. Similarly, the lack of angiotensin II AT_1A_ receptor gene expression in the mPVN by ISHH in some studies (e.g., [186]) appears at odds with the AT_1_-type pharmacological responses observed by electrophysiology in PVN slices [176]. Furthermore, prostanoid EP_3_ receptor electrophysiological responses have been observed in the SON [290] where no ir-EP_3_ receptor cell bodies or fibers have been found [234].

It is possible that some of the GPCR effects in the PVN or SON are spurious or redundant in nature, since it is difficult to envisage that every GPCR we have listed (see Tables 1 and 2) has an important role in co-ordinating control of PVN and/or SON function. We are reminded of a comment attributed to Alfred Gilman a number of years ago: “A typical cell has perhaps 50 different receptors, and the cell doesn’t pay attention to just one receptor at a time. How does it know how to interpret the signal from one hormone when it’s listening to 45 other ones at the same time? How does the whole signaling system work as a network? That’s what we want to find out” [184]. Functional studies of GPCRs indicate that they do have individual roles in the PVN and SON and are probably key to neurones integrating multiple functions as outline below (see Supplementary Table 9).

Change in the levels of intracellular signal transduction molecules (see Section 4.1) and immediate early gene activation [126,127] are frequently used as indices of neuronal activity in the PVN and SON, and are particularly amenable to cell imaging techniques. For example, agonist-induced increases in intracellular Ca^2+^ or ERK activation have been shown for 5-HT_1A_ [61], α_1A_-adrenoceptor [301], dopamine D_4_ [23], melanocortin MC_4_ [277], purinergic P2Y_1_ [302], OT [24,172,277], VIP/PACAP [66] and VP V_1A_ [104] receptors. These are often accompanied by increases in PVN or SON c-*fos* expression, as demonstrated for many GPCRs such as the 5-HT_2A/2C_ [180], CRF_1_ [251], dopamine D_4_ [23], glucagon GLP-1 [175], melanocortin MC_4_ [157], motilin [359], neuropeptide FF/neuropeptide AF NPFF1 [145], prolactin-releasing peptide PRRP [218,362] and tachykinin NK_3_ [157] receptors. One of the most extensively studied functional aspects of GPCRs in pPVN presympathetic and/or endocrine, mPVN and SON neurons, is their often profound affects on neuronal excitability, examples of which are shown in Supplementary Table 9, and include presynaptic effects mediated via metabotropic glutamate receptors [28,29,284] and endocannabinoids (acting through cannabinoid CB_1_ receptors) [68,277] in the SON, and GABA release in presympathetic pPVN neurons [49,188].

GPCR activation in the PVN/SON can alter neuropeptide or GPCR gene synthesis (e.g., see [3,79,80,167,201,346]) and/or the release of neurotransmitters/neuropeptides from dendrites and/or axon terminals. Local dendritic release of neuropeptides acting in an autocrine or paracrine fashion are likely to be important factors in determining the sensitivity and plasticity of PVN and SON neurons to their multitude of inputs [173,202,204,231]. Dendritic peptides may also regulate local blood flow [5] and have local (e.g., OT is anxiolytic via the PVN OT receptor [333]) and distant effects on behavior [204]. Examples of GPCRs that modulate neuropeptide release, typically performed in studies measuring VP and/or OT release from large magnocellular cells, rather than CRF release from the smaller pPVN neurons, include the α_1_-adrenoceptors (inhibit intra-PVN hypoxia-induced CRF release [50]), apelin APJ (increases firing rate of VP neurons and VP dendritic release [327]), histamine H_1/2_ (increases dendritic release of OT via stimulating noradrenaline release [18]), melanocortin MC_4_ (increases Ca^2+^ in OT neurons; stimulation of dendritic, and inhibition of terminal OT release [204,276]), κ opioid (locally released dynorphin inhibits VP neurons and is essential for expression of VP neuron phasic activity; inhibits VP terminal release [34,36]), VIP/PACAP (stimulates somatodendritic and terminal VP release [208,289]), VP V_1A_ (acting on autoreceptors to excite and inhibit quiescent and phasic VP neurons, respectively [35,203,204]), and OT (stimulates dendritic OT release via OT receptor; inhibits OT neurons by increasing endocannabionoid inhibition of glutamate release [204]) receptors.

The release of neurohypophysial hormones from posterior pituitary nerve terminals, and CRF and other pPVN products from the median eminence into the anterior pituitary portal circulation is often reflected by increased circulating levels of VP and OT, ACTH (due to the action of CRF, VP and other ACTH secretagogues) and CORT, and thyroid hormones, and in changes in water homeostasis (principally brought about by altered VP secretion) (Supplementary Table 9 gives some examples). GPCRs can also modulate autonomic functions by activating secretory and non-secretory pPVN neurons. For example, central or intra-PVN administration of a number of GPCR ligands results in orexigenic (e.g., ghrelin [291], galanin [171], and NPY agonists via Y_1_ and Y_5_ [151] receptors) or anorexigenic (e.g., via CRF_1_ [117], melanocortin MC_4_ [94], neuropeptide S NPS [78], and neuromedin U [357] receptors) effects, and alter cardiovascular parameters (e.g., angiotensin AT_1_ [13], CRF_2_ [189], tachykinin NK3 [320], and urotensin II UT [342] receptors), nociception (e.g., α-adrenoceptors [374]), body temperature (e.g., acetylcholine muscarinic receptors [319]), and penile erection (e.g., dopamine receptors [312]).

### 6.2 Possible GPCR co-expression in the PVN and SON

We do not know how many GPCRs are co-expressed in PVN/SON neurons, and different complements of GPCRs may be expressed in subsets of neurons such as magnocellular VP cells with different basal electrical activity, magnocellular neurons with different neuropeptidergic phenotypes, mPVN versus pPVN neurons, or pPVN endocrine versus non-endocrine neurons. However, extrapolating from the study on single warm-sensitive neurons (transcriptomic analysis gave 168 non-olfactory GPCRs of which 27 are orphans [73]) suggests that the number of co-expressed GPCRs is likely to be larger than the number of co-expressed neuropeptides. At least 20 different neuropeptides are co-expressed in magnocellular VP or OT neurons [39] but the extent of the total overlap is unknown. The co-expression of GPCRs raises the question of possible functional consequences of receptor oligomerization in the PVN and SON. The formation of functional GABA_B_ receptors from two GABA_B_ subunits is an example of GPCR heterodimerization that we know occurs in the PVN and SON [373]. Of the GPCRs listed in Supplementary Table 4, there is also a high degree of colocalization of 5HT_1A_ and 5HT_2A_ in OT and CRF neurons in the PVN where activation of one receptor subtype may induce the desensitization of the other [370]. There are many examples of apparent GPCR homodimerization and heterodimerization in the literature [225], and a number of consequences of GPCR oligomerization such as changes in receptor expression, compartmentalization, recycling, turnover and degradation have been noted mainly in *in vitro* studies [88,304]. Assuming that oligomerization is relatively stable, co-expressed GPCRs may allow graded regulation of a population of functionally equivalent neurons in the PVN and SON, as receptor ratios and the levels of their corresponding ligands vary as a function of the physiological and pharmacological state. This could mean that with oligomerization between GPCRs of the same subclass it is possible that the heterodimer acts as a ‘concentration-dependent switch’ where one GPCR is activated by low agonist concentrations whereas the other is activated by higher agonist concentrations. The signaling of one GPCR could be shut down while the other is active, e.g., by internalization of the ‘inactive’ GPCR (e.g., see co-expressed adenosine A_1/2A_ receptors [55]; note that the four adenosine receptor subtypes are all possibly expressed in the PVN (see Supplementary Table 4)). Moreover, the heterodimer may have new signaling modalities e.g., switching to coupling to a new G protein to activate a new signaling pathway (e.g., see co-expressed dopamine receptors [179]). To add to the complexity, GPCRs may also physically associate with non-GPCRs e.g., the dopamine D5 receptor and the GABA_A_ γ2 ligand-gated ion channel subunit appear to complex leading to an attenuation in D5 receptor-mediated cAMP accumulation and GABA_A_-mediated current [198].

### 6.3 Possible function of orphan GPCRs

Some orphan GPCRs, or indeed some GPCRs with known endogenous ligands, may be constitutively active in the PVN and SON. This is not as far-fetched as it may seem since constitutive activity in GPCRs is a relatively well-known phenomenon that can be signaling pathway-dependent, and can result from the overexpression of receptors in native tissue or heterologous systems, and/or by changes in the DNA (introduced or somatic mutations) or RNA (*visa vi* RNA editing as in the 5-HT_2C_ receptor [213]) sequence of GPCRs. For example, isoforms of the histamine H_3_ receptor are constitutively active pre- and post-synaptically in native brain cortical tissue [230], and in cell lines α_1A_- and α_1B_-adrenoceptors (which can heterodimerize) [60], bradykinin B_2_ [261], ghrelin [129], melanocortin MC_4_ [239] and neurotensin NTS_2_ [129] receptors exhibit constitutive activity, and co-expression of the constitutively active histamine H_1_ receptor with the 5-HT_1B_ receptor confers constitutive activity on the latter receptor [14]. All these GPCRs are expressed in the PVN and SON. The orphan GPCRs GPR3, 6, 12, 20, 26 (present in SON by DNA microarrays), 39, 61 (present in PVN and SON by DNA microarrays) and 78 also alter basal signal transduction activity when expressed *in vitro* [129,148,321,329]. Although we may be able to predict changes in GPCR activity based on altered GPCR sequences, the demonstration of constitutive activity in the PVN and SON needs to be functionally-based. A component of the high, basal [^35^S]GTPγS labeling in the rat PVN [1] may reflect constitutive basal activity of known and/or orphan GPCRs. Highly expressed, constitutively active GPCRs may account in part for the molecular mechanisms regulating signal transduction effectors in PVN/SON neurons. Some of these molecules e.g., cAMP, Ca^2+^, have key roles in axonal growth of developing or regenerating neurons (e.g., [226]). Constitutive activity may also underlie ligand-independent functions of orphan GPCRs such as involvement in GPCR heterodimerization and altering target GPCR function – an example of this is the orphan GPCR GPR50 heterodimerizing with the melatonin MT_1_ receptor to inhibit its activity (see [187]). In fact, constitutive activity is observed in the PVN and SON. For example, nitric oxide whose generation is enhanced by many GPCR agonists, and which has general inhibitory neuroendocrine and autonomic effects in the PVN and SON [306], constitutively restrains ongoing firing in SON neurons [307]. As to GPCR-‘specific’ effects, very recently the melanocortin MC_4_ receptor was shown to be constitutively active in the mouse PVN [96].

There are a number of candidate substances that may be ligands for orphan GPCRs expressed within the PVN and SON. These include peptides that modulate PVN/SON function and/or are perhaps expressed in mPVN and SON, or pPVN neurons. Various peptides derived from larger precursor molecules (and isolated by proteomic methods) are expressed in the PVN/SON, such as: (1) the neuroendocrine regulatory peptides (NERPS-1/2), which are products of the VGF gene that colocalize with VP in the storage granules of the PVN and SON of both rats and humans, and suppress basal, hypertonic saline- or angiotensin II-induced VP release [328,363]; (2) neuronostatin, a product of the somatostatin gene that depolarizes or hyperpolarizes PVN magnocellular, parvocellular or preautonomic neurons and administered centrally increases blood pressure and decreases food intake and water drinking [279]; (3) nesfatin-1, an amino-terminal fragment derived from NEFA/nucleobindin 2 (NUCB2) [93] that is present in VP and OT neurons [30], elevates intracellular Ca^2+^ in dissociated hypothalamic [30] and isolated PVN [211] neurons, alters the electrophysiological properties of PVN neurons [257], increases OT release from PVN tissue slices [211] and administered centrally increases c-*fos* in the PVN/SON and decreases food intake via an OT-dependent, leptin-independent melanocortin pathway [211,93] (note that it has been reported that nesfatin activates GPR12 [229], an orphan GPCR which exhibits constitutive activity [321]); and (4) augurin, a product of the c2orf40 gene which encodes the esophageal cancer-related gene 4 (ECRG4) protein) [318] that is present in PVN and SON OT and VP neurons [271], increases VP and CRF release from hypothalamic explants and elevates plasma ACTH levels when administered centrally or intra-PVN [318]. It is interesting to note that peptidomics of the rat SON has identified 20 unique peptides from known pro-hormones [27]. Candidate orphan GPCR ligands are not restricted to peptides and their by-products and post-translationally modified counterparts, but could also include compounds such as steroids (e.g., glucocorticoids) that are known to interact with the PVN/SON. There are a number of ‘fast’, apparently non-genomic effects of steroids [199] and their metabolites (e.g., see [236]) that may be mediated by GPCRs, including chemosensory receptors, in the PVN/SON and other brain regions. A comparison of brain region and peripheral tissue localization and effects of candidate orphan GPCR ligands with the anatomical distribution of orphan GPCR gene expression could possibly contribute to GPCR ‘deorphanization’ in the PVN and SON.

## 7 Concluding remarks

The neuronal universe of GPCRs and associated signaling components in the PVN and SON is expanding: removal of one constituent may not upset the fabric of the system in its basal state (e.g., there is probably some redundancy in the system) but we can measure alterations in individual elements, and these may influence the function of the system as a whole as it responds to different stimuli. It is evident that GPCR gene expression and protein data, preferably verified by at least two methods, needs to be complemented with functional data. Single cell transcriptomic profiling (including deep sequencing) will likely extend the number of potentially functional GPCRs in PVN and SON neurons, but such studies will possibly have to sample a number of different neuronal populations to deal with the heterogenous nature of the cells. These experiments can be performed on electrophysiologically-identified neurons, or on cells that have been identified by complementary techniques such as high-resolution 2-photon Ca^2+^ imaging that has been extensively used in other brain regions like the hippocampus (e.g., [258]). GPCRs are not abundant compared to most cytoplasmic proteins, and are difficult to analyze by gel-based techniques such as 2D-difference gel electrophoresis (DIGE) due to their inherent hydrophobicity and insolubility in standard detergents. Global proteomic approaches to analyze GPCR expression are still not within reach, currently requiring methods such as selectively tagging (e.g., by biotinylation) cell surface proteins, various chromatography techniques including immobilized-metal affinity chromatography (IMAC), or the isotope-coded affinity tag (ICAT) method prior to protein analysis by mass spectrometry [344].

It is likely in the future that high-resolution RNA-[347] and protein-tracking methods will be used in conjunction with functional imaging to study the consequences of spatial and temporal changes in GPCR expression in the PVN, SON and elsewhere in the brain. Concerning the detection of GPCR proteins themselves, although fluorescent and biotinylated GPCR ligands have been available since the 1970s, it is only fairly recently that improved fluorophores and conjugation methods with increased signal/noise ratios and bioactivity have made these compounds viable alternatives to radiolabeled compounds in viewing the native tissue distributions of GPCRs. For example, while fluorescent GPCR ligands may not have been specifically applied to either PVN or SON sections or viable tissue, they have been used to detect relatively abundant GPCRs such as α-adrenoceptors in arteries and dopamine receptor subtypes in brain [8,64]. We are in the midst of an era where fluorescent GPCR ligands can also potentially be used to investigate GPCR internalization and fluorescence resonance energy transfer (FRET)- and bioluminescence energy transfer (BRET)-based measurements to reveal oligomer formation between homologous or heterologous GPCRs, or between other proteins in single PVN/SON cells or within whole tissue [4].

Undoubtedly valuable physiological information will be obtained by the continued use of murine (and perhaps future rat) GPCR knockout models, even though there are ongoing concerns over compensatory mechanisms possibly distorting phenotypes in global knockouts, potentially different physiological phenotypes (e.g., behavior) on different genetic backgrounds, and the different PVN cytoarchitecture between rats and mice [352]. One mouse line with conditional PVN transcriptional re-activation (i.e., functional recovery) of a GPCR has been reported (melanocortin MC_4_ receptor [15]) but to our knowledge no inducible (e.g., by drugs such as tetracyclin) PVN/SON GPCR knockouts are available. ‘Translational’ research dictates the importance of applying scientific discoveries into practical applications like improving human health. Variations in GPCR agonist levels or in GPCRs themselves, e.g., brought about by exposure to stressors, is potentially relevant to a number of human conditions such as depression and cardiovascular disorders where GPCR changes may promote pathological outcomes. Studies on GPCRs in the human PVN and SON are relatively scarce and often fraught with over-interpretation when compared with rat experiments, not least because the human PVN does not have the clear anatomical demarcations as its rat equivalent [314]. Species differences in brain GPCR distribution are also not uncommon (and may partly underlie some behavioral phenotypes [367]), advising caution in extrapolating data on GPCRs from animals to humans. However, conservation of GPCR expression in the PVN/SON between rodents and humans or non-human primates reinforces the idea that a particular GPCR may have an important functional role. For example, the 5-HT_1A/1C_ [252], β_2_-adrenoceptor [197], dopamine D_1/2/3/5_ [108,270], neuropeptide Y_5_ [238] and tachykinin NK_3_ [168] receptors are all present in the human PVN and SON, while CRF_1_ and VP V_1A_ receptor transcript levels appear to be elevated in the PVN of depressed patients [340]. Other GPCRs like the free fatty acid FFA1 [209] and melatonin MT_1_ [360] receptors are present in the primate PVN and SON by IHC but to date have not been shown in the same structures in the rat. A number of studies in humans are compatible with those in rats suggesting GPCR action in the PVN. For example, peripheral melatonin modulates the VP response to exercise and hypertonic saline infusion [86], while naloxone (a μ-opioid receptor blocker) enhances the plasma ACTH response to CRF [56]. In non-human primates, the odd study has investigated the effects of intranasal [250] and central [296] administration of GPCR ligands on PVN-based activity. Further exploration of GPCR expression and function in the PVN and SON of humans and experimental animals, including correlative studies on the possible impact of GPCR gene variations (e.g., single nucleotide polymorphisms), will contribute significantly to unraveling the influence of GPCRs on the homeostatic roles of the PVN and SON under normal conditions and in disease states.

## Figures and Tables

**Table 1:** Summary of GPCRs expressed in the rat PVN and SON.

GPCRs expressed in the PVN and SON	
Total number of known GPCRs^a^	224
Number of orphan GPCRs^a^	96 (class A), 29 (class B) and 7 (class C) = 132
Known GPCRs in rat PVN	94 + 7 based on functional criteria + 14 unvalidated arrays
Orphan GPCRs in rat PVN	9 by ISHH + 17 unvalidated arrays
Known GPCRs in rat SON	74 + 6 based on functional criteria + 16 unvalidated arrays
Orphan GPCRs in rat SON	9 by ISHH + 21 unvalidated arrays

a Numbers based on lists in the on-line IUPHAR Database of Receptors and Ion Channels (http://www.iuphar-db.org/index.jsp) [111] excluding chemosensory (e.g., olfactory, vomeronasal, taste) receptors and possible spliced (see Supplementary Tables 5 and 6) GPCR variants.

**Table 2:** GPCR families expressed in the PVN.

GPCR families expressed in the PVN	
5-HT	*Melanocortin*
Acetylcholine muscarinic	Metabotropic glutamate
Adenosine	*Neuromedin U*
Adrenoceptor	*Neuropeptide FF*
*Angiotensin*	*Neuropeptide S*
*Apelin*	*Neuropeptide W*
*Bombesin*	*Neuropeptide Y*
*Bradykinin*	*Neurotensin*
*Calcitonin*	*Opioid*
Calcium-sensing	*Orexin*
Cannabinoid	P2Y
*Chemokine*	*Parathyroid hormone*
*Cholecystokinin*	*Peptide P518 (QRFP)*
*Corticotropin-releasing factor*	*Prokineticin*
Dopamine	*Prolactin-releasing peptide*
*Endothelin*	Prostanoid
Estrogen	*Relaxin*
GABA_B_	*Somatostatin*
*Galanin*	*Tachykinin*
*Ghrelin*	*Thyrotropin-releasing hormone*
*Glucagon*	*Urotensin*
Histamine	*VIP & PACAP*
*Melanin-concentrating hormone*	*Vasopressin and oxytocin*

There are 46 GPCR families expressed in the PVN, including 33 different peptide classes (in italics). Notably absent are lipid-like GPCRs (e.g., lysophospholipids) which were detected in DNA microarrays but whose presence in the PVN (or SON) has not been validated, and the anaphylatoxin, formyl peptide, kisspeptin, leukotriene, melatonin, motilin, platelet-activating factor, and trace amine GPCRs for which there are functional responses in the PVN following central or peripheral administration of agonists, or in HNS cultures *in vitro*. The vast majority of GPCRs expressed in the PVN are also present in the SON – the exceptions are members of anaphylatoxin, formyl peptide, leukotriene, platelet-activating factor, and trace amine GPCRs which have not been demonstrated in the SON to our knowledge. To date members of the bile acid, free fatty acid, glycoprotein hormone, gonadotropin-releasing hormone and hydroxyl acid GPCRs families do not appear to be expressed in either the PVN and SON.

## Appendix A Supplementary material

Supplementary Table 1 GPCR signal transduction components detected in the PVN by DNA microarrays.

Supplementary Table 2 GPCR signal transduction components detected in the SON by DNA microarrays.

Supplementary Table 3 GPCRs detected in the rat PVN and SON by receptor ARG.

Supplementary Table 4 GPCRs in the paraventricular nucleus (PVN) and supraoptic nucleus (SON) of the rat hypothalamus: a summary of the literature.

Supplementary Table 5 GPCRs noted as present in rat PVN arrays.

Supplementary Table 6 GPCRs noted as present in rat SON arrays.

Supplementary Table 7 Orphan GPCRs in the rat PVN & SON.

Supplementary Table 8 Examples of GPCR regulation in the PVN and SON.

Supplementary Table 9 Some functions of GPCRs in the rat PVN/SON.

Supplementary references References for Supplementary Tables.
