# Supplementary material for: G protein-coupled receptors in the hypothalamic paraventricular and supraoptic nuclei – serpentine gateways to neuroendocrine homeostasis
Source: Front Neuroendocrinol. 2012 Jan;33(1):45–66. doi: 10.1016/j.yfrne.2011.07.002 (PMC3336209; doi:10.1016/j.yfrne.2011.07.002)
Supplement: Supplementary Table 1 — GPCR signal transduction components detected in the PVN by DNA microarrays. [file mmc1.doc]

| **Expression level** | **Signal transduction components in the PVN** | **Expression level** | **Signal transduction components in the PVN** |
| --- | --- | --- | --- |
| 2806 | Calmodulin 2 | 226.7 | Protein kinase C, zeta |
| 2731 | Calmodulin 1 | 221.8 | Guanine nucleotide binding protein 12 |
| 2265 | Dynamin 1 | 203.6 | Mitogen activated protein kinase 3 (Mapk3) |
| 1745 | Clathrin, heavy polypeptide | 197.5 | Adenylate cyclase 5 |
| 1447 | Guanine nucleotide binding protein, beta 2-like 1 (Gnb2l1) | 188.2 | Adenylate cyclase 6 |
| 1277 | Protein kinase, cAMP dependant regulatory, type 1, alpha | 162.6 | Guanine nucleotide binding protein, alpha z (Gnaz) |
| 1235 | Clathrin, light polypeptide | 161.2 | Mitogen activated protein kinase kinase 5 (Map2k5) |
| 1071 | Calmodulin 3 | 150.1 | Regulator of G protein signaling 19 |
| 1045 | Guanine nucleotide binding protein, beta 1 (Gnb1) | 141 | Dynamin 3 |
| 794 | Mitogen activated protein kinase kinase 1 (Map2k1) | 134.7 | G protein-coupled receptor kinase 6 |
| 794 | Guanine nucleotide binding protein, gamma 10 (Gng10) | 127.5 | Phospholipase C, delta 4 |
| 734.2 | Protein kinase C, beta 1 | 118 | Regulator of G protein signaling 2 |
| 679.1 | Guanine nucleotide binding protein, alpha 12 (Gna12) | 116 | MAP kinase-activated protein kinase 2 (Mapkapk2) |
| 616.4 | Regulator of G protein signaling 4 | 111.9 | Protein kinase, cAMP-dependent, regulatory, type 2, alpha |
| 613.2 | Guanine nucleotide binding protein, beta 5 (Gnb5) | 105.5 | Arrestin, beta 2 |
| 530.4 | Calcium/calmodulin-dependent protein kinase II, delta | 103.3 | Protein kinase C, gamma |
| 494.5 | Calcium/calmodulin-dependent protein kinase II, beta | 102.2 | Guanine nucleotide binding protein, alpha 11 (Gna11) |
| 471.8 | Regulator of G protein signaling 2 | 102.2 | Regulator of G protein signaling 9 |
| 454 | Phospholipase A2, gamma | 90.1 | Phospholipase D2 |
| 430.3 | Adenylate cyclase 2 | 81.02 | Guanine nucleotide binding protein, beta 4 (Gnb4) |
| 406.6 | Guanine nucleotide binding protein, beta 2 (Gnb2) | 79.78 | Regulator of G protein signaling 5 |
| 400.8 | Calcium/calmodulin-dependent protein kinase II, gamma | 77.96 | Mitogen activated protein kinase 12 (Mapk12) |
| 396.7 | Protein kinase C, lambda | 75.9 | Regulator of G protein signaling 12 |
| 387 | Protein kinase C, epsilon | 70.46 | Guanine nucleotide binding protein, gamma 11 (Gng11) |
| 386.1 | Mitogen activated protein kinase kinase 2 (Map2k2) | 67.14 | Phospholipase C, delta 1 |
| 382.4 | Phospholipase C, gamma 1 | 65.22 | Protein kinase C, delta |
| 381.3 | Regulator of G protein signaling 7 | 63.5 | Calcium/calmodulin-dependent serine protein kinase |
| 367.8 | Guanine nucleotide binding protein, alpha o (Gnao) | 62.06 | Phospholipase D1 |
| 367.6 | Phospholipase C, beta 4 | 60.54 | Guanine nucleotide binding protein, gamma 12 (Gng12) |
| 350.1 | Mitogen activated protein kinase kinase kinase 12 (Map3k12) | 50.62 | Guanine nucleotide binding protein, beta 3 (Gnb3) |
| 330.4 | Regulator of G protein signaling 17 | 41.7 | Phospholipase A2, group IVA (cytosolic, calcium-dependent) |
| 307.8 | Mitogen activated protein kinase 10 (Mapk10) | 41.4 | Calcium/calmodulin-dependent protein kinase kinase 2,beta |
| 282.5 | G protein-coupled receptor kinase 5 | 40.6 | Phospholipase A2, group VI |
| 264.3 | Regulator of G protein signaling 10 | 38.24* | * Regulator of G protein signaling 14 |
| 261.7 | Mitogen activated protein kinase 6 (Mapk6) | 34.06 | Regulator of G protein signaling 8 |
| 257.7 | Calcium/calmodulin-dependent protein kinase II, alpha | 32.08 | Protein kinase C, eta |
| 251.6 | Mitogen activated protein kinase 14 (Mapk14) | 27.2 | Protein kinase C, alpha |
| 235.3 | Adenylate cyclase 3 | 23.24 | Phospholipase C, epsilon 1 |
| 230.2 | Mitogen activated protein kinase 1 (Mapk1) | 21.38 | Regulator of G protein signaling 3 |
| Comparative levels (arbitary units) of genes listed as present in the PVN on Affymetrix 230 2.0 rat genome chips as in [115]. The chips contain 31,099 individual probe sets covering about 30,000 transcripts encoded by 28,000 genes. Selected intracellular signaling component transcripts on the DNA microarray were isolated using specific wildcard operator terms such as ‘calmodulin’, ‘protein kinase’, ‘guanine nucleotide’, ‘phospholipase’, ‘G protein’ and ‘adenylate cyclase’. The list is not a comprehensive coverage of all signaling molecule transcripts in the PVN - many kinases and potential splice variants, for example, have not been included. Apart from differences in relative gene expression levels, all genes in the PVN are present in the SON except RGS14 (asterisk). Signaling molecule data sets such as those presented here can be searched in general gene/protein databases such as the National Center for Biotechnology Information (NCBI - <http://www.ncbi.nlm.nih.gov/>) and the European Bioinformatics Institute (<http://www.ebi.ac.uk/>), and/or some specific online databases (e.g., <http://www.afcs.org/>; <http://www.signaling-gateway.org/>; <http://dip.doe-mbi.ucla.edu/dip/Main.cgi>) that provide resources for the analysis of core signaling components and signaling clusters, including the importance of individual components such as Ca^2+^and cAMP. There are also a number of useful Web resources that deal with various aspects of cell signaling listed at <http://www.cellsignal.com/reference/webResources.html>. | | | |
