# Supplementary material for: G protein-coupled receptors in the hypothalamic paraventricular and supraoptic nuclei – serpentine gateways to neuroendocrine homeostasis
Source: Front Neuroendocrinol. 2012 Jan;33(1):45–66. doi: 10.1016/j.yfrne.2011.07.002 (PMC3336209; doi:10.1016/j.yfrne.2011.07.002)
Supplement: Supplementary Table 2 — GPCR signal transduction components detected in the SON by DNA microarrays. [file mmc2.doc]

| **Expression level** | **Signal transduction components in the SON** | **Expression level** | **Signal transduction components in the SON** |
| --- | --- | --- | --- |
| 2652 | Calmodulin 2 | 196.9 | Adenylate cyclase 6 |
| 2545 | Calmodulin 1 | 188.4 | Adenylate cyclase 3 |
| 1879 | Clathrin, heavy polypeptide | 187.6 | Protein kinase C, zeta |
| 1779 | Dynamin 1 | 186.5 | Mitogen activated protein kinase 3 (Mapk3) |
| 1759 | Guanine nucleotide binding protein, beta 2-like 1 (Gnb2l1) | 183.5 | Regulator of G protein signaling 17 |
| 1427 | Protein kinase C, beta 1 | 163.3 | Dynamin 3 |
| 1220 | Clathrin, light polypeptide | 145.4 | MAP kinase-activated protein kinase 2 (Mapkap2) |
| 1164 | Protein kinase, cAMP dependent regulatory, type I, alpha | 138.1 | Protein kinase C, delta |
| 922.6 | Guanine nucleotide binding protein, beta 1 (Gnb1) | 137.7 | Mitogen activated protein kinase kinase 5 (Map2k5) |
| 832.3 | Calmodulin 3 | 132.2 | Phospholipase C, delta 4 |
| 807.9 | Guanine nucleotide binding protein, gamma 10 (Gng10) | 105.1 | Regulator of G protein signaling 2 |
| 771.1 | Calcium/calmodulin-dependent protein kinase II, gamma | 104.1 | G protein- coupled receptor kinase 6 |
| 767.3 | Guanine nucleotide binding protein, alpha 12 (Gna12) | 101.3 | Regulator of G protein signaling 5 |
| 691.6 | Mitogen activated protein kinase kinase 1 (Map2k1) | 100.9 | Guanine nucleotide binding protein, alpha 11 (Gna11) |
| 588.5 | Regulator of G protein signaling 4 | 98.74 | Guanine nucleotide binding protein, alpha z (Gnaz) |
| 523.5 | Guanine nucleotide binding protein, gamma 11 (Gng11) | 97.54 | Regulator of G protein signaling 9 |
| 499.8 | Guanine nucleotide binding protein, beta 5 (Gnb5) | 97.28 | Phospholipase D2 |
| 488.4 | Calcium/calmodulin-dependent protein kinase II, delta | 94.14 | Guanine nucleotide binding protein, beta 4 (Gnb4) |
| 423.6 | Adenylate cyclase 2 | 93.24 | Calcium/calmodulin-dependent protein kinase II, alpha |
| 410.4 | Phospholipase A2, gamma | 91.5 | Phospholipase D1 |
| 386.1 | Phospholipase C, gamma 1 | 83.12 | Arrestin, beta 2 |
| 382.7 | Phospholipase C, beta 4 | 82.4 | Regulator of G protein signaling 12 |
| 380.6 | Protein kinase C, lambda | 82.36 | Guanine nucleotide binding protein, gamma 12 (Gng12) |
| 379.3 | Guanine nucleotide binding protein, beta 2 (Gnb2) | 82.18 | Regulator of G protein signalling 4 |
| 364.9 | Mitogen activated protein kinase kinase 2 (Map2k2) | 79.5 | Phospholipase C, delta 1 |
| 332.9 | Regulator of G protein signaling 2 | 74.12 | Mitogen activated protein kinase 12 (Mapk12) |
| 299.8 | Calcium/calmodulin-dependent protein kinase II, beta | 72.18 | Phospholipase A2, group IVA (cytosolic, calcium-dependent) |
| 293.8 | Mitogen activated protein kinase kinase kinase 12 (Map3k12) | 64.34 | Regulator of G protein signaling 19 |
| 290.5 | Regulator of G protein signaling 7 | 61.42 | Protein kinase C, gamma |
| 281.5 | Guanine nucleotide binding protein, alpha o (Gnao) | 60.08 | Calcium/calmodulin-dependent serine protein kinase |
| 271 | Regulator of G protein signaling 10 | 58.16 | Phospholipase C, delta 4 |
| 267.5 | Protein kinase C, epsilon | 54.46 | Guanine nucleotide binding protein, beta 3 (Gnb3) |
| 245.1 | Mitogen activated protein kinase 10 (Mapk10) | 46.92 | Protein kinase C, eta |
| 238 | Guanine nucleotide binding protein, beta 12 (Gnb12) | 44.9 | Phospholipase A2, group VI |
| 224.3 | G protein-coupled receptor kinase 5 | 40.36 | Calcium/calmodulin-dependent protein kinase kinase 2, beta |
| 209.2 | Mitogen activated protein kinase 1 (Mapk1) | 34.64 | Protein kinase C, alpha |
| 208.4 | Mitogen activated protein kinase 14 (Mapk14) | 30.72* | * Adenylate cyclase 4 |
| 202.8 | Adenylate cyclase 5 | 29.6 | Protein kinase, cAMP-dependent, regulatory, type 2, alpha |
| 199.3 | Mitogen activated protein kinase 6 (Mapk6) | 25.68 | Regulator of G protein signaling 3 |
| Comparative levels (arbitary units) of genes listed as present in the SON on Affymetrix 230 2.0 rat genome chips as in [115]. The intracellular signaling component transcripts were isolated as in Table 1. All gene transcripts in the SON are present in the PVN except Adenylate cyclase 4 (asterisk). | | | |
