# Supplementary material for: G protein-coupled receptors in the hypothalamic paraventricular and supraoptic nuclei – serpentine gateways to neuroendocrine homeostasis
Source: Front Neuroendocrinol. 2012 Jan;33(1):45–66. doi: 10.1016/j.yfrne.2011.07.002 (PMC3336209; doi:10.1016/j.yfrne.2011.07.002)
Supplement: Supplementary Table 3 — GPCRs detected in the rat PVN and SON by receptor ARG. [file mmc3.doc]

| **GPCR Family** | **GPCR receptor** | **Region** | **Reference** |
| --- | --- | --- | --- |
| 5-Hydroxytryptamine | 5-HT_1A_ and 5-HT_2_ (subtype unknown) | PVN | [281,8,181] |
| “ | 5-HT_7_ | PVN* | [101] |
| Acetylcholine muscarinic | subtype unknown | PVN and SON | [153] |
| Adrenoceptor | α_1/2_ (subtype unknown) | pPVN | [56] |
| “ | α_2_ (subtype unknown) | PVN and SON | [133] |
| “ | β_2_ | SON | [164] |
| Angiotensin | AT_1_ | PVN and SON | [279] |
| Apelin | APJ | PVN and SON | G. Pope and A-M. O’Carroll unpublished data |
| Bombesin | BB_2_ | PVN and SON | [163] |
| Calcitonin | CT | PVN | [114] |
| Cannabinoid | CB_1_ | PVN and SON | [111] |
| Cholecystokinin | CCK_1_ and CCK_2_ | PVN and SON** | [116] |
| Corticotropin-releasing factor | subtype unknown | PVN | [63] |
| Endothelin | subtype unknown | PVN and SON | [155] |
| GABA_B_ | GABA_B1_ or subtype unknown | PVN and SON | [24] |
| Galanin | subtype unknown | PVN and SON | [35] |
| Ghrelin | Ghrelin | PVN | [104] |
| Glucagon | GLP-1 | PVN | [94] |
| Histamine | H_1_ | PVN and SON | [184] |
| “ | H_3_ | PVN and SON | [249] |
| Melanocortin | subtype unknown | PVN and SON | [300,183] |
| Neuropeptide FF/AF | NPFF1 | PVN | [96] |
| Neuropeptide Y | Y_1_ or unknown subtype | PVN and SON | [70,194] |
| “ | Y_2_ | PVN and SON | [81] |
| “ | Y_4_ | pPVN | [81] |
| Neurotensin | subtype unknown | PVN | [226] |
| Opioid | κ | PVN and SON | [201] |
| “ | NOP | PVN and SON | [223] |
| Relaxin | RXFP3 | PVN and SON | [296] |
| Somatostatin | subtype unknown | pPVN and SON | [176] |
| Tachykinin | NK_1_ | PVN and SON | [258] |
| “ | NK_3_ | PVN and SON | [258,72] |
| VIP and PACAP | VPAC_1_ | PVN and SON | [311,205] |
| “ | subtype unknown | SON | [208] |
| For receptor ARG, typically 8-30μm sections from frozen adult male rat brain are incubated with [^3^H]- or [^125^I]-labelled GPCR ligands (with/without enzyme inhibitors to reduce protelolytic degradation of radiolabeled peptides), washed in cold buffer, and exposed to X-ray film for weeks-months depending on factors such as GPCR abundance, ligand specific activity and signal-noise ratios. pPVN, parvocellular region of the paraventricular nucleus; * receptor subtype discriminated by presence after displacement with other 5-HT ligands; ** receptors discriminated by displacement with CCK_1_ or CCK_2_ ligands. | | | |
