# Supplementary material for: G protein-coupled receptors in the hypothalamic paraventricular and supraoptic nuclei – serpentine gateways to neuroendocrine homeostasis
Source: Front Neuroendocrinol. 2012 Jan;33(1):45–66. doi: 10.1016/j.yfrne.2011.07.002 (PMC3336209; doi:10.1016/j.yfrne.2011.07.002)
Supplement: Supplementary Table 4 — GPCRs in the paraventricular nucleus (PVN) and supraoptic nucleus (SON) of the rat hypothalamus: a summary of the literature. [file mmc4.doc]

| **GPCR** | **PVN** | | | | **SON** | | | |
| --- | --- | --- | --- | --- | --- | --- | --- | --- |
|  | **IHC** | **ISHH** | **Arrays** | **Reference** | **IHC** | **ISHH** | **Arrays** | **Reference** |
| **5-Hydroxytryptamine** |  |  |  |  |  |  |  |  |
| 5-HT_1A_^iii^ | ✓ | ✓ | - | [339,327] | - | ✓ | - | [327] |
| 5-HT_1B_^iii^ | ✓ | - | - | [198,262] | ✓ | - | - | [198] |
| 5-HT_1D_ | - | - | - |  | - | - | - |  |
| 5-HT_1e_ | - | - | - |  | - | - | - |  |
| 5-HT_1F_ | - | - | - |  | - | - | - |  |
| 5-HT_2A_^i^ | ✓ | - | - | [339,337] | - | - | - |  |
| 5-HT_2B_ | - | - | - |  | - | - | - |  |
| 5-HT_2C_^i^ | - | ✓ | - | [110] | - | ✓ | - | [221] |
| 5-HT_4_ | - | - | - |  | - | - | - |  |
| 5-HT_5a_ | - | - | - |  | - | - | - |  |
| 5-HT_5b_ |  |  |  |  |  |  |  |  |
| 5-HT_6_ | - | - | - |  | - | - | - |  |
| 5-HT_7_^ii^ | - | - | ✓ | [115] | - | - | ✓ | [115] |
| **Acetylcholine (muscarinic)** |  |  |  |  |  |  |  |  |
| M_1_^i^ | - | - | - |  | - | - | - |  |
| M_2_^iii^ | - | - | - |  | - | - | - |  |
| M_3_^i^ | - | - | ✓ | [115] | - | - | - |  |
| M_4_ | - | - | - |  | - | - | - |  |
| M_5_ | - | - | - |  | - | - | - |  |
| **Adenosine** |  |  |  |  |  |  |  |  |
| A_1_^iii^ | ✓ | - | ✓ | [179,115] | ✓ | - | ✓ | [259,115] |
| A_2A_^ii^ | ✓ | - | - | [263] | ✓ | - | - | [263] |
| A_2B_^ii^ | - | - | ✓ | [115] | - | - | ✓ | [115] |
| A_3_^iii^ | - | - | ✓ | [115] | - | - | - |  |
| **Adrenoceptors** |  |  |  |  |  |  |  |  |
| α_1A_-adrenoceptor^i^ | - | ✓ | - | [68] | - | ✓ | - | [68] |
| α_1B_-adrenoceptor^i^ | ✓ | ✓ | ✓ | [321,59,115] | - | - | ✓ | [115] |
| α_1D_-adrenoceptor^i^ | - | ✓ | - | [275] | - | - | - |  |
| α_2A_-adrenoceptor^iii^ | ✓ | - | - | [7] | ✓ | - | - | [7] |
| α_2B_-adrenoceptor | - | - | - |  | - | - | - |  |
| α_2C_-adrenoceptor | - | - | - |  | - | - | - |  |
| β_1_-adrenoceptor | - | - | - |  | - | - | - |  |
| β_2_-adrenoceptor^ii^ | - | - | - |  | - | - | ✓ | [115] |
| β_3_-adrenoceptor | - | - | - |  | - | - | - |  |
| **Anaphylatoxin** |  |  |  |  |  |  |  |  |
| C3a | - | - | - |  | - | - | - |  |
| C5a^iii^ | - | - | - |  | - | - | - |  |
| C5L2 | - | - | - |  | - | - | - |  |
| **Angiotensin** |  |  |  |  |  |  |  |  |
| AT_1A_^i,iii^ | ✓ | ✓ | ✓ | [248,319,279,115] | ✓ | - | - | [248] |
| AT_1B_ | - | - | - |  | - | - | - |  |
| AT_2_^iii^ | ✓ | - | - | [10] | ✓ | - | - | [10] |
| **Apelin** |  |  |  |  |  |  |  |  |
| APJ^iii^ | ✓ | ✓ | - | [216,233] | ✓ | ✓ | - | [302,233] |
| **Bile Acid** |  |  |  |  |  |  |  |  |
| GPBA | - | - | - |  | - | - | - |  |
| **Bombesin** |  |  |  |  |  |  |  |  |
| BB_1_ | - | - | - |  | - | - | - |  |
| BB_2_^i^ | - | ✓ | - | [313] | - | ✓ | - | [313] |
| BB_3_^i^ | ✓ | - | - | [130] | ✓ | - | - | [130] |
| **Bradykinin** |  |  |  |  |  |  |  |  |
| B_1_ | - | - | - |  | - | - | - |  |
| B_2_^i,ii,iii^ | ✓ | - | - | [253] | ✓ | - | - | [253] |
| **Calcitonin** |  |  |  |  |  |  |  |  |
| CT^i^ | ✓ | ✓ | ✓ | [22,280,115] | ✓ | - | ✓ | [22,115] |
| AMY_1_ | - | - | - |  | - | - | - |  |
| AMY_2_ | - | - | - |  | - | - | - |  |
| AMY_3_ | - | - | - |  | - | - | - |  |
| Calcitonin receptor-like | - | - | ✓ | [115] | - | - | ✓ | [115] |
| CGRP | - | - | - |  | - | - | - |  |
| AM_1_ | - | - | - |  | - | - | - |  |
| AM_2_ | - | - | - |  | - | - | - |  |
| **Calcium-sensing** |  |  |  |  |  |  |  |  |
| CaS^i^ | ✓ | ✓ | - | [220,260] | - | ✓ | - | [220,260] |
| GPRC_6_ | - | - | - |  | - | - | - |  |
| **Cannabinoid** |  |  |  |  |  |  |  |  |
| CB_1_^iii^ | ✓ | ✓ | ✓ | [39,209,115] | - | - | ✓ | [115] |
| CB_2_ | - | - | - |  | - | - | - |  |
| **Chemokine** |  |  |  |  |  |  |  |  |
| CCR1 | - | - | - |  | - | - | - |  |
| CCR2^iii^ | ✓ | - | - | [15] | ✓ | - | - | [15] |
| CCR3 | - | - | - |  | - | - | - |  |
| CCR4 | - | - | - |  | - | - | - |  |
| CCR5 | - | - | - |  | - | - | - |  |
| CCR6 | - | - | - |  | - | - | - |  |
| CCR7 | - | - | - |  | - | - | - |  |
| CCR8 | - | - | - |  | - | - | - |  |
| CCR9 | - | - | - |  | - | - | - |  |
| CCR10 | - | - | - |  | - | - | - |  |
| CXCR1 | - | - | - |  | - | - | - |  |
| CXCR2 | - | - | - |  | - | - | - |  |
| CXCR3^iii^ | - | - | ✓ | [115] | - | - | ✓ | [115] |
| CXCR4^iii^ | ✓ | - | ✓ | [36,115] | ✓ | - | ✓ | [36,115] |
| CXCR5 | - | - | - |  | - | - | - |  |
| CXCR6 | - | - | - |  | - | - | - |  |
| CXCR7 | - | - | ✓ |  | - | - | ✓ |  |
| CX_3_CR1^iii^ | ✓ | - | - | S.T. Yao *et al*., unpublished data | - | - | - |  |
| XCR1 | - | - | - |  | - | - | - |  |
| **Cholecystokinin** |  |  |  |  |  |  |  |  |
| CCK_1_^i^ | - | ✓ | ✓ | [117,115] | - | ✓ | - | [116] |
| CCK_2_^i^ | - | ✓ | - | [117,217] | - | ✓ | - | [116,217] |
| **Corticotropin-releasing factor** |  |  |  |  |  |  |  |  |
| CRF_1_^ii^ | ✓ | ✓ | - | [127,310] | - | ✓ | - | [310] |
| CRF_2_^ii^ | - | ✓ | - | [310] | - | ✓ | - | [310] |
| **Dopamine** |  |  |  |  |  |  |  |  |
| D_1_^ii^ | ✓ | ✓ | - | [57,320,83] | - | ✓ | - | [83] |
| D_2_^iii^ | - | ✓ | - | [320] | - | - | - |  |
| D_3_ | - | ✓ | - | [30] | - | - | - |  |
| D_4_^iii^ | ✓ | - | - | [61] | ✓ | - | - | [61] |
| D_5_ | - | - | - |  | ✓ | - | - | [147] |
| **Endothelin** |  |  |  |  |  |  |  |  |
| ET_A_^i^ | ✓ | - | ✓ | [161,115] | ✓ | - | ✓ | [161,115] |
| ET_B_^i,ii,iii^ | - | - | ✓ | [115] | - | - | ✓ | [115] |
| **Estrogen** |  |  |  |  |  |  |  |  |
| GPER^i,ii^ | ✓ | ✓ | - | [31,108] | ✓ | ✓ | - | [31,108] |
| **Formylpeptide** |  |  |  |  |  |  |  |  |
| FPR1 | - | - | - |  | - | - | - |  |
| FPR2/ALX | - | - | - |  | - | - | - |  |
| FPR3 | - | - | - |  | - | - | - |  |
| **Free fatty acid** |  |  |  |  |  |  |  |  |
| FFA1 (GPR40) | - | - | - |  | - | - | - |  |
| FFA2 | - | - | - |  | - | - | - |  |
| FFA3 | - | - | - |  | - | - | - |  |
| **Frizzled** |  |  |  |  |  |  |  |  |
| FZD_1_^i,iii^ | - | - | ✓ | [115] | - | - | ✓ | [115] |
| FZD_2_^iii^ | - | - | ✓ | [115] | - | - | - |  |
| FZD_3_ | - | - | - |  | - | - | - |  |
| FZD_4_ | - | - | - |  | - | - | - |  |
| FZD_5_ | - | - | - |  | - | - | - |  |
| FZD_6_ | - | - | - |  | - | - | - |  |
| FZD_8_ | - | - | - |  | - | - | - |  |
| FZD_9_ | - | - | - |  | - | - | - |  |
| SMO | - | - | - |  | - | - | - |  |
| **GABA_B_** |  |  |  |  |  |  |  |  |
| GABA_B1_ | ✓ | ✓ | ✓ | [203,257,24,115] | ✓ | ✓ | ✓ | [203,257,24,115] |
| GABA_B2_ | ✓ | ✓ | - | [257,71] | ✓ | ✓ | ✓ | [257,71] |
| GABAB^iii^ | - | - | - |  | - | - | - |  |
| **Galanin** |  |  |  |  |  |  |  |  |
| GAL_1_^iii^ | - | ✓ | - | [100] | - | ✓ | - | [100] |
| GAL_2_^i,iii^ | - | ✓ | ✓ | [100,62,115] | - | ✓ | ✓ | [62,115] |
| GAL_3_^iii^ | - | ✓ | - | [218] | - | - | - |  |
| **Ghrelin** |  |  |  |  |  |  |  |  |
| ghrelin^i^ | - | ✓ | - | [345] | - | ✓ | - | [99] |
| **Glucagon** |  |  |  |  |  |  |  |  |
| GHRH | - | - | - |  | - | - | - |  |
| GIP | - | - | - |  | - | - | - |  |
| GLP-1^ii^ | - | ✓ | - | [219] | - | ✓ | - | [219] |
| GLP-2 | - | - | - |  | - | - | - |  |
| glucagon | - | - | - |  | - | - | - |  |
| secretin^ii^ | ✓ | ✓ | - | [47] | ✓ | ✓ | - | [47] |
| **Glycoprotein hormone** |  |  |  |  |  |  |  |  |
| FSH | - | - | - |  | - | - | - |  |
| LH | - | - | - |  | - | - | - |  |
| TSH | - | - | - |  | - | - | - |  |
| **Gonadotrophin-releasing hormone** |  |  |  |  |  |  |  |  |
| GnRH | - | - | - |  | - | - | - |  |
| **Histamine** |  |  |  |  |  |  |  |  |
| H_1_^i^ | - | ✓ | - | [184] | - | ✓ | - | [184] |
| H_2_^i^ | - | - | - |  | - | - | - |  |
| H_3_^iii^ | - | ✓ | ✓ | [249,115] | - | ✓ | - | [249] |
| H_4_ | - | - | - |  | - | - | - |  |
| **Hydroxycarboxylic acid** |  |  |  |  |  |  |  |  |
| HCA_1_ | - | - | - |  | - | - | - |  |
| HCA_2_ | - | - | - |  | - | - | - |  |
| **Kisspeptin** |  |  |  |  |  |  |  |  |
| kisspeptin | - | - | - |  | - | - | - |  |
| **Leukotriene** |  |  |  |  |  |  |  |  |
| BLT_1_ | - | - | - |  | - | - | - |  |
| BLT_2_ | - | - | - |  | - | - | - |  |
| CysLT_1_ | - | - | - |  | - | - | - |  |
| CysLT_2_ | - | - | - |  | - | - | - |  |
| FPR2/ALX | - | - | - |  | - | - | - |  |
| **Lysophospholipid** |  |  |  |  |  |  |  |  |
| LPA_1_^i,iii^ | - | - | ✓ | [115] | - | - | ✓ | [115] |
| LPA_2_ | - | - | - |  | - | - | - |  |
| LPA_3_ | - | - | - |  | - | - | - |  |
| LPA_4_ |  |  |  |  |  |  |  |  |
| LPA_5_ |  |  |  |  |  |  |  |  |
| S_1_P_1_^i,iii^ | - | - | ✓ | [115] | - | - | ✓ | [115] |
| S_1_P_2_ | - | - | - |  | - | - | - |  |
| S_1_P_3_ | - | - | - |  | - | - | - |  |
| S_1_P_4_ | - | - | - |  | - | - | - |  |
| S_1_P_5_ | - | - | - |  | - | - | - |  |
| **Melanin-concentrating hormone** |  |  |  |  |  |  |  |  |
| MCH_1_ ^i,ii,iii^ | ✓ | ✓ | - | [112] | ✓ | ✓ | - | [112] |
| **Melanocortin** |  |  |  |  |  |  |  |  |
| MC_1_ | - | - | - |  | - | - | - |  |
| MC_2_ | - | - | - |  | - | - | - |  |
| MC_3_ | - | - | - |  | - | - | - |  |
| MC_4_^ii^ | - | ✓ | - | [151] | - | ✓ | - | [151] |
| MC_5_ | - | - | - |  | - | - | - |  |
| **Melatonin** |  |  |  |  |  |  |  |  |
| MT_1_^iii^ | - | - | - |  | - | - | - |  |
| MT_2_ | - | - | - |  | - | - | - |  |
| **Metabotropic glutamate** |  |  |  |  |  |  |  |  |
| mGlu_1_^i^ | ✓ | ✓ | ✓ | [154,284,115] | ✓ | ✓ | ✓ | [154,284,6,115] |
| mGlu_2_ | - | - | - |  | - | - | - |  |
| mGlu_3_^iii^ | - | ✓ | ✓ | [235,115] | - | ✓ | ✓ | [6,235,115] |
| mGlu_4_^iii^ | - | - | ✓ | [115] | - | - | ✓ | [115] |
| mGlu_5_^i^ | ✓ | ✓ | - | [74] | - | - | - |  |
| mGlu_6_ | - | - | - |  | - | - | - |  |
| mGlu_7_^iii^ | ✓ | ✓ | ✓ | [149,150,115] | ✓ | ✓ | ✓ | [149,150,115] |
| mGlu_8_^iii^ | - | - | ✓ | [115] | - | - | - |  |
| **Motilin** |  |  |  |  |  |  |  |  |
| motilin^i^ | - | - | - |  | - | - | - |  |
| **Neuromedin U** |  |  |  |  |  |  |  |  |
| NMU1 | - | - | - |  | - | - | - |  |
| NMU2^i^ | - | ✓ | - | [98] | - | ✓ | - | [98] |
| **Neuropeptide FF/Neuropeptide AF** |  |  |  |  |  |  |  |  |
| NPFF1^iii^ | - | ✓ | - | [185] | - | - | - |  |
| NPFF2 | - | - | - |  | - | - | - |  |
| **Neuropeptide S** |  |  |  |  |  |  |  |  |
| NPS^i,ii^ | ✓ | ✓ | - | [175,329] | ✓ | - | - | [329] |
| **Neuropeptide W/neuropeptide B** |  |  |  |  |  |  |  |  |
| NPBW1^iii^ | - | ✓ | - | [171] | - | ✓ | - | [171] |
| **Neuropeptide Y** |  |  |  |  |  |  |  |  |
| Y_1_^iii^ | ✓ | ✓ | - | [307,323,245] | ✓ | ✓ | - | [307,323,245] |
| Y_2_^iii^ | - | ✓ | - | [323] | - | ✓ | - | [323] |
| Y_4_^iii^ | - | ✓ | - | [323] | - | ✓ | - | [245] |
| Y_5_^iii^ | ✓ | ✓ | ✓ | [323,245,81,115] | ✓ | ✓ | ✓ | [245,81,115] |
| **Neurotensin** |  |  |  |  |  |  |  |  |
| NTS_1_^i^ | - | ✓ | - | [3] | ✓ | ✓ | - | [3,75] |
| NTS_2_^i^ | - | - | ✓ | [115] | - | - | ✓ | [115] |
| **Opioid** |  |  |  |  |  |  |  |  |
| δ^iii^ | - | ✓ | - | [200] | - | ✓ | - | [200] |
| κ^iii^ | ✓ | ✓ | ✓ | [11,88,115] | ✓ | ✓ | ✓ | [11,88,115] |
| μ^iii^ | ✓ | ✓ | - | [200,199] | ✓ | ✓ | - | [88,199] |
| NOP^iii^ | - | ✓ | ✓ | [223,115] | - | ✓ | ✓ | [223,115] |
| **Orexin** |  |  |  |  |  |  |  |  |
| OX_1_^i,iii^ | ✓ | - | - | [113] | ✓ | ✓ | - | [113,202] |
| OX_2_^i^ | ✓ | ✓ | ✓ | [113,50,303,115] | ✓ | ✓ | - | [50,202] |
| **P2Y** |  |  |  |  |  |  |  |  |
| P2Y_1_^i^ | ✓ | - | - | [289] | ✓ | - | - | [289] |
| P2Y_2_ | - | - | - |  | - | - | - |  |
| P2Y_4_ | - | - | - |  | - | - | - |  |
| P2Y_6_ | - | - | - |  | - | - | - |  |
| P2Y_12_^iii^ | - | - | - |  | - | - | ✓ | [115] |
| P2Y_13_^iii^ | - | - | ✓ | [115] | - | - | ✓ | [115] |
| P2Y_14_ | - | - | - |  | - | - | - |  |
| **Parathyroid hormone** |  |  |  |  |  |  |  |  |
| PTH1^ii^ | - | ✓ | ✓ | [318,115] | - | ✓ | ✓ | [318,115] |
| PTH2^ii^ | ✓ | ✓ | - | [314] | ✓ | - | - | [314] |
| **Peptide P518** |  |  |  |  |  |  |  |  |
| QRFP1^i,iii^ | - | ✓ | - | [84] | - | - | - |  |
| QRFP2^i^ | - | ✓ | - | [141] | - | - | - |  |
| **Platelet-activating factor** |  |  |  |  |  |  |  |  |
| PAF^i^ | - | - | - |  | - | - | - |  |
| **Prokineticin** |  |  |  |  |  |  |  |  |
| PKR_1_ | - | - | - |  | - | - | - |  |
| PKR_2_^i,iii^ | - | ✓ | - | [45,224] | - | - | - |  |
| **Prolactin-releasing peptide** |  |  |  |  |  |  |  |  |
| PRRP^i^ | - | ✓ | - | [261] | - | - | - |  |
| **Prostanoid** |  |  |  |  |  |  |  |  |
| DP_1_ | - | - | - |  | - | - | - |  |
| DP_2_ | - | - | - |  | - | - | - |  |
| EP_1_^i^ | ✓ | ✓ | - | [212,236] | - | - | - |  |
| EP_2_ | - | - | - |  | - | - | - |  |
| EP_3_^iii^ | ✓ | - | - | [222] | - | - | - |  |
| EP_4_^ii^ | - | ✓ | - | [236,340] | - | ✓ | - | [340] |
| FP | - | - | - |  | - | - | - |  |
| IP_1_ | - | - | - |  | - | - | - |  |
| TP | - | - | - |  | - | - | - |  |
| **Protease-activated** |  |  |  |  |  |  |  |  |
| PAR1^i,iii^ | - | - | ✓ | [115] | - | - | ✓ | [115] |
| PAR2 | - | - | - |  | - | - | - |  |
| PAR3 | - | - | - |  | - | - | - |  |
| PAR4 | - | - | - |  | - | - | - |  |
| **Relaxin family peptide** |  |  |  |  |  |  |  |  |
| RXFP1^ii,iii^ | - | ✓ | - | [195] | - | ✓ | - | [195] |
| RXFP2 | - | - | - |  | - | - | - |  |
| RXFP3^iii^ | - | ✓ | - | [296] | - | ✓ | - | [296] |
| **Somatostatin** |  |  |  |  |  |  |  |  |
| sst_1_^iii^ | ✓ | ✓ | - | [160,21] | - | - | ✓ | [115] |
| sst _2_^iii^ | ✓ | ✓ | ✓ | [160,21,115] | - | - | - |  |
| sst _3_^iii^ | ✓ | - | ✓ | [160,115] | - | - | ✓ | [115] |
| sst _4_^iii^ | ✓ | - | - | [160] | - | - | - |  |
| sst _5_ | - | - | - |  | - | - | - |  |
| **Tachykinin** |  |  |  |  |  |  |  |  |
| NK_1_^i^ | - | ✓ | ✓ | [197,115] | - | - | ✓ | [115] |
| NK_2_ | - | - | - |  | - | - | - |  |
| NK_3_^i^ | ✓ | ✓ | - | [67,102,72] | ✓ | ✓ | ✓ | [67,102,72,115] |
| **Thyrotropin-releasing hormone** |  |  |  |  |  |  |  |  |
| TRH_1_^i^ | - | ✓ | ✓ | [234,115] | - | - | ✓ | [115] |
| TRH_2_^i^ | - | ✓ | - | [234] | - | - | - |  |
| **Trace amine** |  |  |  |  |  |  |  |  |
| TA_1_^ii^ | - | - | - |  | - | - | - |  |
| **Urotensin** |  |  |  |  |  |  |  |  |
| UT^i^ | - | ✓ | - | [129] | - | ✓ | - | [129] |
| **VIP and PACAP** |  |  |  |  |  |  |  |  |
| PAC_1_^ii^ | ✓ | ✓ | - | [136,229] | ✓ | ✓ | - | [136,229] |
| VPAC_1_^ii^ | ✓ | - | - | [136] | ✓ | - | - | [136] |
| VPAC_2_^ii^ | ✓ | ✓ | - | [136,192] | ✓ | ✓ | - | [136,192] |
| **Vasopressin and oxytocin** |  |  |  |  |  |  |  |  |
| V_1A_^i^ | ✓ | ✓ | - | [122,121] | ✓ | ✓ | - | [122,121] |
| V_1B_^i^ | ✓ | ✓ | - | [122,121] | ✓ | ✓ | - | [122,121] |
| V_2_ | - | - | - |  | - | - | - |  |
| OT^i^ | ✓ | ✓ | - | [204,214] | ✓ | ✓ | - | [204,214] |
| Detection of GPCR expression by immunohistochemistry (IHC), *in situ* hybridisation histochemistry (ISHH) and DNA microarrays (arrays). Receptor nomenclature is based on the official family receptor names given in the on-line IUPHAR Database of Receptors and Ion Channels (www.iuphar-db.org/) excluding chemosensory GPCRs (e.g., olfactory, vomeronasal and taste receptors), and only includes receptors known to be in the rat genome. For some GPCR sub-families, e.g., calcitonin, pharmacological responses are dictated by additional proteins (e.g., RAMPS: receptor activity-modifying proteins), whereas in others e.g., GABA_B_, heterodimerization of two GPCR subunits is required for a functional response. In some instances only functional GPCRs have been documented (see Supplementary Table 9) – examples of these include responses to anaphylatoxins (C5a), formyl peptides, kisspetin, leukotrienes, platelet-activating factor and trace amines in the PVN, and responses to melatonin and motilin in both the PVN and SON. For the GPCRs expressed (or where functional responses of known or unknown subtypes are indicated), the major Gα protein involved in signal transduction (as per www.iuphar-db.org/) is provided: i, coupled to the G_q/11_: downstream signal increases intracellular Ca^2+^ and stimulates phosphatidyl inositol phosphorylation (also activates phospholipase C, and sometimes phospholipase A_2_ and D); ii, coupled to G_s_: downstream signal increases cAMP; iii, coupled to G_i/o_: downstream signal decreases cAMP. (✓) indicates the presence of either receptor mRNA or protein in the PVN or SON, and (-) denotes either an absence of mRNA or protein in the PVN or SON, or a localization study for that GPCR has not been undertaken or reported to our knowledge. Known or possible splice variants for the rat GPCRs have not been indicated but for some GPCRs (e.g., metabotropic glutamate mGlu_1_ receptors [228]) splice variants have been detected in the PVN and SON. | | | | | | | | |
