# Supplementary material for: G protein-coupled receptors in the hypothalamic paraventricular and supraoptic nuclei – serpentine gateways to neuroendocrine homeostasis
Source: Front Neuroendocrinol. 2012 Jan;33(1):45–66. doi: 10.1016/j.yfrne.2011.07.002 (PMC3336209; doi:10.1016/j.yfrne.2011.07.002)
Supplement: Supplementary Table 5 — GPCRs noted as present in rat PVN arrays. [file mmc5.doc]

| **Expression level** | **PVN GPCRs** | **Expression Level** | **PVN GPCRs** |
| --- | --- | --- | --- |
| 1,270 | GABA_B1_* (1f)^SON^ | 79.74 | ***GPR83***^SON^ |
| 1,197 | NTS_2_ neurotensin^SON^ | 79.05 | ***GPR123****^SON^ |
| 1,046 | GABA_B1_* (1j)^SON^ | 77.38 | FZD_2_ frizzled |
| 900 | ET_B_ endothelin ^SON^ | 76.42 | CXCR7^SON^ |
| 853 | S_1_P_1_ lysophospholipid^SON^ | 75.98 | NK_1_ tachykinin^SON^ |
| 830.6 | GABA_B2_^SON^ | 71.42 | GABA_B1_* (1a) |
| 703.1 | ***GPR37-like1***^SON^ | 69.52 | OX_2_ orexin |
| 596.2 | ***GPRC5b****^SON^ | 68.82 | CCK_1_ cholecystokinin |
| 504.3 | ***GPRC5b****^SON^ | 62.44 | ***GPR149***^SON^ |
| 428.2 | ***GPR56***^SON^ | 61.88 | ***GPR34***^SON^ |
| 390.9 | CB_1_ cannabinoid^SON^ | 60.14 | M_3_ muscarinic |
| 378.8 | mGlu_1_ metabotropic glutamate^SON^ | 60.08 | SST_2_ somatostatin |
| 375.5 | ***GPR37***^SON^ | 59.86 | 5-HT_7_ serotonin^SON^ |
| 362.8 | ***GPR158***^SON^ | 58.26 | FZD_1_ frizzled^SON^ |
| 355.8 | ***GPR162***^SON^ | 58.06 | AT_1_ angiotensin II |
| 351.3 | PAR1 protease-activated^SON^ | 53.94 | SST_3_ somatostatin^SON^ |
| 286.2 | CT calcitonin^SON^ | 50.62 | GAL_2_ galanin* |
| 228.7 | LPA_1_ lysophospholipid^SON^ | 49.44 | mGlu_4_ metabotropic glutamate^SON^ |
| 190.6 | ***GPR48***^SON^ | 49.38 | Y_5_ neuropeptide Y^SON^ |
| 179.3 | H_3_ histamine | 42.74 | CXCR4 chemokine*^SON^ |
| 156.5 | ***GPR123****^SON^ | 40.7 | ***GPR116****^SON^ |
| 152.3 | ***GPR98***^SON^ | 40.62 | mGlu_8_ metabotropic glutamate* |
| 148.8 | TRH_1_ thyrotropin releasing factor^SON^ | 39.96 | ***P2Y5***^SON^ |
| 136.9 | ***GPR85***^SON^ | 36.94 | ***GPR125*** |
| 129.6 | Calcitonin receptor-like^SON^ | 34.58 | GAL_2_ galanin* |
| 128.8 | κ opioid*^SON^ | 33.78 | ***GPR88***^SON^ |
| 121.7 | mGlu3 metabotropic glutamate^SON^ | 33.08 | ***GPR61***^SON^ |
| 120.6 | ***GPR116****^SON^ | 31.38 | P2Y_13_ purinergic^SON^ |
| 114.2 | ***GPR108***^SON^ | 31.02 | CXCR4 chemokine*^SON^ |
| 113.5 | ***GPR176***^SON^ | 30.7 | PTH_1_ parathyroid hormone*^SON^ |
| 111.8 | ***GPR68***^SON^ | 29.34 | κ opioid*^SON^ |
| 109.1 | NOP nociceptin*^SON^ | 28.36 | ET_A_ endothelin*^SON^ |
| 104.5 | SST_1_ somatostatin^SON^ | 27.42 | ***GPR153***^SON^ |
| 104.3 | ***GPR146***^SON^ | 26.66 | ET_A_ endothelin*^SON^ |
| 102.9 | ***GPR19***^SON^ | 25.66 | mGlu_7_ metabotropic glutamate^SON^ |
| 102.8 | ***GPR107***^SON^ | 23.82 | PTH_1_ parathyroid hormone*^SON^ |
| 85.54 | mGlu_8_ metabotropic glutamate* | 20.36 | NOP nociceptin*^SON^ |
| 85.24 | A_2B_ adenosine | 17.82 | A_3_ adenosine |
| 83.78 | A_1_ adenosine^SON^ | 15.96 | CXCR3 chemokine^SON^ |
| 82.24 | α_1B_ adrenoceptor^SON^ |  |  |
| Comparative levels (arbitary units) of GPCR genes listed as present in the PVN on Affymetrix 230 2.0 rat genome chips as in [115]. Lists of genes that are represented on the Affymetrix array were isolated using the wildcard operator terms ‘receptor’, ‘GPCR’, ‘GPR’ and ‘G protein-coupled receptor’ and hand-finished. The Tas1r2 (taste R, type I) and Vom2r44 vomeronasal 2 receptors were also detected in the PVN (expression levels of 175 and 18.72, respectively). The pharmacological specificity of the family of calcitonin (CT) receptors and calcitonin receptor-like receptors are dictated by additional proteins known as receptor activity-modifying proteins (RAMPs). These are integral parts of the receptor complex and were detected in the PVN (expression levels of 333.2 and 324 for Ramp1 and Ramp2, respectively). GPCRs also found in the SON (superscript) are indicated. GPR107 and GPR108 appear to have a 7TM structure but show little homology to other GPCRs. Orphan GPCRs are in ***bold*** ***italics***. For comparison, OT, VP, β-actin, TRH and CRF were detected at expression levels of 4,090, 3,531, 2801, 614.7 and 282, respectively. * denotes GPCRs with possible spliced transcripts. | | | |
