# Supplementary material for: G protein-coupled receptors in the hypothalamic paraventricular and supraoptic nuclei – serpentine gateways to neuroendocrine homeostasis
Source: Front Neuroendocrinol. 2012 Jan;33(1):45–66. doi: 10.1016/j.yfrne.2011.07.002 (PMC3336209; doi:10.1016/j.yfrne.2011.07.002)
Supplement: Supplementary Table 6 — GPCRs noted as present in rat SON arrays. [file mmc6.doc]

| **Expression level** | **SON GPCRs** | **Expression Level** | **SON GPCRs** |
| --- | --- | --- | --- |
| 1,312 | NTS_2_ neurotensin | 70.88 | A_1_ adenosine |
| 1,096 | ET_B_ endothelin | 70.24 | CXCR4 chemokine* |
| 1,006 | GABA_B1_* (1f) | 68.3 | ***P2Y5*** |
| 857.5 | GABA_B1_* (1j) | 67.94 | α_1B_ adrenoceptor |
| 750.1 | S_1_P_1_ lysophospholipid | 61.3 | ***GPR149*** |
| 734.9 | ***GPR37-like 1*** | 60.96 | FZD_1_ frizzled |
| 574.5 | ***GPRC5b*** | 59.86 | NK_3_ tachykinin |
| 521.3 | LPA_1_ lysophospholipid* | 59.24 | ET_A_ endothelin* |
| 499.2 | GABA_B2_ | 56.22 | ***GPR34*** |
| 351 | mGlu_1_ metabotropic glutamate | 56.1 | SST_1_ somatostatin |
| 337.8 | PAR1 protease-activated | 55.26 | ***GPR116**** |
| 331.4 | ***GPR37*** | 54.58 | SST_3_ somatostatin |
| 308.5 | ***GPR158*** | 53.72 | CXCR4 chemokine* |
| 285.6 | CB_1_ cannabinoid | 51.7 | ***GPR88*** |
| 249.4 | ***GPR56*** | 51.14 | PTH_1_ parathyroid hormone |
| 246.2 | ***GPR162*** | 47.82 | ***GPR83*** |
| 191.3 | ***GPR116**** | 42.92 | ***GPR84*** |
| 168 | ***GPR48*** | 41.26 | ***GPR126*** |
| 165.3 | Calcitonin receptor-like | 40.52 | P2Y_12_ purinergic |
| 159.2 | mGlu_3_ metabotropic glutamate | 37.54 | 5-HT_7_ serotonin |
| 147.9 | ***GPR107*** | 34.72 | ***GPR182*** |
| 145.8 | ***GPR123**** | 33.4 | ***GPR61*** |
| 136.3 | CXCR7 | 30.62 | GABA_B1_* (1g) |
| 129.3 | ***GPR85*** | 30.22 | CXCR4 chemokine* |
| 126 | ***GPR108*** | 29.92 | mGlu_4_ metabotropic glutamate |
| 122.7 | ***GPR123**** | 29.68 | ***GPR116**** |
| 118.4 | ***GPR176*** | 28.82 | ET_A_ endothelin* |
| 117.1 | ***GPR19*** | 27.34 | PTH_1_ parathyroid hormone* |
| 114.7 | ***GPR98*** | 26.64 | P2Y_13_ purinergic |
| 113.8 | ***GPR146*** | 25.76 | κ opioid* |
| 104.3 | A_2B_ adenosine | 21.76 | Y_5_ neuropeptide Y |
| 97.52 | κ opioid* | 20.18 | ***GPR153*** |
| 94.66 | CT calcitonin | 19.78 | mGlu_7_ metabotropic glutamate |
| 94.36 | ***GPR68*** | 19.22 | LPA_1_ lysophospholipid* |
| 89.88 | TRH_1_ thyrotropin releasing factor | 19.22 | ***GPR26*** |
| 74.46 | NK_1_ tachykinin | 18.32 | CXCR3 chemokine |
| 72.92 | NOP nociceptin | 15.88 | β_2_ adrenoceptor |
| Comparative levels (arbitary units) of GPCR genes listed as present in the SON on Affymetrix 230 2.0 rat genome chips as in [115]. The GPCR transcripts were isolated as in Supplementary Table 5. Tas1r2 (taste R, type 1) and olfactory receptor Olr414 were also detected at expression levels of 188.2 and 29.4, respectively. Ramps 1 and 2 were detected at expression levels of 358.7 and 388.5, respectively. GPR107 and GPR108 appear to have a 7TM structure but show little homology to other GPCRs. Orphan GPCRs are in ***bold*** ***italics***. For comparison, VP, OT and β-actin were detected at expression levels of 4,996, 4,588 and 3014, respectively. * denotes GPCRs with possible spliced transcripts. | | | |
