# Supplementary material for: G protein-coupled receptors in the hypothalamic paraventricular and supraoptic nuclei – serpentine gateways to neuroendocrine homeostasis
Source: Front Neuroendocrinol. 2012 Jan;33(1):45–66. doi: 10.1016/j.yfrne.2011.07.002 (PMC3336209; doi:10.1016/j.yfrne.2011.07.002)
Supplement: Supplementary Table 7 — Orphan GPCRs in the rat PVN & SON. [file mmc7.doc]

| Common to PVN and SON | Receptor mRNA by ISHH | Reference |
| --- | --- | --- |
| GPR19 | - | - |
| GPR34 | - | - |
| GPR37 | - | - |
| GPR37-like 1 | - | - |
| GPR48 | - | - |
| GPR56 | ✓^*(PVN/SON)^ | [186] ^(PVN only)^; see figure 1 ^(PVN and SON)^ |
| GPR61 | ✓ ^(PVN/SON)^ | [169] |
| GPR68 | - | - |
| GPR83 | x | [272] |
| GPR85 | ✓ ^(PVN/SON)^ | [210] |
| GPR88 | - | - |
| GPR98 | - | - |
| GPR101**^ψ^** | ✓ ^(PVN/SON)^ | [227] |
| GPR107 | - | - |
| GPR108 | ✓^*(PVN/SON)^ | see figure 1 |
| GPR116 | - | - |
| GPR123 | ✓ ^(PVN/SON)^ | [165] |
| GPR125 | ✓^*(PVN+/-SON)^ | see figure 1 |
| GPR146 | ✓^*(PVN/SON)^ | see figure 1 |
| GPR149 | - | - |
| GPR153 | ✓^*(PVN/SON)^ | see figure 1 |
| GPR158 | - | - |
| GPR162 | - | - |
| GPR176 | - | - |
| GPRC5b | - | - |
| P2Y5 | - | - |
| SON only | - | - |
| GPR26 | - | - |
| GPR84 | - | - |
| GPR126 | - | - |
| GPR182 | - | - |
| There are 30 orphan GPCR genes expressed in the PVN and/or SON with 26 common to each nuclei. Expression was detected by DNA microarray profiling [115] with the exception of GPR101 (**^ψ^**). Orphan GPCR expression has been confirmed by ISHH in this study (*) or previously published work. x = present in periventricular PVN, and not detected in pPVN, mPVN or SON by ISHH. (-) denotes that orphan GPCR mRNA has not been reported present in the PVN/SON as assayed by ISHH to our knowledge. There are some interesting features of a number of these orphans, e.g., GPR84 is proposed to be a free fatty acid receptor in one study [124] and GPR126 appears to be preferentially expressed in glia [156,276]. GPR107 and GPR108 appear to have a 7TM structure but show little homology to other GPCRs. Orphan GPCR mRNA is clearly expressed in the mouse PVN and/or SON in some instances, e.g., GPR88, 98, 116, 123, 162, 165 and 176 - see Allen Brain Atlas; <http://www.brain-map.org/>. GPR101 and GPR165 are not represented by probe sets on the Affymetix 230 2.0 rat genome chip used to derive this list [115]. Potential orphan GPCR splice variants are not included in the table. | | |
