# Supplementary material for: G protein-coupled receptors in the hypothalamic paraventricular and supraoptic nuclei – serpentine gateways to neuroendocrine homeostasis
Source: Front Neuroendocrinol. 2012 Jan;33(1):45–66. doi: 10.1016/j.yfrne.2011.07.002 (PMC3336209; doi:10.1016/j.yfrne.2011.07.002)
Supplement: Supplementary Table 8 — Examples of GPCR regulation in the PVN and SON. [file mmc8.doc]

| GPCR | Experimental Conditions | Change in GPCR expression* | Reference |
| --- | --- | --- | --- |
| 5-HT_1A_ | Daily ip injections of paroxetine, a 5-HT uptake inhibitor (1-14 days). | No effect on 5-HT_1A_ expression (ARG in PVN) | [181] |
| 5-HT_1B_ | Gastric bypass on obese male rats (10 days). | ↑ 39% (ir in PVN) | [262] |
| 5-HT_2A_ | Daily ip injections of (-)-1-(2,5-dimethoxy-4-iodophenyl)-2-aminopropane HCl (DOI; 4 or 7 day treatment). | ↓ ~50% (ARG in PVN); ↑ 167% (4 days) or ↑ 191% (7 days) (Westerns on PVN tissue); no effect on mRNA (q-PCR on PVN mRNA). | [281] |
| α_1_- and α_2_-adrenoceptors | Salt loading (10 days) or adrenalectomy (sacrificed after 14 days). | Salt loading ↓ in α_1_ (43%) and α_2_ (50%), while adrenalectomy ↓ (45 %) α_2_ but has no effect on α_1_ (ARG in PVN) | [56] |
| α_1B_ -adrenoceptor | Adrenalectomy (sacrificed 1, 3, 7 or 14 days), some received sc CORT pellets (sc for 7 days). | ↑ mRNA 7 (173%*) or 14 (193%*) days following adrenalectomy; 10 mg CORT prevented adrenalectomy-induced ↑ in mRNA, and 50 mg further ↓ (42%*) mRNA expression (ISHH in PVN). * compared to sham controls. | [59] |
| Angiotensin AT_1_ | 24 hour isolation stress or sc infusion of candesartan, a non-competitive AT_1_ receptor antagonist, for 14 days. | Isolation stress ↑ (~2-fold) receptor binding, an effect inhibited by candesartan pretreatment (ARG in PVN). | [10] |
| “ | 2 week sc administration of PD123319 a AT_2_  receptor antagonist. | ↑ mRNA (~10%) and protein (40-60%) (ISHH and ARG in PVN). | [196] |
| “ | 4 week sc infusion of Angiotensin II (with or without concomitant 4 week icv infusions of MAPK inhibitors or losartan (AT_1_ receptor antagonist)). | Angiotensin II ↑ (~1.5-2-fold) mRNA and protein (~1.5-2-fold), an effect blocked by losartan, the p44/42 MAPK inhibitor PD-98059, and the JNK inhibitor SP-600125, but not p38 MAPK inhibitor SB-203580 (q-PCR, and Westerns on PVN tissue). | [319] |
| “ | Adrenalectomy, CORT (in drinking water), dexamethasone injection (SC), and various stress paradigms. | ~ 20% ↓ mRNA and protein following adrenalectomy (reversed with CORT or dexamethasone); ~ 20% ↑ mRNA and protein following dexamethasone injection in intact rats; immobilization stress ↑ mRNA (49-66%) and protein (~30%) in intact animals; ip salt injection, water deprivation and salt loading also ↑ mRNA and protein in intact animals; stress ↓ (30%) mRNA in adrenalectomized animals (a stress induced CORT surge is required to ↑ mRNA)(ISHH and ARG in PVN). | [1] |
| “ | Coronary artery ligation-induced heart failure | AT_1_ protein ↑ in PVN (Western blots of dissected PVN) | [142] |
| “ | Pharmacological model of acute Na^+^ depletion (furosemide injections). | Furosemide induces a rapid and long-lasting ↑ (estimated ~3-4-fold) in pPVN AT_1A_ mRNA (ISHH) and angiotensin II binding sites (ARG). | [42] |
| Apelin APJ | Acute/chronic restraint stress and/or adrenalectomy. | Acute/chronic restraint ↑ mRNA (↑ 240% acute, ↑ 95% chronic) in pPVN of intact rats; adrenalectomy ↑ mRNA in pPVN (by 301%); restraint in adrenalectomized rats could not ↑ mRNA above that of adrenalectomized controls in pPVN; stress had no effect on mRNA in mPVN although adrenalectomy ↑ 111% mRNA (ISHH in PVN). | [231] |
| “ | 24-48 hour salt-loading (2% NaCl in drinking water) or dehydration. | Salt-loading and dehydration ↑ APJ mRNA (salt loading ↑ mRNA by 101% in mPVN, 75% in SON; dehydration ↑ mRNA by 874% in mPVN, 455% in SON); salt-loading ↑ colocalization of APJ mRNA in VP mRNA- containing neurons (by ~12%) within SON but not mPVN, dehydration had no effect on colocalization in either mPVN or SON (ISHH). | [232] |
| Cannabinoid CB_1_ | Chronic treatment (15 days) with SR 141716A (ip) a selective CB_1_ receptor antagonist | ↑ 10 % (ARG in PVN) | [39] |
| Chemokine CXCR4 | Long Evans and Brattleboro (VP-deficient) rats | ir-CXCR4 ↓ 60% in the PVN/SON of Brattleboro rats, when compared to Long Evans control rats. | [37] |
| Chemokine CX_3_CR1 | Induced chronic heart failure | ↑ ~34 % in pPVN and ~28% in mPVN (ir in PVN) | S.T. Yao *et al*., unpublished data |
| Cholecystokinin CCK_1_ and CCK_2_ | Salt-loading (various durations) | Large ↑ (~8-10-fold) CCK_2_ mRNA and protein in PVN and SON; smaller ↑ CCK_1_ mRNA (PVN ↑ ~169%; SON ↑ ~355%)/ protein (PVN ↑ 26%; SON ↑ 50%)(ISHH/ARG). | [116,217] |
| Corticotropin-releasing factor CRF_1_ | Immobilization stress, dehydration, salt-loading (2% NaCl in drinking water for 12 days), adrenalectomy. | ↑ (50-76%) mRNA in PVN following acute and chronic immobilization stress; dehydration (24 or 60 hours) and salt-loading ↑ mRNA in PVN and SON; transient ↑ mRNA (4-5-fold) in PVN 18 hours after adrenalectomy in PVN but no ↑ following long-term adrenalectomy (4-6 days)(ISHH). | [190,191] |
| “ | Leptin infusions into third ventricle (5 days) and/or running stress (60 min) | Running stress ↑ mRNA in PVN (↑ ~4-5-fold) and SON (↑ ~4-5-fold), an effect blunted by leptin (ISHH). | [120] |
| “ | 0.1 μg CRF microinjection into the PVN. | ↑ (~3-fold) mRNA (ISHH in PVN). | [157] |
| “ | Restraint stress (60 min) on virgin, pregnant or lactating female rats | CRF_1_ mRNA ↑ in PVN in response to restraint; restraint response is augmented (estimated ~1.2-fold) in lactation when compared with parturition (ISHH). | [58] |
| Galanin GAL_1_ and GAL_2_ | 4 day salt-loading (2% NaCl in drinking water), dehydration and food-deprivation. | Salt-loading and dehydration ↑ GAL_1_ mRNA in mainly mPVN and SON (e.g., SON mRNA: ↑ ~143% in salt-loading, and ↑ ~180% in dehydration)(ISHH)) and ↑ total GAL-binding sites (e.g., SON protein ↑ ~120-130% in salt-loading and water deprivation (ARG); food deprivation ↓ GAL_1_ mRNA (~30-45%)(ISHH) but not protein (ARG) in the SON. GAL_2_ mRNA expression in pPVN or SON did not change following salt-loading, dehydration or food deprivation (ISHH). | [35] |
| Ghrelin | 48 hour fasting. | No effect on receptor protein (ARG in PVN) | [104] |
| GPR101 (orphan) | Female virgin vs pregnant or lactating rats | ↑ mRNA at the end of gestation (↑ ~75%) and during lactation (↑ ~125%) in SON and throughout lactation in pPVN (↑ ~200%) (ISHH). | [227] |
| Metabotropic glutamate mGluR_1_ and mGluR_3_ | Stage 5 amygdala kindling (induction of seizures) | ↑ mGluR1 and mGluR3 mRNA (28-61%), but only ↑ mGluR1 protein (↑ ~30%)(ISHH and ir in SON). | [6] |
| Neuropeptide Y Y_1_ | Salt-loading (2% NaCl in drinking water for 24-72 hours) or dehydration (48 hours) | ~2-fold ↑ number of ir-Y_1_ positive cells protein in SON following salt-loading or dehydration (IHC); ~2-3-fold ↑ in Y_1_ mRNA in SON after salt-loading (ISHH). | [307] |
| Neuropeptide Y_1_ and Y_5_ | Comparison of young (8-10 months), presenescent (27-30 months) vs. senescent (27-33 months) Fischer rats | Y_1_ and Y_5_ mRNA ~2-4-fold higher in young versus older animals; no difference in Y_1_ and Y_5_ mRNA between presenescent and sensescent (q-PCR in PVN), although number of ir-Y_1_-expressing PVN neurons ↑ in presenescent compared with young and senescent rats. | [51] |
| Prostanoid EP_4_ | iv LPS/IL-1β, icv PGE_2_ (500ng), im injection of  turpentine. | ~2-fold ↑ EP_4_ mRNA in pPVN following LPS; IL-1β ↑ EP_4_ mRNA in pPVN (but not SON) ~4-6-fold; PGE_2_ ↑ pPVN EP_4_ mRNA ~2-3-fold; turpentine ↑ EP_4_ mRNA ~2-4-fold in pPVN (ISHH). | [236,340,341] |
| VIP/PACAP PAC_1_ | Salt-loading (2% NaCl in drinking water for 5 days) | ↑ protein (ir in SON) | [91] |
| Vasopressin/Oxytocin V_1A_ | 1 week of reduced or high water intake. | High water intake ↓ (59%), while reduced water intake ↑ (26%) mRNA (ISHH in the SON). Reduced water intake significantly ↑ (35%) protein levels (ir in the SON). | [122] |
| Vasopressin/Oxytocin OT | Female virgin vs pregnant or ovariectomized (with progesterone or estrogen replacement) rats. | OTR mRNA expression ↑ ~1.5 fold in SON (but not PVN) at parturition (ISHH); OTR binding ↑ mid-late gestation in the PVN/SON (0.5-2.5 fold); estrogen (but not progesterone) ↑ binding in SON (but not PVN) ~2-2.5 fold in ovariectomized animals (ARG). | [214,20] |
| * Change in GPCR protein (ARG), immunoreactivity (ir/Western), or mRNA (ISHH/RT-PCR/quantitative (q)-PCR). All studies were performed on male rats unless otherwise stated. icv, intracerebroventricular injection; im, intramuscular; ip, intraperitoneal injection; iv, intravenous injection; mPVN, magnocellular PVN; pPVN, parvoceullar PVN; sc, subcutaneous injection. | | | |
