# Supplementary material for: G protein-coupled receptors in the hypothalamic paraventricular and supraoptic nuclei – serpentine gateways to neuroendocrine homeostasis
Source: Front Neuroendocrinol. 2012 Jan;33(1):45–66. doi: 10.1016/j.yfrne.2011.07.002 (PMC3336209; doi:10.1016/j.yfrne.2011.07.002)
Supplement: Supplementary Table 9 — Some functions of GPCRs in the rat PVN/SON. [file mmc9.doc]

| **GPCR** | **Role of GPCRs in regulating PVN/SON activity** | **References** |
| --- | --- | --- |
| **5-Hydroxytryptamine** | 5-HT_1A_ inhibits water intake; ↓ GABA synaptic transmission in presympathetic PVN neurons; and ↑ plasma OT and ACTH levels. The 5-HT_2A/2C_ receptor agonist, DOI ((±)1-(2,5-dimethoxy-4-iodophenyl)-2 aminopropane), ↑ c-*fos* in PVN neurons and plasma OT, prolactin, ACTH and CORT levels. Administration (sc) of 5-HT_1A_ agonist ↑ expression of phosphorylated extracellular signal-regulated kinases (ERKs) in the PVN. | [64,170,242,172,337,54] |
| **Acetylcholine** | Intra-PVN injection of the muscarinic receptor antagonist, or nicotinic receptor antagonist, blocks saline-stimulated ↑ in plasma VP levels. Icv administration of carbachol (muscarinic and nicotinic receptor agonist) stimulates VP release. Muscarine (muscarinic receptor agonist) ↑ the frequency of phasic bursts by VP neurons in the SON *in vitro*. Iv muscarinic agonist arecoline stimulates ACTH and CORT release *in vivo*, and arecoline ↑ CRF secretion in hypothalamic cells *in vitro* (blocked by atropine). Cholinergic stimulation of PVN ↓ body temperature and ↑ water intake, whereas cholinergic stimulation of SON ↑ body temperature (blocked by muscarinic antagonist). | [254,125,89,38,297] |
| **Adenosine** | Application of the selective adenosine A_2A_ receptor agonist depolarizes SON neurons. Adenosine inhibits SON neurons via presynaptic A_1_ receptors. Activation of A_1_ ↓ firing activity and hyperpolarizes PVN presympathetic neurons. | [251,238,179] |
| **Adrenoceptors** | α_2_-adrenoceptor agonist (ip) ↑ c-*fos* expression in OT but not VP neurons. α_1_-adrenoceptor agonist (icv) ↑ CRF mRNA in PVN and plasma ACTH release. Noradrenaline excites both phasic and non-phasic firing SON neurons (putative VP and OT cells, respectively). Noradrenaline microinjected into the PVN ↑ pain threshold via local α-adrenoceptors and NMDA receptors. Noradrenaline ↑ frequency of spontaneous glutamatergic excitatory postsynaptic currents (EPSCs) in PVN and SON magnocellular neurons via presynaptic α_1_-adrenoceptors > α_2_-adrenoceptors. Hypoxia ↑ noradrenaline and CRF release in the PVN and median eminence: Hypoxia-stimulated release of CRF from median eminence is blocked by α_1_-adrenoceptor antagonist but facilitated by α_2_-adrenoceptor antagonist. α_1_-adrenoceptor agonist ↑ the firing rate of spinally projecting PVN neurons (but not in the presence of GABA_A_ or ionotropic glutamate receptor antagonists), and also ↑ the frequency of spontaneous EPSCs and ↓ the frequency of spontaneous inhibitory postsynaptic currents (IPSCs). Icv noradrenaline augments TRH release from the PVN and median eminence in response to acute hypoxia, via α_2_-adrenoceptors. α_2_-adrenoceptor agonist ↓ the amplitude of evoked GABAergic IPSCs and ↓ the frequency of GABAergic miniature IPSCs (blocked by α_2_-adrenoceptor antagonist) in spinally-projecting PVN neurons. VP release from HNS explants ↑ following exposure to a α_1_-adrenoceptor agonist, while simultaneous exposure to ATP and α_1_-adrenoceptor agonist induces a synergistic stimulation of sustained VP and OT release, through activation of multiple α_1_-subtypes (_α1A,B,D_). Stimulations of α_1_-adrenoceptors ↑ intracellular Ca^2+^ in isolated SON explants. Stimulation of α1/α2 receptors modulate spontaneous IPSCs in mPVN and pPVN neurons: α_2A_ ↓ spontaneous IPSCs whereas α_1_ (subtype unknown) ↑ spontaneous IPSCs. | [250,152,332,342,28,43,44,118,178,143,290,291,46] |
| **Anaphylatoxin** | Ip administration of a C5a antagonist ↓ LPS-induced c-*fos* in CRF neurons. | [53] |
| **Angiotensin** | Angiotensin II (ATII) activates the intracellular MAPK and JNK signalling pathways in PVN neurons via AT_1_. ATII stimulates VP, OT, ACTH secretion (some effects may be indirect via SFO). Activation of central AT_1_ receptors is necessary to mount a full HPA axis response to isolation stress: subcutaneous infusion of an AT_1_ antagonist inhibits isolated stress mediated ↑ in pituitary ACTH and adrenal CORT content, an effective glucocorticoid feedback inhibition (a ↓ in PVN CRF mRNA and protein), and an ↑ in AT_1_ binding (ARG) in pPVN. AII excites SON OT and VP neurons in *ex vivo* preparations. AII microinjected into the PVN ↑ blood pressure, which is blocked by systemic administration of an AT1 receptor antagonist. AII depolarizes mPVN neurons and ↑ frequency of EPSCs/excitatory postsynaptic potentials via glutamate release. AII depolarizes and ↑ frequency of action potential discharge of PVN neurons that innervate in the rostral ventrolateral medulla via AT_1_. AII intra-PVN ↑ renal sympathetic nerve activity responses to electrical stimulation: blocked by intra-PVN AT_1_ antagonist. AT_1_ antisense oligonucleotides into PVN ↓ resting renal sympathetic nerve activity and normalizes enhanced cardiac sympathetic afferent reflex in males rats with chronic heart failure. AII ↑ intracellular Ca^2+^ in SON neurons. | [319,80,10,237,13,168,40,344,343,283] |
| **Apelin** | Apelin depolarizes and ↑ axonal action potential firing in VP (but not OT) SON neurons, while ↓ somatodendritic VP release. APJ KO mouse studies suggest apelin stimulates HPA axis activity via both CRF_1_ and VP V_1B_ receptors. | [302,225] |
| **Bombesin** | Icv (but not iv) gastrin-releasing peptide (BB_2_ agonist) ↑ 5-HT in PVN and plasma ACTH and CORT levels. Gastrin-releasing peptide also ↑ CRF release from isolated hypothalami. | [86,85] |
| **Bradykinin** | Bradykinin regulates release of noradrenaline from rat hypothalamic slices. | [305] |
| **Calcitonin** | Icv adrenomedullin-5 ↑ c-*fos* mRNA in PVN & SON OT neurons and ↑ plasma OT levels through central CGRP or AM receptors. Adrenomedullin excites OT but not VP neurons in SON. icv adrenomedullin 2 and adrenomedullin ↑ plasma OT levels (adrenomedullin 2 > adrenomedullin). | [243,306,105] |
| **Cannabinoid** | Endocannabinoids mediate fast-feedback action of glucocorticoids on PVN CRF release; presynaptic action mediates α-MSH-induced inhibition of OT cells. Endocannabinoids released from SON magnocellular soma/dendrites ↓ glutamate release and postsynaptic spiking. | [66,271,65] |
| **Chemokine** | Chemokine stromal cell derived factor 1 SDF-1/CXCL12 (activates CXCR4) icv blocks plasma VP release induced by icv angiotensin or ip injection of NaCl, but has no effect on basal plasma VP levels. Electrophysiological recordings demonstrate that endogenous SDF-1 has no effect on basal plasma VP levels (basal activity not inhibited by a CXCR4 antagonist), though administration of SDF-1 can blunt the autoregulation of VP neurons. | [36] |
| **Cholecystokinin** | CCK ↑ OT neuronal activity and OT release, and ↓ VP neuronal activity and VP release. | [106,269] |
| **Corticotropin-releasing factor** | Stress induces CRF_1_ expression in pPVN and SON. Urocortin 1 (binds CRF_2_ with higher affinity than CRF_1_) injected into the SON alters behaviour in open-field test (blocked by CRF_2_ antagonists). CRF injection into PVN ↑ CRF_1_ mRNA expression in pPVN (blocked by CRF antagonist). CRF ↑ CRF_1_ mRNA levels in primary hypothalamic neuron cultures. CRF icv ↑ plasma OT, with no effect on plasma VP levels. Urocortin 2 and 3 (CRF_2_ agonists) into lateral ventricle ↑ CRF and VP hnRNA in pPVN and plasma CORT levels; effect attenuated by CRF_2_ antagonist. Urocortin 3 into PVN ↑ blood pressure, heart rate and renal sympathetic nerve activity (blocked by CRF_2_ antagonist). CRF (icv) ↑ c-*fos* and CRF mRNA in the pPVN, an effect blocked by a CRF_1_ antagonist. CRF_1_ antagonist intra-PVN potentiates the feeding induced by intra-PVN NPY (CRF systems in the PVN exert inhibitory control over NPY-induced food intake). | [126,76,157,34,206,180,246,109] |
| **Dopamine** | Dopamine acts mainly on D_2_ and D_4_ to ↑ OT-stimulated penile erection. Dopamine ↑ magnocellular neuron excitability: activation of D_4_ ↓ the frequency of miniature IPSCs; D_4_ activation reduces GABA release in SON - dopamine facilitation of neurohypophysial hormone release partly results from distribution of magnocellular neurons; presynaptic D_4_ activation inhibits glutamate neurotransmission onto SON magnocellular neurons (N.B. when deviating from base line activity the modification of both glutamatergic and GABAergic input is essential to allow for phasic or spontaneous firing of VP or OT neurons, respectively). Dopamine D_2_ activation ↑ TRH release. D_4_ receptor activation ↑ ERK phosphorylation and c-*fos* expression in the PVN. | [293,14,12,252,177,25] |
| **Endothelin** | ET_A_ agonists ↑, while ET_B_ agonists ↓ basal VP release. | [264] |
| **Estrogen** | Estrogen ↓ 5-HT-stimulated ACTH release via GPER, independent of estrogen receptor β. Estrogen amplifies the naloxone-induced ↑ in OT neuron firing rate in morphine-dependent female rats. | [330,266,32] |
| **Formylpeptide** | Formylpeptide works concomitantly with annexin to ↓ VP and CRF release. | [135] |
| **GABA_B_** | Activation of GABA_B_ ↓ the frequency of spontaneous and miniature IPSCs in pPVN. | [187] |
| **Galanin** | Galanin ↓ evoked EPSCs and ↓ eEPSC frequency in VP/OT neurons. Administration of galanin (icv) ↓ hypothalamic and neurohypophysial OT, but has no effect on plasma OT or VP levels in euhydrated rats; icv galanin in salt-loaded rats ↑ VP and OT levels within the hypothalamus and neural lobe and ↓ plasma VP and OT levels. Intra-PVN microinjection of galanin ↑ food intake. | [159,49,162] |
| **Ghrelin** | Ghrelin injected into PVN ↑ food intake (inhibited by MC_4_ agonists). Icv and iv administration of ghrelin ↑ VP plasma levels. Knockdown of Ghsr1a in the PVN ↓ body weight and plasma ghrelin levels. Ghrelin icv ↑ c-*fos* in magnocellular and parvocellular PVN OT neurons. Ghrelin (via Ghsr1a) potentiates miniature EPSCs in approximately 80% of SON magnocellular neurons tested. Ghrelin (100nM) ↑ CRF and VP mRNA levels 2-3 fold (maximum response at 6 hours) in hypothalamic 4B cell line. | [285,128,286,239,335,139] |
| **Glucagon** | Secretin ↑ VP release from hypothalamus. Icv glucagon-like peptide-1-(7–36)amide (GLP-1) ↑ VP (though not OT) and CORT release, and ↑ c-*fos* expression mainly in CRF neurons of the pPVN and OT neurons of the mPVN/SON. | [47,167] |
| **Histamine** | Histamine ↑ PVN and plasma OT levels and modulates OT neuronal activity in the SON via H_2_ receptors. In HNS explants histamine depolarizes VP neurons via H_1_ receptors. Histamine H_2_ receptor activation ↑ TRH release from hypothalamic slices. Histamine H_1_ and H_2_ antagonists prevents suckling-induced OT release in PVN. | [18,107,287,41,19] |
| **Kisspeptin** | Kisspeptin injected into PVN augments plasma LH and testosterone levels. | [247] |
| **Leukotriene** | Leukotriene B4 (LTB_4_) ↑ CRF release from hypothalamic explants in vitro. Icv LTB_4_ (via BLT_1_) ↑ PVN CRF mRNA/protein and plasma ACTH/CORT levels in ovalbumin-sensitized animals. | [23,338] |
| **Melanin-concentrating hormone** | MCH icv or directly into the PVN ↑ ACTH release; MCH ↑ CRF release from hypothalamic explants; intra-PVN injection of MCH ↑ food intake. MCH ↓ basal ACTH release at end of lights-on period. | [146,265,26] |
| **Melanocortin** | Icv melanocortin agonist ↑ CRF transcription in PVN and plasma CORT levels. alpha-melanocyte-stimulating hormone (α-MSH) ↑ OT release from SON dendrites and ↓ OT release from neurohypophysial terminals (via MC4). α-MSH ↑ intracellular Ca^2+^ in isolated SON neurons. Adrenoviral shRNA-MC_4_ into PVN ↓ food intake and body mass. α-MSH infused over SON *in vivo*, ↑ c-*fos* mRNA expression, though has no effect on VP hnRNA or mRNA. MC_4_ agonist into PVN ↓ food intake. Melanocortin MC_4_ activation ↑ TRH release from hypothalamic slices. | [188,270,87,145,92,148] |
| **Melatonin** | Melatonin significantly ↓ VP secretion in HNS explants; ↓ substance P-stimulated OT and VP release *in vitro;* and ↓ VP and OT (but not CRF) release from the hypothalamus *in vitro*. | [137,138,333] |
| **Metabotropic glutamate** | Activation of presynaptic group III mGluRs (includes mGlu _4_,mGlu_6_, mGlu_7_,and mGlu_8_ although distribution studies suggest that only mGlu_4_and mGlu _7_ may be present in the SON) ↓ frequency of miniature EPSCs in SON neurons, and ↓ glutamate/GABA release onto SON neurons. Activation of group I mGluRs (mGlu_1_ and mGlu_5_: however only mGlu1 has been shown to be in the SON) ↑ glutamate/GABA release onto SON neurons. Group 1 mGlu agonist injected into PVN ↑ lumbar sympathetic nerve activity (blocked by mGlu_1/5_ antagonists). | [29,277,182] |
| **Motilin** | Icv motilin ↑ c-*fos* in PVN and SON. | [328] |
| **Neuromedin U** | 100nM neuromedin U (NMU) binding of NMU2 depolarizes 31% of pPVN neurons, but not magnocellular neurons, via enhancement of hyperpolarization-activated inward current; Neuromedin S (icv), an endogenous ligand for NMU2, ↑ c-*fos* in OT neurons in PVN and SON, and OT release. Intra-PVN NMU ↓ food intake and ↑ plasma ACTH and CORT levels. NMU ↑ CRF and VP release from hypothalamic explants. | [255,256,273,326] |
| **Neuropeptide FF/neuropeptide AF** | Neuropeptide FF (NPFF) facilitates inhibitory input to mPVN via GABAergic interneurons. Hypervolemia or hyperosmolality-induced ↑ in plasma levels of VP are blunted by NPFF (injected into the lateral ventricle), and hyperosmolality-induced plasma VP is significantly augmented by icv NPFF antibodies. Icv NPFF ↑ c-*fos* expression mainly in brainstem-projecting pPVN OT neurons, and a few VP and CRF neurons. | [132,9,334,134] |
| **Neuropeptide S** | Icv or intra-PVN neuropeptide S ↓ palatable food intake. Intra-PVN injection of neuropeptide S ↑ plasma ACTH and CORT levels, and neuropeptide S ↑ CRF and VP release from HNS explants. | [77,288] |
| **Neuropeptide W/neuropeptide B** | Neuropeptide B (icv) induced increase in ACTH is completely blocked by anti-CRF antibodies with no effect on plasma VP/OT levels. Neuropeptide W (icv) stimulated-rise in plasma CORT is prevented by CRF antagonist without effect on plasma VP/OT levels. Neuropeptide W depolarizes and ↑ spike frequency of PVN neurons. | [274,301] |
| **Neuropeptide Y** | Stimulation of HNS explants with NPY potentiates the VP and OT response to phenylephrine, but has no effect on basal VP and OT release; however the Y1-agonist, [Leu31,Pro34]-NPY stimulates OT and VP under resting conditions, with no effect on phenylephrine stimulated release, suggesting alternative roles for the neuropeptide receptors . *In vivo* intra-PVN injections of NPY ↑ feeding, and plasma VP and CORT levels in males. Intra- SON administration of NPY ↑ plasma OT levels in lactating females via Y_1_ and also ↑ the OT secretory response to α_1_-adrenergic receptor stimulation. Icv NPY ↓ PVN pro-TRH mRNA expression. NPY-induced c-*fos* in mPVN blocked by Y_1_ and Y_5_ antagonists; both Y_1_ and Y_5_ antagonists required to block feeding. Y1 and Y5 agonists delivered in the cerebrospinal fluid ↓ PVN pro-TRH mRNA expression. NPY ↑ CRF release from hypothalamic explants, and ↑ CRF mRNA in the PVN. | [144,174,322,244,78,140,79,304,294] |
| **Neurotensin** | Neurotensin (icv) ↑ plasma ACTH and CORT levels (attenuated by CRF antagonist); icv neurotensin ↑ VP but not CRF in median eminence. | [267] |
| **Opioid** | Opioids have complex (and contradictory) effects on ACTH and CORT release: acute dose of morphine (ip) ↑ plasma ACTH and CORT levels; chronic morphine administration (ip) ↓ plasma ACTH and CORT levels; and endomorphin 1 and 2 have no effect on the CORT levels. Sc injection of κ or μ agonists (but not δ) strongly inhibit the release of OT and VP, however only μ agonists ↓ release when given icv. Icv nociceptin ↑ plasma ACTH/CORT levels and ↑ pPVN CRF mRNA (no effect on VP mRNA), an effect blocked by a nociceptin antagonist. Microinjection of δ agonist into the PVN ↑ ethanol intake, while κ agonist ↓ alcohol intake. μ-agonist DAMGO ([d-Ala^2^,N-Me-Phe^4^,Gly^5^-ol]-enkephalin) ↓ K^+^-induced OT > VP release. Intra-PVN injection of dynorphin ↑ food intake via κ opioid receptor. | [312,52,308,173,16,241,95] |
| **Orexin** | Icv orexin-A ↑ pPVN CRF and VP mRNA levels and plasma ACTH and CORT levels. Orexin A ↑ CRF and NPY release, but not VP from isolated hypothalamic explants, an effect blocked by a neuropeptide Y1 antagonist. Orexin-A depolarizes mPVN and pPVN neurons. | [268,82,2] |
| **P2Y** | ATP ↑ Ca^2+^ in SON neurons. P2Y_1_ activation ↑ Ca^2+^ in HNS SON explants (response blocked by P2Y_1_-selective antagonist); Responses to UTP (most potent at P2Y_2_/Y_4_ receptors) and UDP (most potent agonist for P2Y_6_) occurred in fewer SON neurons and produced smaller peak amplitude when compared to a P2Y_1_ stimulated response. | [73,292] |
| **Platelet-activating factor** | Platelet-activating factor ↑ CRF release from hypothalamic explants. | [69] |
| **Parathyroid hormone** | Icv tuberoinfundibular peptide of 39 residues (TIP39) (the endogenous PTH2 ligand) ↓ VP release in response to dehydration. TIP39 ↑ the release of CRF and VP from *in vitro* hypothalamic explants. Icv TIP39 ↑ basal ACTH release. | [295,316] |
| **Prokineticin** | Prokineticin 2 (PK2) depolarizes the majority of magnocellular neurons, an effect blocked by kynurenic acid (glutamate antagonist), which suggests involvement of glutamate interneurons. On the other hand, PK2 depolarizes the pre-autonomic and neuroendocrine parvocellular neurons through MAPK signalling. | [336] |
| **Prolactin-releasing peptide (GPR10)** | Icv prolactin-releasing peptide ↑ plasma ACTH levels (inhibited by CRF antagonist), ↑ c-*fos* expression in neurons of the pPVN and mPVN, and ↑ plasma OT and VP levels in female rats, but only plasma OT levels in male rats. | [211,331,207] |
| **Prostanoid** | Prostaglandin E_2_ (PGE_2_) ↑ the firing rate of rat SON neurons an effect mimicked by an EP_4_ agonist (but not by EP_1_, EP_2_, or EP_3_ agonists). PGE2 and EP_3_ ↓ the frequency of spontaneous IPSCs in the rat SON (EP_1_, EP_2_, EP_2_ or FP agonists had no significant effects on IPSCs). PGE_2_ effects on phasic and non-phasic neurons in SON mostly excitatory. EP_4_ levels in the PVN are ↑ following iv IL-1β. Icv PGE_2_ ↑ plasma CORT levels. | [123,282,341,278] |
| **Relaxin** | Icv relaxin ↑ c-*fos* expression in VP and OT neurons of PVN and SON, as well as many pPVN neurons. Relaxin-3 ↑ food intake (via RXFP3) when administered intra-PVN or -SON, and water intake when administered icv (via RXFP1?). Relaxin-3 intra-PVN ↑ plasma luteinizing hormone levels. | [213,299] |
| **Somatostatin** | Icv somatostatin (SST) ↑ plasma OT levels in virgin and pregnant rats. Icv SST ↑ OT and VP neuron firing rates and c-*fos* expression in SON and PVN (which may be mediated in part by SST_2_). Retrodialysis of SST onto ventrally exposed SON ↑ VP neuron firing rate but ↓ OT neuron firing rate - this may indicate that the direct effect of SST on SON OT neurons is inhibitory, and may not contribute to enhanced OT release. | [215,93] |
| **Tachykinin** | Hydralazine induced hypotension activates NK_3_ signaling in magnocellular neurons to release VP and OT. Icv NK_1_ antagonist attenuates the duration of plasma ACTH and CORT response to acute restraint stress. Icv NK_1_ antagonists ↓ the activation of c-*fos* in the PVN, and the cardiovascular (↑ in mean arterial pressure and heart rate) and behavioural reactions (hind limb grooming/biting) in response to noxious stimuli (sc formalin). NK_3_ agonist intra-PVN causes hypertension which is blocked by a VP V_1_ antagonist. NK_3_ agonist ↑ VP release and substance P ↑ VP and OT release from HNS explants. | [103,131,55,17,298,119,144] |
| **Thyrotropin-releasing hormone** | Iv TRH appears to ↑ hypothalamic VP content in young male rats, and ↓ hypothalamic OT content in mature male rats. | [48] |
| **Trace amine** | Chronic 10 day delivery of β-phenylethylamine (ip) ↑ CRF mRNA in the PVN, CRF immunoreactivity in the median eminence, and plasma ACTH and CORT levels in response to stress. | [158] |
| **Urotensin** | Icv urotensin II ↑ c-*fos* immunoreactivity in the PVN, heart rate, and plasma glucose and CORT levels. | [317] |
| **VIP and PACAP** | Pituitary adenylate cyclise activating polypeptide (PACAP) excites and ↑ firing rate of both phasic and non-phasic neurons; PACAP stimulates local somatodendritic VP release in SON and ↓ basal glutamate and aspartate release. Icv PACAP ↑ c-*fos* in rat PVN and SON. PACAP ↑ VP/OT release from isolated neural lobe. Intra-PVN injection of vasoactive intestinal peptide (VIP) ↑ plasma ACTH and CORT levels, an effect attenuated by pre-treatment with the CRF_1_ and/or V_1_ receptor antagonists. Peptide histidine isoleucine (PHI) via VIP/PACAP receptors ↑ % c-*fos* staining neurons in PVN. PHI icv or intra-PVN ↓ food consumption in overnight food-deprived rats. PACAP ↑ intracellular Ca^2+^ in isolated SON neurons. | [283,91,230,193,4,5,240,60] |
| **Vasopressin and oxytocin** | VP effects depend on activity of VP neurons; VP neurons with weak phasic or irregular activity are excited by VP, whereas VP neurons with robust phasic activity or continuous activity are inhibited by VP (N.B. most VP neurons do not display phasic activity under basal conditions). OT excites OT and VP neurons but only OT neurons show spike frequency reduction. Ionotropic glutamate receptor antagonists ↓ the effects of OT on firing rate (consistent with presynaptic loci of OT action). V_1A_ antagonists (concomitantly with CRF antagonists) delivered into the PVN ↑ plasma ACTH levels - thus PVN V_1A_ has an inhibitory effect of on VP-induced HPA activity. V_1A_/V_2_ antagonists ↓ osmotically-stimulated intra-SON VP release. OT and VP stimulate the release of OT and VP, respectively from SON *in vitro*. Infusion of lysine VP intra-SON ↑ basal VP release into extracellular fluid. Intra-PVN OT administration ↓ anxiety-related behaviour in male rats, and ↑ ERK phosphorylation in PVN and SON neurons (some expressing VP) which may be linked to the anxiolytic actions of OT. OT and V_1A_ ↑ intracellular Ca^2+^ in isolated SON neurons. OT intra-PVN exerts anxiolytic effects via OT receptor. | [33,315,324,90,189,325,27,166,97,309] |
| Abbreviations: ACTH, adrenocorticotropic hormone; CORT, corticosterone; CRF, corticotropin-releasing factor; EPSCs, excitatory postsynaptic currents; HPA, hypothalamic-pituitary-adrenal; HNS, hypothalamo-neurohypophysial system; IPSCs, inhibitory postsynaptic currents; icv, intracerebroventricular injection; ip, intraperitoneal injection; iv, intravenous injection; pPVN, parvocellular region of the paraventricular nucleus; mPVN, magnocellular region of the paraventricular nucleus; OT, oxytocin; sc, subcutaneous injection; VP, vasopressin; TRH, thyrotropin-releasing hormone. | | |
