# Supplementary material for: G protein-coupled receptors in the hypothalamic paraventricular and supraoptic nuclei – serpentine gateways to neuroendocrine homeostasis
Source: Front Neuroendocrinol. 2012 Jan;33(1):45–66. doi: 10.1016/j.yfrne.2011.07.002 (PMC3336209; doi:10.1016/j.yfrne.2011.07.002)
Supplement: Supplementary references — References for Supplementary Tables. [file mmc10.doc]

**Supplementary information**

**References**

1. G. Aguilera, A. Kiss, X. Luo, Increased expression of type 1 angiotensin II receptors in the hypothalamic paraventricular nucleus following stress and glucocorticoid administration, J. Neuroendocrinol. 7 (1995) 775-783.
2. K.A. Al-Barazanji, S. Wislon, J. Baker, D.S. Jessop, M.S. Harbuz, Central orexin-A activates hypothalamic-pituitary-adrenal axis and stimulates hypothalamic corticotropin releasing factor and arginine vasopressin neurones in conscious rats, J. Neuroendocrinol. 13 (2001) 421-424.
3. M.J. Alexander, S.E. Leeman, Widespread expression in adult rat forebrain of mRNA encoding high-affinity neurotensin receptor, J. Comp. Neurol. 402 (1998) 475-500.
4. L.D. Alexander, L.D. Sander, Vasoactive intestinal peptide stimulates ACTH and corticosterone release after injection into the PVN, Regul. Pept. 51 (1994) 221-227.
5. L.D. Alexander, L.D. Sander, Involvement of vasopressin and corticotropin-releasing hormone in VIP- and PHI-induced secretion of ACTH and corticosterone, Neuropeptides 28 (1995) 167-173.
6. W.M. Al-Ghoul, R.B. Meeker, R.S. Greenwood, Kindled Seizures Increase Metabotropic Glutamate Receptor Expression and Function in the Rat Supraoptic Nucleus. J. Neurosci. Res. 54 (1998) 412–423.
7. C. Aoki, C.G. Go, C. Venkatesan, H. Kurose, Perikaryal and synaptic localization of alpha 2A-adrenergic receptor-like immunoreactivity, Brain Res. 650 (1994) 181-204.
8. N.M. Appel, W.M. Mitchell, R.K. Garlick, R.A. Glennon, M. Teitler, E.B. De Souza, Autoradiographic characterization of (±)-1-(2,5-dimethoxy-4-[125I]iodophenyl)-2-aminopropane (125I]DOI binding to 5-HT2 and 5-HT1C receptors in rat brain, J. Pharmacol. Exp. Ther. 255 (1990) 843-857.
9. H. Arima, T. Murase, K. Kondo, Y. Iwasaki, Y. Oiso, Centrally administered neuropeptide FF inhibits arginine vasopressin release in conscious rats, Endocrinology 137 (1996) 1523-1529.
10. I. Armando, S. Volpi, G. Aguilera, J.M. Saavedra, Angiotensin II AT_1_ receptor blockade prevents the hypothalamic corticotropin-releasing factor response to isolation stress, Brain Res. 1142 (2007) 92-99.
11. U. Arvidsson, M. Riedl, S. Chakrabarti, L. Vulchanova, J.H. Lee, A.H. Nakano, X. Lin, H.H. Loh, P.Y. Law, M.W. Wessendorf, R. Elde, The κ-opioid receptor is primarily postsynaptic: combined immunohistochemical localization of the receptor and endogenous opioids, Proc. Natl. Acad. Sci. USA 92 (1995) 5062-5066.
12. K. Azdad, R. Piet, D.A. Poulain, S.H.R. Oliet, Dopamine D4 receptor-mediated presynaptic inhibition of GABAergic transmission in the rat supraoptic nucleus, J. Neurophysiol. 90 (2003) 559-565.
13. J.S. Bains, A. Potyok, A.V. Ferguson, Angiotensin II actions in paraventricular nucleus: functional evidence for neurotransmitter role in efferents originating in subfornical organ, Brain Res. 599 (1992) 223-229.
14. D.V. Balmoukhametova, S.A. Hewitt, C.A. Sank, J.S. Bains, Dopamine modulates use-dependent plasticity of inhibitory synapses, J. Neurosci. 24 (2004) 5162-5171.
15. G. Banisadr, R.D. Gosselin, P. Mechighel, W. Rostène, P. Kitabgi, S. Mélik Parsadaniantz, Constitutive neuronal expression of CCR2 chemokine receptor and its colocalization with neurotransmitters in normal rat brain: functional effect of MCP-1/CCL2 on calcium mobilization in primary cultured neurons, J. Comp. Neurol. 492 (2005) 178-192.
16. J.R. Barson, A.J. Carr, J.E. Soun, N.C. Sobhani, P. Rada, S.F. Leibowitz, B.G. Hoebel, Opioids in the hypothalamic paraventricular nucleus stimulate ethanol intake, Alcoholism: Clin. Exp. Res. 34 (2010) 1-9.
17. J. Baulmann, H. Spitznagel, T. Herdegen, T. Unger, J. Culman, Tachykinin receptor inhibition and c-Fos expression in the rat brain following formalin-induced pain. Neuroscience, 95 (2000) 813-820.
18. S.L. Bealer, W.R. Crowley, Stimulation of central and systemic oxytocin release by histamine in the paraventricular nucleus: evidence for an interaction with norepinephrine, Endocrinology 140 (1999) 1158-1164.
19. S.L. Bealer, W.R. Crowley, Histaminergic control of oxytocin release in the paraventricular nucleus during lactation in rats, Exp. Neurol. 171 (2001) 317-322.
20. S.L. Bealer, D.L. Lipschitz, G. Ramoz, W.R. Crowley, Oxytocin receptor binding in the hypothalamus during gestation in rats, Am. J. Physiol. Regul. Integr. Comp. Physiol. 291 (2006) 53-58.
21. A. Beaudet, D. Greenspun, J. Raelson, G.S. Tannenbaum, Patterns of expression of SSTR1 and SSTR2 somatostatin receptor subtypes in the hypothalamus of the adult rat: relationship to neuroendocrine function, Neuroscience 65 (1995) 551-561.
22. C. Becskei, T. Riediger, D. Zünd, P. Wookey, T.A. Lutz, Immunohistochemical mapping of calcitonin receptors in the adult rat brain, Brain Res. 1030 (2004) 221-233.
23. R. Bernardini, A. Chiarenza, A.E. Calogero, P.W. Gold, G.P. Chrousos GP, Arachidonic acid metabolites modulate rat hypothalamic corticotropin-releasing hormone secretion in vitro, Neuroendocrinology 50 (1989) 708-715.
24. S. Bischoff, S. Leonhard, N. Reymann, V. Schuler, R. Shigemoto, K. Kaupmann, B. Bettler, Spatial distribution of GABA(B)R1 receptor mRNA and binding sites in the rat brain, J. Comp. Neurol. 412 (1999) 1-16.
25. R.S. Bitner, A.L. Nikkel, S. Otte, B. Martino, E.H. Barlow, P. Bhatia, A.O. Stewart, J.D. Brioni, M.W. Decker, R.B. Moreland, Dopamine D4 receptor signaling in the rat paraventricular hypothalamic nucleus: Evidence of natural coupling involving immediate early gene induction and mitogen activated protein kinase phosphorylation, Neuropharmacology 50 (2006) 521-531.
26. M.T. Bluet-Pajot, F. Presse, Z. Voko, C. Hoeger, F. Mounier, J. Epelbaum, J.L. Nahon, Neuropeptide-E-I antagonizes the action of melanin-concentrating hormone on stress-induced release of adrenocorticotropin in the rat, J. Neuroendocrinol. 7 (1995) 297-303.
27. A. Blume, O.J. Bosch, S. Miklos, L. Torner, L. Wales, M. Waldherr, I.D. Neumann, Oxytocin reduces anxiety via ERK1/2 activation: local effect within the rat hypothalamic paraventricular nucleus, Eur. J. Neurosci. 27 (2008) 1947-1956.
28. C. Boudaba, S. Di, J.G. Tasker, Presynaptic noradrenergic regulation of glutamate inputs to hypothalamic magnocellular neurons, J. Neuroendocrinol. 15 (2003) 803-810.
29. C. Boudaba, D.M. Linn, K.C. Halmos, J.G. Tasker, Increased tonic activation of presynaptic metabotropic glutamate receptors in the rat supraoptic nucleus following chronic dehydration, J. Physiol. 551 (2003) 815-823.
30. M.L. Bouthenet, E. Souil, M.P. Martres, P. Sokoloff, B. Giros, J.C. Schwartz, Localization of dopamine D3 receptor mRNA in the rat brain using in situ hybridization histochemistry: comparison with dopmaine D2 receptor mRNA, Brain Res. 564 (1991) 203-219.
31. E. Brailoiu, S.L. Dun, G.C. Brailoiu, K. Mizuo, L.A. Sklar, T.I. Oprea, E.R. Prossnitz, N.J. Dun, Distribution and characterization of estrogen receptor G protein-coupled receptor 30 in the rat central nervous system, J. Endocrinol. 193 (2007) 311-321.
32. C.H. Brown, P.J. Brunton, J.A. Russell, Rapid estradiol-17beta modulation of opioid actions on the electrical and secretory activity of rat oxytocin neurons in vivo, Neurochem. Res. 33 (2008) 614-623.
33. C.H. Brown, V. Scott, M. Ludwig, G. Leng, C.W. Bourque, Somatodendritic dynorphin release: orchestrating activity patterns of vasopressin release, Biochem. Soc. Trans. 35 (2007) 1236-1242.
34. T.O. Bruhn, S.W. Sutton, P.M. Plotsky, W.W. Vale, Central administration of corticotropin-releasing factor modulates oxytocin secretion in the rat, Endocrinology 119 (1986) 1558-1563.
35. T.C.D. Burazin, J.A. Larm, A.L. Gundlach, Regulation by osmotic stimuli of galanin-R1 receptor expression in magnocellular neurones of the paraventricular and supraoptic nuclei of the rat, J. Neuroendocrinol. 13 (2001) 358-370.
36. C. Callewaere, G. Banisadr, M.G. Desarménien, P. Mechighel, P. Kitabgi, W.H. Rostène, S. Mélik Parsadaniantz, The chemokine SDF-1/CXCL12 modulates the firing pattern of vasopressin neurons and counteracts induced vasopressin release through CXCR4, Proc. Natl. Acad. Sci. USA 103 (2006) 8221-8226.
37. C. Callewaere, B. Fernette, D. Raison, P. Mechighel, A. Burlet, A. Calas, P. Kitabgi, S.M. Parsadaniantz, W. Rostène, Cellular and subcellular evidence for neuronal interaction between the chemokine stromal cell-derived factor-1/CXCL 12 and vasopressin: regulation in the hypothalamo-neurohypophysial system of the Brattleboro rats, Endocrinology 149 (2008) 310-319.
38. A.E. Calogero, T.C. Kamilaris, M.T. Gomez, E.O. Johnson, M.E. Tartaglia, P.W. Gold, G.P. Chrousos, The muscarinic cholinergic agonist arecoline stimulates the rat hypothalamic-pituitary-adrenal axis through a centrally-mediated corticotropin-releasing hormone-dependent mechanism, Endocrinology 125 (1989) 2445-2453.
39. M.P. Castelli, A.P. Piras, T. Melis, S. Succu, F. Sanna, M.R. Melis, S. Collu, M.G. Ennas, G. Diaz, K. Mackie, A. Argiolas, Cannabinoid CB1 receptors in the paraventricular nucleus and central control of penile erection: immunocytochemistry, autoradiography and behavioural studies, Neuroscience 147 (2007) 197-206.
40. M.J. Cato, G.M. Toney, Angiotensin II excites paraventricular nucleus neurons that innervate the rostral ventrolateral medulla: an in vitro patch-clamp study in brain slices, J. Neurophysiol. 93 (2005) 403-413.
41. J.L. Charli, P. Joseph-Bravo, J.M. Palacios, C. Kordon, Histamine-induced release of thyrotropin releasing hormone from hypothalamic slices, Eur. J. Pharmacol. 52 (1978) 401-403.
42. G. Charron, S. Laforest, C. Gagnon, G. Drolet, D. Mouginot, Acute sodium deficit triggers plasticity of the brain angiotensin type 1 receptors, FASEB J. 16 (2002) 610-612.
43. X.Q. Chen, J.Z. Du, Y.S. Wang, Regulation of hypoxia-induced release of corticotropin-releasing factor in the rat hypothalamus by norepinephrine, Regul. Pept. 119 (2004) 221-228.
44. Q. Chen, D.P. Li, H.L. Pan, Presynaptic α1 adrenergic receptors differentially regulate synaptic glutamate and GABA release to hypothalamic presympathetic neurons, J. Pharmacol. Exp. Therap. 316 (2006) 733-742.
45. M.Y. Cheng, C.M. Bullock, C. Li, A.G. Lee, J.C. Bermak, J. Belluzzi, D.R. Weaver, F.M. Leslie, Q.Y. Zhou, Prokinectin 2 transmits the behavioural circadian rhythm of the suprachiasmatic nucleus, Nature 417 (2002) 405-410.
46. W. Chong, L.H. Li, K. Lee, M.H. Lee, J.B. Park, P.D. Ryu, Subtypes of alpha1- and alpha2-adrenoceptors mediating noradrenergic modulation of spontaneous inhibitory postsynaptic currents in the hypothalamic paraventricular nucleus, J. Neuroendocrinol. 16 (2004) 450-457.
47. J.Y. Chu, L.T Lee, C.H. Lai, H. Vaudry, Y.S. Chan, W.H. Yung, B.K. Chow, Secretin as a neurohypophyseal factor regulating water homeostasis, Proc. Natl. Acad. Sci. USA 106 (2009) 15961-15966.
48. J. Ciosek, K. Izdebska, Thyrotropin-releasing hormone modulates vasopressin and oxytocin synthesis and release from the hypothalamo-neurohypophysial system of different age male rats. J. Physiol. Pharmacol. 60 (2009) 63-70.
49. A. Cisowska-Maciejewska, J. Ciosek, Galanin influences vasopressin and oxytocin release from the hypothalamo-neurohypophysial system of salt-loaded rats, J. Physiol. Pharmacol. 56 (2005) 673-688.
50. J.E. Cluderay, D.C. Harrison, G.J. Hervieu, Protein distribution of the orexin-2 receptor in the rat central nervous system, Regul. Pept. 104 (2002) 131-144.
51. J.D. Coppola, B.A. Horwitz, J. Hamilton, R.B. McDonald, Expression of NPY Y1 and Y5 receptors in the hypothalamic paraventricular nucleus of aged Fischer 344 rats, Am. J. Physiol. Regul. Integr. Comp. Physiol. 287 (2004) 69-75.
52. T.L. Coventry, D.S. Jessop, D.P. Finn, M.D. Crabb, H. Kinoshita, M.S. Harbuz, Endomorphins and activation of the hypothalamo-pituitary-adrenal axis, J. Endocrinol. 169 (2001) 185-193.
53. J.W. Crane, K.M. Buller, Systemic blockade of complement C5a receptors reduces lipopolysacharride-induced responses in the paraventricular nucleus and the central amygdala, Neurosci. Lett. 424 (2007) 10-15.
54. J.W. Crane, K. Shimizu, G.A. Carrasco, F. Garcia, C. Jia, N.R. Sullivan, D.N. D'Souza, Y. Zhang, L.D. Van de Kar, N.A. Muma, G. Battaglia, 5-HT1A receptors mediate (+)8-OH-DPAT-stimulation of extracellular signal-regulated kinase (MAP kinase) in vivo in rat hypothalamus: time dependence and regional differences, Brain Res. 1183 (2007) 51-59.
55. J. Culman, S. Klee, C. Ohlendorf, T. Unger, Effect of tachykinin receptor inhibition in the brain on cardiovascular and behavioural responses to stress, J. Pharmacol. Exp. Ther. 280 (1997) 238-246.
56. S. Cummings, V. Seybold, Relationship of alpha-1 and alpha-2-adrenergic-binding sites to regions of the paraventricular nucleus of the hypothalamus containing corticotrophin-releasing factor and vasopressin neurons, Neuroendocrinology 47 (1988) 523-532.
57. A. Czyrak, A. Chocyk, M. Maćkowiak, K. Fijał, K. Wedzony, Distribution of dopamine D1 receptors in the nucleus paraventricularis of the hypothalamus in rats: an immunohistochemical study, Brain Res. Mol. Brain Res. 85 (2000) 209-217.
58. A.P. da Costa, X. Ma, C.D. Ingram, S.L. Lightman, G. Aguilera, Hypothalamic and amygdaloid corticotropin-releasing hormone (CRH) and CRH receptor-1 mRNA expression in the stress-hyporesponsive late pregnant and early lactating rat, Brain Res. Mol. Brain Res. 91 (2001) 119-130.
59. H.E.W. Day, S. Campeau, S.J. Watson, H. Akil, Expression of α1b adrenoceptor mRNA in corticotropin-releasing hormone-containing cells of the rat hypothalamus and its regulation by corticosterone, J. Neurosci. 19 (1999) 10098-10106.
60. G. Dayanithi, N. Sabatier, H. Widmer, Intracellular calcium signalling in magnocellular neurones of the rat supraoptic nucleus: understanding the autoregulatory mechanisms, Exp. Physiol. 85 (2000) 75-84.
61. M.C. Defagot, E.L. Malchiodi, M.J. Villar, M.C. Antonelli, Distribution of D4 dopamine receptor in rat brain with sequence-specific antibodies, Brain Res. Mol. Brain Res. 45 (1997) 1-12.
62. B. Depczynski, K. Nichol, Z. Fathi, T. Iismaa, J. Shine, A. Cunningham, Distribution and characterization of the cell types expressing GALR2 mRNA in brain and pituitary gland, Ann. N Y Acad. Sci. 863 (1998) 120-128.
63. E.B. De Souza, T.R. Insel, M.H. Perrin, J. Rivier, W.W. Vale, M.J. Kuhar, Corticotropin-releasing factor receptors are widely distributed within the rat central nervous system: an autoradiographic study, J. Neurosci. 5 (1995) 3189-3203.
64. P. de Souza Villa, J.V. Menani, G.M. de Arruda Camargo, L.A. de Arruda Camargo, W.A. Saad, Activation of the serotonergic 5-HT1A receptor in the paraventricular nucleus of the hypothalamus inhibits water intake and increases urinary excretion in water-deprived rats, Regul. Pept. 150 (2008) 14-20.
65. S. Di, C. Boudaba, I.R. Popescu, F.J. Weng, C. Harris, V.L. Marcheselli, N.G. Bazan, J.G. Tasker, Activity-dependent release and actions of endocannabinoids in the rat hypothalamic supraoptic nucleus. J. Physiol 569 (2005) 751-760.
66. S. Di, R. Malcher-Lopes, K.C. Halmos, J.G. Tasker JG, Nongenomic glucocorticoid inhibition via endocannabinoid release in the hypothalamus: a fast feedback mechanism, J. Neurosci. 23 (2003) 4850-4857.
67. Y.Q. Ding, B.Z. Lu, Z.L. Guan, D.S. Wang, J.Q. Xu, J.H. Li, Neurokinin B receptor (NK3)-containing neurons in the paraventricular and supraoptic nuclei of the rat hypothalamus synthesize vasopressin and express fos following intravenous injection of hypertonic saline, Neuroscience 91 (1999) 1077-1085.
68. A.V. Domyancic, D.A. Morilak, Distribution of alpha1A adrenergic receptor mRNA in the rat brain visualized by in situ hybridization, J. Comp. Neurol. 386 (1997) 358-378.
69. F. Dray, A. Wisner, M.C. Bommelaer-Bayet, C. Tiberghien, K. Gerozissis, M. Saadi, M.P. Junier, C. Rougeot, Prostaglandin E2, leukotriene C4, and platelet-activating factor receptor sites in the brain. Binding parameters and pharmacological studies, Ann. NY Acad. Sci. 559 (1989) 100-111.
70. Y. Dumont, R. Quirion, [^125^I]-GR231118: a high affinity radioligand to investigate neuropeptide Y Y1 and Y4 receptors, Br. J. Pharmacol. 129 (2000) 37-46.
71. M.M. Durkin, C.A. Gunwaldsen, B. Borowsky, K.A. Jones, T.A. Branchek, An in situ hybridization study of the distribution of the GABAB2 mRNA in the rat CNS, Mol. Brain Res. 71 (1999) 185-200.
72. T. Eguchi, Y. Takano, T. Hatae, R. Saito, Y. Nakayama, Y. Shigeyoshi, H. Okamura, J.E. Krause, H. Kamiya, Antidiuretic action of tachykinin NK-3 receptor in the rat paraventricular nucleus, Brain Res. 743 (1996) 49-55.
73. J. Espallergues, O. Solovieva, V. Techer, K. Bauer, G. Alonso, A. Vincent, N. Hussy, Synergistic activation of astrocytes by ATP and norepinephrine in the rat supraoptic nucleus, Neuroscience 148 (2007) 712-723.
74. N.K. Evanson, D.C. Van Hooren, J.P. Herman, GluR5-mediated glutamate signaling regulates hypothalamo-pituitary-adrenocortical stress responses at the paraventricular nucleus and median eminence, Psychoneuroendocrinology 34 (2009)1370-1379.
75. A. Fassio, G. Evans, R. Grisshammer, J.P. Bolam, M. Mimmack, P.C. Emson, Distribution of the neurotensin receptor NTS1 in the rat CNS studied using an amino-terminal directed antibody, Neuropharmacology 39 (2000) 1430-1442.
76. A. Fatima, M.F. Haroon, G. Wolf, M. Engelmann, M.G. Spina, Urocortin 1 administered into the hypothalamic supraoptic nucleus affects open-field behaviour in rats, Amino Acids 38 (2010) 1407-1414.
77. A. Fedelli, S Braconi, D. Economidou, N. Cannella, M. Kallupi, R. Guerrini, C. Calo, M. Massi, R. Ciccocioppo, The paraventricular nucleus of the hypothalamus is a neuroanatomical substrate for the inhibition of palatable food intake by neuropeptide S, Eur. J. Neurosci. 30 (2009) 1594-1602.
78. C. Fekete, J. Kelly, E. Mihaly, S. Sarkar, W.M. Rand, G. Legradi, C.H. Emerson, R.M. Lechan, Neuropeptide Y has a central inhibitory action on the hypothalamic-pituitary-thyroid axis, Endocrinology 142 (2001) 2602-2613.
79. C. Fekete, S. Sarkar, W.M. Rand, J.W. Harney, C.H. Emerson, A.C. Bianco, A. Beck-Sickinger, R.M. Lechan, Neuropeptide Y1 and Y5 receptors mediate the effects of neuropeptide Y on the hypothalamic-pituitary-thyroid axis, Endocrinology 143 (2002) 4513-4519.
80. A.V. Ferguson, Angiotensinergic regulation of autonomic and neuroendocrine outputs: critical roles for the subfornical organ and paraventricular nucleus, Neuroendocrinology 89 (2009) 370-376.
81. S.O. Fetissov, J. Kopp, T. Hokfelt, Distribution of NPY receptors in the hypothalamus, Neuropeptides 38 (2004) 175-188.
82. M.J. Follwell, A.V. Ferguson, Cellular mechanisms of orexin actions on paraventricular nucleus neurones in rat hypothalamus, J. Physiol. 545 (2002) 855-867.
83. R.T. Fremeau, G.E. Duncan, M.G. Fornaretto, A. Dearry, J.A. Gingrich, G.R. Breese, M.G. Caron, Localization of D1 dopamine receptor mRNA in brain supports a role in cognitive, affective, and neuroendocrine aspects of dopaminergic neurotransmission, Proc. Natl. Acad. Sci. USA 88 (1991) 3772-3776.
84. S. Fukusumi, H. Yoshida, R. Fujii, M. Maruyama, H. Komatsu, Y. Habata, Y. Shintani, S. Hinuma, M. Fujino, A new peptidic ligand and its receptor regulating adrenal function in rats, J. Biol. Chem. 278 (2003) 46387-46395.
85. M.M. Garrido, J. Manzanares, J.A. Fuentes, Hypothalamus, anterior pituitary and adrenal gland involvement in the activation of adrenocorticotropin and corticosterone secretion by gastrin-releasing peptide, Brain Res. 828 (1999) 20-26.
86. M.M. Garrido, S. Martin, E. Ambrosio, J.A. Fuentes, J. Manzanares, Role of corticotropin-releasing hormone in gastrin-releasing peptide-mediated regulation of corticotropin and corticosterone secretion in male rats, Neuroendocrinology 68 (1998) 116-122.
87. J.C. Garza, C.S. Kim, J. Liu, W. Zhang, X.Y. Lu, Adeno-associated virus-mediated knockdown of melanocortin-4 receptor in the paraventricular nucleus of the hypothalamus promotes high-fat diet-induced hyperphagia and obesity, J. Endocrinol. 197 (2008) 471-482.
88. S.R. George, R.L. Zastawny, R. Briones-Urbina, R. Cheng, T. Nguyen, M. Heiber, A. Kouvelas, A.S Chan, B.F. O’Dowd. Distinct distributions of mu, delta and kappa opioid receptor mRNA in rat brain. Biochim. Biophys. Res. Commun. 205 (1994) 1438-1444.
89. M. Ghamari-Langroudi, C.W. Bourque, Muscarinic receptor modulation of slow afterhyperpolarization and phasic firing in rat supraoptic nucleus neurons, J. Neurosci. 24 (2004) 7718-7726.
90. E.R. Gillard, C.G. Coburn, A. de Leon, E.P. Snissarenko, L.G. Bauce, Q.J. Pittman, B. Hou, M.C. Curras-Collazo, Vasopressin autoreceptors and nitric oxide-dependent glutamate release from rat magnocellular neuroendocrine cells responding to osmotic stimuli, Endocrinology 148 (2007) 479-489.
91. E.R. Gillard, M. Leon-Olea, S. Mucio-Ramirez, C.G. Coburn, E. Sanchez-Islas, A. de Leo, H. Mussenden, L.G. Bauce, Q.J. Pittman, M.C. Curras-Collazo, A novel role for endogenous pituitary adenylate cyclase activating poplypeptide in the magnocellular neuroendocrine system, Endocrinology 147 (2006) 791-803.
92. S.Q. Giraudo, C.J. Billington, A.S. Levine, Feeding effects of hypothalamic injection of melanocortin 4 receptor ligands, Brain Res. 809 (1998) 302-306.
93. M. Goebel, A. Stengel, L. Wang, T. Coskun, J. Alsina-Fernandez, J. Rivier, Y. Taché, Pattern of Fos expression in the brain induced by selective activation of somatostatin receptor 2 in rats, Brain Res. 1351 (2010) 150-164.
94. R. Goke, P.J. Larsen, J.D. Mikkelsen, S.P. Sheikh, Distribution of GLP-1 binding sites in the rat brain: evidence that exendin-4 is a ligand of brain GLP-1 binding sites, Eur. J. Neurosci. 7 (1995) 2294-2300.
95. B.A. Gosnell, J.E. Morley, A.S. Levine, Opioid-induced feeding: localization of sensitive brain sites, Brain Res. 369 (1986) 177-184.
96. C. Gouarderes, I. Quelven, C. Mollereau, H. Mazarguil, S.Q. Rice, J.M. Zajac, Quantitative autoradiographic distribution of NPFF1 neuropeptide FF receptor in the rat brain and comparison with NPFF2 receptor by using [125I]YVP and [125I]EYF as selective radioligands, Neuroscience 115 (2002) 349-361.
97. L. Gouzénes, N. Sabatier, P. Richard, F.C. Moos, G. Dayanithi, V1a- and V2-type vasopressin receptors mediate vasopressin-induced Ca2+ responses in isolated rat supraoptic neurones, J. Physiol. 517 (1999) 771-779.
98. X.M. Guan, H. Yu, Q. Jiang, L.H. Van Der Ploeg, Q. Liu, Distribution of neuromedin U receptor subtype 2 mRNA in the rat brain, Brain. Res. Gene. Expr. Patterns 1 (2001) 1–4.
99. X.M. Guan, H. Yu, O.C. Palyha, K.K. McKee, S.D. Feighner, D.J. Sirinathsinghji, R.G. Smith, L.H. Van der Ploeg, A.D. Howard, Distribution of mRNA encoding the growth hormone secretagogue receptor in brain and peripheral tissues, Brain Res. Mol. Brain Res. 48 (1997) 23-29.
100. A.L. Gundlach, T.C. Burazin, Galanin-galanin receptor systems in the hypothalamic paraventricular and supraoptic nuclei. Some recent findings and future challenges, Ann. NY Acad. Sci. 863 (1998) 241-251.
101. E.L. Gustafson, M.M Durkin, J.A. Bard, J. Zgombick, T.A. Branchek, A receptor autoradiographic and in situ hybridization analysis of the distribution of the 5-ht7 receptor in rat brain. Br. J. Pharmacol. 117 (1996) 657-666.
102. G.E. Haley, F.W. Flynn, Agonist and hypertonic saline-induced trafficking of the NK3-receptors on vasopressin neurons within the paraventricular nucleus of the hypothalamus, Am. J. Physiol. Integr. Comp. Physiol. 290 (2006) 1242-1250.
103. G.E. Haley, F.W. Flynn, Blockade of NK3R signalling in the PVN decreases vasopressin and oxytocin release and c-fos expression in the magnocellular neurons in response to hypotension, Am. J. Physiol. Regul. Integr. Comp. Physiol. 295 (2008) 1158-1167.
104. J.A. Harrold, T. Dovey, X.J. Cai, J.C. Halford, J. Pinkney, Autoradiographic analysis of ghrelin receptors in the rat hypothalamus, Brain Res. 1196 (2008) 59-64.
105. H. Hashimoto, S. Hyodo, M. Kawasaki, M. Shibata, T. Saito, H. Suzuki, H. Otsubo, T. Yokoyama, H. Fujihara, T. Higuchi, Y. Takei, Y. Ueta, Adrenomedullin 2 (AM2)/intermedin is a more potent activator of hypothalamic oxytocin-secreting neurons than AM possibly through an unidentified receptor in rats, Peptides 28 (2007) 1104-1112.
106. H. Hashimoto, T. Onaka, M. Kawasaki, L. Chen, T. Mera, A. Soya, T. Saito, H. Fujihara, H. Sei, Y. Morita, Y. Ueta, Effects of cholecysokinin (CCK)-8 on hypothalamic oxytocin-secreting neurons in rats lacking CCK-A receptor, Auton. Neurosci. 121 (2005) 16-25.
107. G.I. Hatton, Q.Z. Yang, Ionotropic histamine receptors and H2 receptors modulate supraoptic oxytocin neuronal excitability and dye coupling, J. Neurosci. 21 (2001) 2974-2982.
108. G.G. Hazell, S.T.Yao, J.A. Roper, E.R. Prossnitz, A-M. O'Carroll, S.J. Lolait, Localisation of GPR30, a novel G protein-coupled oestrogen receptor, suggests multiple functions in rodent brain and peripheral tissues, J. Endocrinol. 202 (2009) 223-236.
109. S.C. Heinrichs, F. Menzaghi, E.M. Pich, R.L. Hauger, G.F. Koob, Corticotropin-releasing factor in the paraventricular nucleus modulates feeding induced by neuropeptide Y, Brain Res. 611 (1993) 18-24.
110. L.K. Heisler, N. Pronchuk, K. Nonogaki, L. Zhou, J. Raber, L. Tung, G.S. Yeo, S. O’Rahilly, W.F. Colmers, J.K. Elmquist, L.H. Tecott, Serotonin activates the hypothalamic-pituitary-adrenal axis via serotonin 2C receptor stimulation, J. Neurosci. 27 (2007) 6956-6964.
111. M. Herkenham, A.B. Lynn, M.R. Johnson, L.S. Melvin, B.R. de Costa, K.C. Rice, Characterization and localization of cannabinoid receptors in rat brain: a quantitative in vivo autoradiographic study, J. Neurosci. 11 (1991) 563-583.
112. G.J. Hervieu, J.E. Cluderay, D. Harrison, J. Meakin, P. Maycox, S. Nasir, R.A. Leslie, The distribution of the mRNA and protein products of the melanin-concentrating hormone (MCH) receptor gene, slc-1, in the central nervous system of the rat, Eur. J. Neurosci. 12 (2000) 1194-1216.
113. G.J. Hervieu, J.E. Cluderay, D.C. Harrison, J.C. Roberts, R.A. Leslie, Gene expression and protein distribution of the orexin-1 receptor in the rat brain and spinal cord, Neuroscience 103 (2001) 777-797.
114. J.M. Hilton, S.Y. Chai, P.M. Sexton, In vitro autoradiographic localization of the calcitonin receptor isoforms, C1a and C1b, in rat brain, Neuroscience 69 (1995) 1223-1237.
115. C. Hindmarch, S. Yao, G. Beighton, J. Paton, D. Murphy, A comprehensive description of the transcriptome of the hypothalamoneurohypophyseal system in euhydrated and dehydrated rats, Proc. Natl. Acad. Sci. USA 103 (2006) 1609-1614.
116. G.L. Hinks, J.A. Poat, J. Hughes, Changes in hypothalamic cholecystokinin A and cholecysotkinin B receptor subtypes and associated neuropeptide expression in response to salt-stress in the rat and mouse, Neuroscience 68 (1995) 765-781.
117. T. Honda, E. Wada, J.F. Battey, S.A Wank, Differential Gene Expression of CCK(A) and CCK(B) Receptors in the Rat Brain, Mol. Cell. Neurosci. 4 (1993) 143-154.
118. T.D. Hou, J.Z. Du, Norepinephrine attenuates hypoxia-inhibited thyrotropin-releasing hormone release in median eminence and paraventricular nucleus of rat hypothalamus, Neuro. Endocrinol. Lett. 26 (2005) 43-49.
119. H.E. Howe, S.J. Somponpon, C.D. Sladek, Role of neurokinin 3 receptors in supraoptic vasopressin and oxytocin neurons, J. Neurosci. 24 (2004) 10103-10110.
120. Q. Huang, E. Timofeeva, D. Richard, Regulation of corticotropin-releasing factor and its types 1 and 2 receptors by leptin in rats subjected to treadmill running-induced stress, J. Endocrinol. 191 (2006) 179-188.
121. A. Hurbin, L. Boissin-Agasse, H. Orcel, A. Rabié, N. Joux, MG. Desarménien, P. Richard, F.C. Moos, The V1a and V1b, but not V2, vasopressin receptor genes are expressed in the supraoptic nucleus of the rat hypothalamus, and the transcripts are essentially colocalized in the vasopressinergic magnocellular neurons, Endocrinology 139 (1998) 4701-4707.
122. A. Hurbin, H. Orcel, G. Alonso, F. Moos, A. Rabié, The vasopressin receptors colocalize with vasopressin in the magnocellular neurons of the rat supraoptic nucleus and are modulated by water balance, Endocrinology 143 (2002) 456-466.
123. N. Ibrahim, I Shibuya, N. Kabashima, S.V. Sutarmo, Y. Ueta, H. Yamashita H, Prostaglandin E2 inhibits spontaneous inhibitory postsynaptic currents in rat supraoptic neurones via presynaptic EP receptors, J. Neuroendocrinol. 11 (1999) 879-886.
124. A. Ichimura, A. Hirasawa, T. Hara, G. Tsujimoto, Free fatty acid receptors act as nutrient sensors to regulate energy homeostasis, Prostaglandins Other Lipid Mediat. 89 (2009) 82-88.
125. K. Iitake, L. Share, Y. Ouchi, J.T. Crofton, D.P. Brooks, Central cholinergic control of vasopressin release in conscious rats, Am. J. Physiol. 251 (1986) 146-150.
126. T. Imaki, H. Katsumata, S.I. Konishi, Y. Kasagi, S. Minami, Corticotropin-releasing factor type-1 receptor mRNA is not induced in mouse hypothalamus by either stress or osmotic stimulation, J Neuroendocrinology 15 (2003) 916-924.
127. T. Imaki, H. Katsumata, M. Miyata, M. Naruse, J. Imaki, S. Minami, Expression of corticotropin-releasing hormone type 1 receptor in paraventricular nucleus after acute stress, Neuroendocrinology 73 (2001) 293-301.
128. S. Ishizaki, T. Murase, Y. Sugimura, S. Kakiya, H. Yokoi, K. Tachikawa, H. Arima, Y. Miura, Y. Oiso, Role of ghrelin in the regulation of vasopressin release in conscious rats, Endocrinology 143 (2002) 1589-1593.
129. S. Jegou, D. Cartier, C. Dubessy, B.J. Gonzalez, D. Chatenet, H. Tostivint, E. Scalbert, J. Leprince, H. Vaudry, I. Lihrmann, Localization of the urotensin II receptor in the rat central nervous system, J. Comp. Neurol. 495 (2006) 21-36.
130. C.A. Jennings, D.C. Harrison, P.R. Maycox, B. Crook, D. Smart, G.J. Hervieu, The distribution of the orphan bombesin receptor subtype-3 in the rat CNS, Neuroscience 120 (2003) 309-324.
131. D.S. Jessop, D. Renshaw, P.J. Larsen, H.S. Chowdrey, M.S. Harbuz, Substance P is involved in terminating the hypothalamo-pituitary-adrenal axis response to acute stress through centrally located neurokinin-1 receptors, Stress 3 (2000) 209-220.
132. J.H. Jhamandas, D. MacTavish, K.H. Harris, Neuropeptide FF (NPFF) control of magnocellular neurosecretory cells of the rat hypothalamic paraventricular nucleus (PVN), Peptides 27 (2006) 973-979.
133. M. Jhanwar-Uniyal, C.R. Roland, S.F. Leibowitz SF, Diurnal rhythm of alpha 2-noradrenergic receptors in the paraventricular nucleus and other brain areas: relation to circulating corticosterone and feeding behavior, Life Sci. 38 (1986) 473-482.
134. J.H. Jjamandas, D. MacTavish, Central administration of neuropeptide FF causes activation of oxytocin paraventricular hypothalamic neurones that project to the brainstem, J. Neuroendocrinol. 15 (2003) 24-32.
135. C.D. John, F.N. Gavins, N.A. Buss, P.O. Cover, J.C. Buckingham. Annexin A1 and the formyl peptide receptor family: neuroendocrine and metabolic aspects. Curr. Opin. Pharmacol. 8 (2008) 765-776.
136. K.M. Joo, Y.H. Chung, M.K. Kim, R.H. Nam, B.L. Lee, K.H. Lee, C.I. Cha, Distribution of vasoactive intestinal peptide and pituitary adenylate cyclase-aqctivating polypeptide receptors (VPAC1, VPAC2, and PAC1 receptor) in the rat brain, J. Comp. Neurol. 476 (2004) 388-413.
137. M. Juszczak, E. Boczek-Leszczyk, B. Stempniak, Effect of melatonin on the vasopressin secretion as influenced by tachykinin NK-1 receptor agonist and antagonists: in vivo and in vitro studies, J. Physiol Pharmacol. 58 (2007) 829-843.
138. M. Juszczak, B. Stempniak, Melatonin inhibits the substance P-induced secretion of vasopressin and oxytocin from the rat hypothalamo-neurohypophysial system: in vitro studies, Brain Res. Bull. 59 (2003) 393-397.
139. K. Kageyama, Y. Kumata, K. Akimoto, S. Takayasu, N. Tamasawa, T. Suda, Ghrelin stimulates corticotropin-releasing factor and vasopressin gene expression in rat hypothalamic 4B cells, Stress (2011) In press.
140. S.P. Kalra, P.S. Kalra, NPY and cohorts in regulating appetite, obesity and metabolic syndrome: beneficial effects of gene therapy, Neuropeptides 38 (2004) 201-211.
141. J. Kampe, P. Wiedmer, P.T. Pfluger, T.R. Castaneda, L. Burget, H. Mondala, J. Kerr, C. Liaw, B.J. Oldfield, M.H. Tschöp, D. Bagnol, Effect of central administration of QRFP(26) peptide on energy balance and characterization of a second QRFP receptor in rat, Brain Res. 1119 (2006) 133-149.
142. Y.M. Kang, Y. Ma, C. Elks, J.P. Zheng, Z.M. Yang, J. Francis, Cross-talk between cytokines and renin-angiotensin in hypothalamic paraventricular nucleus in heart failure: role of nuclear factor-kappaB, Cardiovasc. Res. 79 (2008) 671-678.
143. J.R. Kapoor, C.D. Sladek, Purinergic and adrenergic agonists synergize in stimulating vasopressin and oxytocin release, J. Neurosci. 20 (2000) 8868–8875.
144. J.R. Kapoor, C.D. Sladek, Substance P and NPY differentially potentiate ATP and adrenergic stimulated vasopressin and oxytocin release, Am. J. Physiol. Regul. Integr. Comp. Physiol. 280 (2001) 69-78.
145. M. Kawasaki, T.A. Ponzio, C. Yue, R.L. Fields, H. Gainer, Neurotransmitter regulation of c-fos and vasopressin gene expression in the rat supraoptic nucleus, Exp. Neurol. 219 (2009) 212-222.
146. A.R. Kennedy, J.F. Todd, W.S. Dhillo, L.J. Seal, M.A. Ghatei, C.P. O'Toole, M. Jones, D. Witty, K. Winborne, G. Riley, G. Hervieu, S. Wilson, S.R. Bloom, Effect of direct injection of melanin-concentrating hormone into the paraventricular nucleus: further evidence for a stimulatory role in the adrenal axis via SLC-1, J. Neuroendocrinol. 15 (2003) 268-272.
147. Z.U. Khan, A. Gutiérrez, R. Martín, A. Peñafiel, A. Rivera, A. de la Calle, Dopamine D5 receptors of rat and human brain, Neuroscience 100 (2000) 689-699.
148. M.S. Kim, C.J. Small, S.H. Russell, D.G. Morgan, C.R. Abbott, S.H. alAhmed, D.L. Hay, M.A. Ghatei, D.M. Smith, S.R. Bloom, Effects of melanocortin receptor ligands on thyrotropin-releasing hormone release: evidence for the differential roles of melanocortin 3 and 4 receptors, J. Neuroendocrinol. 14 (2002) 276-282.
149. A. Kinoshita, R. Shigemoto, H. Ohishi, H. van der Putten , N. Mizuno, Immunohistochemical localization of metabotropic glutamate receptors, mGlu7a and mGlu7b, in the central nervous system of the adult rat and mouse: a light and electron microscopic study, J. Comp. Neurol. 393 (1998) 332-352.
150. J.M. Kinzie, J.A. Saugstad, G.L.Westbrook, T.P. Segerson, Distribution of metabotropic glutamate receptor 7 messenger mRNA in the developing and adult rat brain, Neuroscience 69 (1995) 167-176.
151. T. Kishi, C.J. Aschkenasi, C.E. Lee, K.G. Mountjoy, C.B. Saper, J.K. Elmquist, Expression of melanocortin 4 receptor mRNA in the central nervous system of the rat, J. Comp. Neurol. 457 (2003) 213-235.
152. A. Kiss, G. Aguilera, Role of alpha-1-adrenergic receptors in the regulation of corticotropin-releasing hormone mRNA in the paraventricular nucleus of the hypothalamus during stress, Cell Mol. Neurobiol. 20 (2000) 683-694.
153. R.M Kobayashi, M. Palkovits, R.E. Hruska, R. Rothschild, H.I. Yamamura, Regional distribution of muscarinic cholinergic receptors in rat brain, Brain Res. 154 (1978) 13-23.
154. K. Kocsis, J. Kiss, T. Görcs, B. Halász, Metabotropic glutamate receptor in vasopressin, CRF and VIP hypothalamic neurones, NeuroReport 9 (1998) 4029–4033.
155. M. Kohzuki, S.Y. Chai, G. Paxinos, A. Karavas, D.J. Casley, C.I. Johnston, F.A. Mendelsohn, Localization and characterization of endothelin receptor binding sites in the rat brain visualized by in vitro autoradiography, Neuroscience 42 (1991) 245-260.
156. S. Koirala, G. Corfas, Identification of novel glial genes by single-cell transcriptional profiling of Bergmann glial cells from mouse cerebellum, PLoS One 5 (2010) e9198.
157. S.I. Konishi, Y. Kasagi, H. Katsumata, S. Minami, T. Imaki, Regulation of corticotropin-releasing factor (CRF) type 1 receptor gene expression by CRF in the hypothalamus, Endocr. J. 50 (2003) 21-36.
158. E. Kosa, A. Marcilhac-Flouriot, M.P. Fache, P. Siaud, Effects of β-phenylethylamine on the hypothalamo-pituitary-adrenal axis in the male rat, Pharmacol. Biochem. Behav. 67 (2000) 527-535.
159. M.G. Kozoriz, J.B. Kuzmiski, M. Hirasawa, Q.J. Pittman, Galanin modulates neuronal and synaptic properties in the rat supraoptic nucleus in a use and state dependent manner, J. Neurophysiol. 96 (2006) 154-164.
160. U. Kumar, Colocalization of somatostatin receptor subtypes (SSTR1-5) with somatostatin, NADPH-diaphorase (NADP-d), and tyrosine hydroxylase in the rat hypothalamus, J. Comp. Neurol. 504 (2007) 185-205.
161. K. Kurokawa, H. Yamada, J. Ochi, Topographical distribution of neurons containing endothelin type A receptor in the rat brain, J. Comp. Neurol. 389 (1997) 348-360.
162. S.E. Kyrkouli, B.G. Stanley, R.D. Seirafi, S.F. Leibowitz, Stimulation of feeding by galanin: anatomical localization and behavioral specificity of this peptide's effects in the brain, Peptides 11 (1990) 995-1001.
163. E.E. Ladenheim, R.T. Jensen, S.A. Mantey, T.H. Moran, Distinct distributions of two bombesin receptor subtypes in the rat central nervous system, Brain Res. 593 (1992) 168-178.
164. M. Lafarga, M.T. Berciano, E. Del Olmo, M.A. Andres, A. Pazos, Osmotic stimulation induces changes in the expression of beta-adrenergic receptors and nuclear volume of astrocytes in supraoptic nucleus of the rat, Brain Res. 588 (1992) 311-316.
165. M.C. Lagerström, N. Rabe, T. Haitina, I. Kalnina, A.R. Hellström, J. Klovins, K. Kullander, H.B. Schiöth, The evolutionary history and tissue mapping of GPR123: specific CNS expression pattern predominantly in thalamic nuclei and regions containing large pyramidal cells, J. Neurochem. 100 (2007) 1129-1142.
166. R.C. Lambert, G. Dayanithi, F.C. Moos, P. Richard, A rise in the intracellular Ca2+ concentration of isolated rat supraoptic cells in response to oxytocin, J. Physiol. 478 (1994) 275-287.
167. P.J. Larsen, M. Tang-Christensen, D.S. Jessop, Central administration of glucagon-like peptide-1 activates hypothalamic neuroendocrine neurons in the rat, Endocrinology 138 (1997) 4445-4455.
168. K.J. Latchford, A.V. Ferguson, ANG II-induced excitation of paraventricular nucleus magnocellular neurons: a role for glutamate interneurons, Am. J. Physiol. Regul. Integr. Comp. Physiol. 286 (2004) 894-902.
169. D.K. Lee, S.R. George, R. Cheng, T. Nguyen, Y. Liu, M. Brown, K.R. Lynch, B.F. O'Dowd, Identification of four novel human G protein-coupled receptors expressed in the brain, Brain Res. Mol. Brain Res. 86 (2001) 13-22.
170. K.S. Lee, T.H. Han, J.Y. Jo, G. Kang, S.Y. Lee, P.D. Ryu, J.H. Im, B.H. Jeon, J.B. Park, Serotonin inhibits GABA synaptic transmission in presympathetic paraventricular nucleus neurons, Neurosci. Lett. 439 (2008) 138-142.
171. D.K. Lee, T. Nguyen, C.A. Porter, R. Cheng, S.R. George, B.F. O'Dowd, Two related G protein-coupled receptors: the distribution of GPR7 in rat brain and the absence of GPR8 in rodents, Brain Res. Mol. Brain Res. 71 (1999) 96-103.
172. T.T. Lee, V.A. Redila, M.N. Hill, B.B. Gorzalka, 5-HT(2A) receptor mediated neuronal activation within the paraventricular nucleus of the hypothalamus is desensitized following prolonged glucocorticoid treatment, Eur. J. Pharmacol. 602 (2009) 54–57.
173. J.D. Legget, M.S. Harbuz, D.S. Jessop, A.J. Fulford, The nociceptin receptor antagonist [NPhe^1^,Arg1^4^,Lys^15^]nociceptin/orphanin FQ-NH_2_ blocks stimulatory effects of nociceptin/orphanin FQ on the HPA axis in rats, Neuroscience 141 (2006) 2051-2057.
174. S.F. Leibowitz, C. Sladek, L. Spencer, D. Tempel, Neuropeptide Y, epinephrine and norepinephrine in the paraventricular nucleus: stimulation of feeding and the release of corticosterone, vasopressin and glucose, Brain Res. Bull. 21 (1988) 905-912.
175. S.K. Leonard, R.H. Ring, Immunohistochemical localization of the neuropeptide S receptor in the rat central nervous system, Neuroscience 172 (2011) 153-163.
176. P. Leroux, B.J. Gonzales, A. Laquerriere, C. Bodenant, H. Vaudry, Autoradiographic study of somatostatin receptors in the rat hypothalamus: validation of a GTP-induced desaturation procedure, Neuroendocrinology 47 (1988) 533-544.
177. B.M. Lewis, C. Dieguez, M.D. Lewis, M.F. Scanlon, Dopamine stimulates release of thyrotrophin-releasing hormone from perfused intact rat hypothalamus via hypothalamic D2-receptors, J. Endocrinol. 115 (1987) 419-424.
178. D.P. Li, L.M. Atnip, S.R. Chen, H.L. Pan, Regulation of synaptic inputs to paraventricular-spinal output neurons by α2 adrenergic receptors, J. Neurophysiol. 93 (2005) 393-402.
179. D.P. Li, S.R. Chen, H.L. Pan, Adenosine Inhibits Paraventricular Presympathetic Neurons through ATP-dependent Potassium Channels, J. Neurochem. 113 (2010) 530-542.
180. X. Li, M. Fan, L. Shen, Y. Cao, D. Zhu, Z. Hong, Excitatory responses of cardiovascular activities to urocortin3 administration into the PVN of the rat, Auton Neurosci. 154 (2010) 108-111.
181. Q. Li, N.A. Muma, G. Battaglia, L.D. Van De Kar, A desensitisation of hypothalamic 5-HT1A receptors by repeated injections of paroxetine: reduction in the levels of Gi and Go proteins and neuroendocrine responses, but not in the density of 5-HT1A receptors, J. Pharmacol. Exp. Ther. 282 (1997) 1581-1590.
182. D.P. Li, H.L. Pan, Increased group I metabotropic glutamate receptor activity in paraventricular nucleus supports elevated sympathetic vasomotor tone in hypertension, Am. J. Physiol. Regul. Integr. Comp. Physiol. 299 (2010) 552-561.
183. W. Lichtensteiger, B. Hanimann, W. Siegrist, A.N. Eberle, Region- and stage-specific patterns of melanocortin receptor ontogeny in rat central nervous system, cranial nerve ganglia and sympathetic ganglia, Brain Res. Dev. Brain Res. 91 (1996) 93-110.
184. M. Lintunen, T. Sallmen, K. Karlstedt, H. Fukui, K.S. Eriksson, P. Panula, Postnatal expression of H1-receptor mRNA in the rat brain: correlation to L-histidine decarboxylase expression and local upregulation in limbic seizures, Eur. J. Neurosci. 10 (1998) 2287-2301.
185. Q. Liu, X.M. Guan, W.J. Martin, T.P. McDonald, M.K. Clements, Q. Jiang, Z. Zeng, M. Jacobson, D.L. Williams, H. Yu, D. Bomford, D. Figueroa, J. Mallee, R.Wang, J. Evans, R. Gould, C.P. Austin, Identification and characterization of novel mammalian neuropeptide FF-like peptides that attenuate morphine-induced antinociception, J. Biol. Chem. 276 (2001) 36961-36969.
186. M. Liu, R.M. Parker, K. Darby, H.J. Eyre, N.G. Copeland, J. Crawford, D.J. Gilbert, G.R. Sutherland, N.A. Jenkins, H. Herzog, GPR56, a novel secretin-like human G-protein-coupled receptor gene, Genomics 55 (1999) 296-305.
187. X. Liu, E. Tribollet, M. Raggenbass, GABAB receptor-activation inhibits GABAergic synaptic transmission in parvocellular neurones of rat hypothalamic paraventricular nucleus, J. Neuroendocrinol. 18 (2006) 177-186.
188. X.Y. Lu, G.S. Barsh, H. Akil, S.J. Watson, Interaction between α-melanocyte-stimulating hormone and corticotropin-releasing hormone in the regulation of feeding and hypothalamo-pituitary-adrenal responses, J. Neurosci. 23 (2003) 7863-7872.
189. M. Ludwig, Dendritic release of vasopressin and oxytocin, J. Neuroendocrinol. 10 (1998) 881-895.
190. X. Luo, A. Kiss, G. Makara, S.J. Lolait, G. Aguilera, Stress-specific regulation of corticotropin releasing hormone receptor expression in the paraventricular and supraoptic nuclei of the hypothalamus in the rat, J. Neuroendocrinol. 6 (1994) 689-696.
191. X. Luo, A. Kiss, C. Rabadan-Diehl, G. Aguilera, Regulation of hypothalamic and pituitary corticotropin-releasing hormone receptor messenger ribonucleic acid by adrenalectomy and glucocorticoids, Endocrinology. 136 (1995) 3877-3883.
192. E.M. Lutz, W.J. Sheward, K.M. West, J.A. Morrow, G. Fink, A.J. Harmar, The VIP2 receptor: molecular characterisation of a cDNA encoding a novel receptor for vasoactive intestinal peptide, FEBS Lett. 334 (1993) 3-8.
193. B. Lutz-Bucher, D. Monnier, B. Koch, Evidence for the presence of receptors for pituitary adenylate cyclase-activating polypeptide in the neurohypophysis that are positively coupled to cyclic AMP formation and neurohypophyseal hormone secretion, Neuroendocrinology 64 (1996) 153-161.
194. D.R. Lynch, M.W. Walker, R.J. Miller, S.H. Snyder SH, Neuropeptide Y receptor binding sites in rat brain: differential autoradiographic localizations with 125I-Peptide YY and 125I-neuropeptide Y imply receptor heterogeneity, J. Neurosci. 9 (1989) 2607-2619.
195. S. Ma, P.J. Shen, T.C. Burazin, G.W. Tregear, A.L. Gundlach, Comparative localization of leucine-rich repeat-containing G-protein-coupled receptor-7 (RXFP1) mRNA and [33P]-relaxin binding sites in rat brain: restricted somatic co-expression a clue to relaxin action? Neuroscience 141 (2006) 329-344.
196. M. Macova, J. Pavel, J.M. Saavedra, A peripherally administered, centrally acting angiotensin II AT2 antagonist selectively increases brain AT1 receptors and decreases brain tyrosine hydroxylase transcription, pituitary vasopressin and ACTH, Brain Res. 1250 (2009) 130-140.
197. H. Maeno, H. Kiyama, M. Tohyama, Distribution of the substance P receptor (NK-1 receptor) in the central nervous system, Brain Res. Mol. Brain Res. 18 (1993) 43-58.
198. I.G. Makarenko, M.M Meguid, M.V Ugrumov, Distribution of serotonin 5-hydroxytriptamine 1B (5-HT1B) receptors in the normal rat hypothalamus, Neurosci. Lett. 328 (2002) 155–159.
199. A. Mansour, C.A. Fox, S. Burke, H. Akil, S.J. Watson, Immunohistochemical localization of the cloned μ opioid receptor in the rat CNS, J. Chem. Neuroanat. 8 (1995) 283-305.
200. A. Mansour, C.A. Fox, S. Burke, F. Meng, R.C. Thompson, H. Akil, S.J. Watson, Mu, delta, and kappa opioid receptor mRNA expression in the rat CNS: an in situ hybridization study. J. Comp. Neurol. 350 (1994) 412-438.
201. A. Mansour, H. Khachaturian, M.E. Lewis, H. Akil, S.J. Watson, Autoradiographic differentiation of mu, delta, and kappa opioid receptors in the rat forebrain and midbrain, J. Neurosci. 7 (1987) 2445-2464.
202. J.N. Marcus, C.J. Aschkenasi, C.E. Lee, R.M. Chemelli, C.B. Saper, M. Yanagisawa, J.K. Elmquist, Differential expression of orexin receptors 1 and 2 in the rat brain, J. Comp. Neurol. 435 (2001) 6-25.
203. M. Margeta-Mitrovic, I. Mitrovic, R.C. Riley, L.Y. Jan, A.I. Basbaum, Immunohistochemical localization of GABA(B) receptors in the rat central nervous system, J. Comp. Neurol. 405 (1999) 299-321.
204. S.S. Marroni, F.N. Nakano, C.D.C. Gati, J.A.C. Oliveira, J. Antunes-Rodrigues, N. Garcia-Cairasco, Neuroanatomical and cellular substrates of hypergrooming induced by microinjection of oxytocin in central nucleus of amygdala, an experimental model of compulsive behaviour, Mol. Psychiatry. 12 (2007) 1103-1117.
205. J.L. Martin, M.M. Dieti, P.R. Hof, J.M. Palacios, P.J. Magistretti, Autoradiographic mapping of [mono[125I]iodo-Tyr10, MetO17] vasoactive intestinal peptide binding sites in the rat brain, Neuroscience 23 (1987) 539-565.
206. H. Maruyama, S. Makino, T. Noguchi, T. Nishioka, K. Hashimoto, Central type 2 corticotropin-releasing hormone receptor mediates hypothalamic-pituitary-adrenocortical axis activation in the rat, Neuroendocrinology 86 (2007) 1-16.
207. M. Maruyama, H. Matsumoto, K. Fujiwara, J. Noguchi, C. Kitada, S. Hinuma, H. Onda, O. Nishimura, M. Fujino, T. Higuchi, K. Inoue, Central administration of prolactin-releasing peptide stimulates oxytocin release in rats, Neurosci. Lett. 276 (1999) 193-196.
208. Y. Masuo, T. Ohtaki, Y. Masuda, M. Tsuda, M. Fujino, Binding sites for pituitary adenylate cyclase activating polypeptide (PACAP): comparison with vasoactive intestinal polypeptide (VIP) binding site localization in rat brain sections, Brain Res. 575 (1992) 113-123.
209. L.A. Matsuda, T.I. Bonner, S.J. Lolait, Localization of cannabinoid receptor mRNA in rat brain, J. Comp. Neurol. 327 (1993) 535-550.
210. M. Matsumoto, S. Beltaifa, C.S. Weickert, M.M. Herman, T.M. Hyde, R.C. Saunders, B.K. Lipska, D.R. Weinberger, J.E. Kleinman, A conserved mRNA expression of SREB2 (GPR85) in adult human, monkey, and rat forebrain, Brain Res. Mol. Brain Res. 138 (2005) 58-69.
211. M. Matsumoto, M. Maruyama, J. Noguchi, Y. Horikoshi, K. Fujiwara, C. Kitada, S. Hinuma, H. Onda, O. Nishimura, K. Inoue, M. Fujino, Stimulation of corticotropin-releasing hormone-mediated adrenocorticotropin secretion by central administration of prolactin-releasing peptide in rats, Neurosci. Lett. 285 (2000) 234-238.
212. Y. Matsuoka, T. Furuyashiki, H. Bito, F. Ushikubi, Y. Tanaka, T. Kobayashi, S. Muro, N. Satoh, T. Kayahara, M. Higashi, A. Mizoguchi, H. Shichi, Y. Fukuda, K. Nakao, S. Narumiya, Impaired adrenocorticotropic hormone response to bacterial endotoxin in mice deficient in prostaglandin E receptor EP1 and EP3 subtypes, Proc. Natl. Acad. Sci. USA 100 (2003) 4132-4137.
213. M.J. McKinley, P. Burns, L.M. Covill, B.J. Oldfield, J.D. Wade, R.S. Weisinger, G.W. Tregear, Distribution of fos immunoreactivity in the lamina terminalis and hypothalamus induced by centrally administered relaxin in conscious rats, J. Neuroendocrinol. 9 (1997) 431-437.
214. S.L. Meddle, V.R. Bishop, E. Gkoumassi, F.W. van Leeuwen, A.J Douglas, Dynamic changes in oxytocin receptor expression and activation at parturition in the rat brain, Endocrinology 148 (2007) 5095-5104.
215. S.L. Meddle, P.M. Bull, G.L. Leng, J.A. Russell, M. Ludwig, Somatostatin actions on rat supraoptic nucleus oxytocin and vasopressin neurons, J. Neuroendocrinol. 22 (2010) 438-445.
216. A.D. Medhurst, C.A. Jennings, M.J. Robbins, R.P. Davis, C. Ellis, K.Y. Winborn, K.W. Lawrie, G. Hervieu, G. Riley, J.E. Bolaky, N.C. Herrity, P. Murdock, J.G. Darker, Pharmacological and immunohistochemical characterization of the APJ receptor and its endogenous ligand apelin, J. Neurochem. 84 (2003) 1162-1172.
217. B. Meister, C. Broberger, M.J. Villar, T. Hökfelt, Cholecystokinin B receptor gene expression in hypothalamic neurosecretory neurons after experimental manipulations, Neuroendocrinology 60 (1994) 458-469.
218. F. Mennicken, C. Hoffert, M. Pelletier, S. Ahmad, D. O’Donnell, Distribution of galanin receptor 3 (GalR3) mRNA in the adult rat central nervous system, J. Chem. Neuroanat. 24 (2002) 257-268.
219. I. Merchenthaler, M. Lane, P. Shughrue, Distribution of Pre-Pro-Glucagon and Glucagon-Like Peptide-1 Receptor Messenger RNAs in the Rat Central Nervous System, J. Comp. Neurol. 403 (1999) 261-280.
220. T. Mitsuma, N. Rhue, M. Kayama, Y. Mori, K. Adachi, Y. Yokoi, J. Ping, T. Nogimori, Y. Hirooka, Distribution of calcium sensing receptor in rats: an immunohistochemical study, Endocr. Regul. 33 (1999) 55-59.
221. S.M. Molineaux, T.M. Jessell, R. Axel, D. Julius, 5-HT1c receptor is a prominent serotonin receptor subtype in the central nervous system, Proc. Natl. Acad. Sci. USA 86 (1989) 6793-6797.
222. K. Nakamura, T. Kaneko, Y. Yamashita, H. Hasegawa, H. Katoh, M. Negishi, Immunohistochemical localization of prostaglandin EP3 receptor in the rat nervous system, J. Comp. Neurol. 421 (2000) 543-569.
223. C.R. Neal, A. Mansour, R. Reinscheid, H-P. Nothacker, O. Civelli, H. Akil, S.J. Watson, Opioid receptor-like (ORL) receptor distribution in the rat central nervous system: comparison of OLR1 receptor mRNA expression with 125I[14Tyr]-orphanin FQ binding, J. Comp. Neurol. 412 (1999) 563-605.
224. L. Negri, R. Lattanzi, E. Giannini, M. De Felice, A. Colucci, P. Melchiorri, Bv8, the amphibian homologue of the mammalian prokineticins, modulates ingestive behaviour in rats, Br. J. Pharmacol. 142 (2004) 181-191.
225. M.J. Newson, E.M. Roberts, G.R. Pope, S.J. Lolait, A-M. O’Carroll, The effects of apelin on hypothalamic-pituitary-adrenal axis neuroendocrine function are mediated through corticotrophin-releasing factor- and vasopressin-dependent mechanisms, J. Endocrinol. 202 (2009) 123-129.
226. A. Nicot, A. Berod, D. Gully, W. Rowe, R. Quirion, E.R. de Kloet, W. Rostene, Blockade of neurotensin binding in the rat hypothalamus and of the central action of neurotensin on the hypothalamic-pituitary-adrenal axis with non-peptide receptor antagonists, Neuroendocrinology 59 (1994) 572-578.
227. K.N. Nilaweera, D. Wilson, L. Bell, J.G. Mercer, P.J. Morgan, P. Barrett, G protein-coupled receptor 101 mRNA expression in supraoptic and paraventricular nuclei in rat hypothalamus is altered by pregnancy and lactation, Brain Res. 1193 (2008) 76-83.
228. C.M. Niswender, P.J. Conn, Metabotropic glutamate receptors: physiology, pharmacology, and disease, Annu. Rev. Pharmacol. Toxicol. 50 (2010) 295-322.
229. M. Nomura, Y. Ueta, R. Serino, N. Kabashima, I. Shibuya, H. Yamashita, Pacap type I receptor gene expression in the paraventricular and supraoptic nuclei of rats, NeuroReport 8 (1996) 67-70.
230. M. Nomura, Y. Ueta, R. Serino, Y. Yamamoto, I. Shibuya, H. Yamashita, Effects of centrally administered pituitary adenylate cyclase-activating polypeptide on c-fos gene expression and heteronuclear RNA for vasopressin in rat paraventricular and supraoptic nuclei, Neuroendocrinology 69 (1999) 167-180.
231. A-M O'Carroll, A.L. Don, S.J. Lolait, APJ receptor mRNA expression in the rat hypothalamic paraventricular nucleus: regulation by stress and glucocorticoids, J. Neuroendocrinol. 15 (2003) 1095-1101.
232. A-M O'Carroll, S.J. Lolait, Regulation of rat APJ receptor messenger ribonucleic acid expression in magnocellular neurones of the paraventricular and supraopric nuclei by osmotic stimuli, J. Neuroendocrinol. 15 (2003) 661-666.
233. A-M. O'Carroll, T.L. Selby, M. Palkovits, S.J. Lolait, Distribution of mRNA encoding B78/apj, the rat homologue of the human APJ receptor, and its endogenous ligand apelin in brain and peripheral tissues, Biochim. Biophys. Acta. 1492 (2000) 72-80.
234. B.F. O’Dowd, D.K. Lee, W. Huang, T. Nguyen, R. Cheng, Y. Liu, B. Wang, M.C. Gershengorn, S.R. George, TRH-R2 exhibits similar binding and acute signalling but distinct regulation and anatomic distribution compared with TRH-R1, Mol. Endo. 14 (2000) 183-193.
235. H. Ohishi, R. Shigemoto, S. Nakanishi, N. Mizuno, Distribution of the mRNA for a metabotropic glutamate receptor (mGluR3) in the rat brain: an *in situ* hybridization study, J. Comp. Neurol. 335 (1993) 252-266.
236. T. Oka, K. Oka, T.E. Scammell, C. Lee, J.F. Kelly, F. Nantel, J.K. Elmquist, C.B. Saper. Relationship of EP1-4 prostaglandin receptors with rat hypothalamic cell groups involved in lipopolysaccharide fever responses. J. Comp. Neurol. 428 (2000) 20-32.
237. S. Okuya, K. Inenaga, T. Kaneko, H. Yamashita, Angiotensin II sensitive neurons in the supraoptic nucleus, subfornical organ and anteroventral third ventricle of rats in vitro, Brain Res. 402 (1987) 58-67.
238. S.H. Oliet, D.A. Poulain, Adenosine-induced presynaptic inhibition of IPSCs and EPSCs in rat hypothalamic nucleus neurons, J. Physiol. 520 (1999) 815-825.
239. P.K. Olszewski, E.M. Bomberg, A. Martell, M.K. Grace, A.S. Levine, Intraventricular ghrelin activates oxytocin neurons: implications in feeding behavior, Neuroreport. 18 (2007) 499-503.
240. P.K. Olszewski, M.M. Wirth, T.J. Shaw, M.K. Grace, A.S. Levine, Peptides that regulate food intake: effect of peptide histidine isoleucine on consummatory behavior in rats, Am. J. Physiol. Regul. Integr. Comp. Physiol. 284 (2003) 1445-1453.
241. S.I. Ortiz-Miranda, G. Dayanithi, V. Coccia, E.E. Custer, S. Alphandery, E. Mazuc, S. Treistman, J.R. Lemos, mu-Opioid receptor modulates peptide release from rat neurohypophysial terminals by inhibiting Ca(2+) influx, J. Neuroendocrinol. 15 (2003) 888-894.
242. P. Osei-Owusu, A. James, J. Crane, K.E. Scrogin, 5-Hydroxytryptamine 1A receptors in the paraventricular nucleus of the hypothalamus mediate oxytocin and adrenocorticotropin hormone release and some behavioral components of the serotonin syndrome, J. Pharmacol. Exp. Ther. 313 (2005) 1324-1330.
243. H. Otsubo, S. Hyodo, H. Hashimoto, M. Kawasaki, H. Suzuki, T. Saito, T. Ohbuchi, T. Yokoyama, H. Fujihara, T. Matsumoto, Y. Takei, Y. Ueta, Centrally administered adrenomedullin 5 activates oxytocin-secreting neurons in the hypothalamus and elevates plasma oxytocin level in rats, J. Endocrinol. 202 (2009) 237-247.
244. S.L. Parker, W.R. Crowley, Central stimulation of oxytocin release in the lactating rat: interaction of neuropeptide Y with alpha-1-adrenergic mechanisms, Endocrinology 132 (1993) 658-666.
245. R.M.C. Parker, H. Herzog, Regional distribution of Y-receptor subtype mRNAs in rat brain, Eur. J. Neurosci. 11 (1999) 1431–1448.
246. D. Parkes, S. Rivest, S. Lee, C. Rivier, W. Vale W, Corticotropin-releasing factor activates c-fos, NGFI-B, and corticotropin-releasing factor gene expression within the paraventricular nucleus of the rat hypothalamus, Mol. Endo. 7 (1993) 1357-1367.
247. M. Patterson, K.G. Murphy, E.L. Thompson, S. Patel, M.A. Ghatei, S.R. Bloom, Administration of kisspeptin-54 into discrete regions of the hypothalamus potently increases plasma luteinizing hormone and testosterone in male adult rats, J. Neuroendocrinol. 18 (2006) 349-354.
248. J. Pfister, C. Spengler, E. Grouzmann, M.K. Raizada, D. Felix, H. Imboden, Intracellular staining of angiotensin receptors in the PVN and SON of the rat, Brain Res. 754 (1997) 307–310.
249. C. Pillot, A. Heron, V. Cochois, J. Tardivel-Lacombe, X. Ligneau, J.C. Schwartz, J.M. Arrang, A detailed mapping of the histamine H(3) receptor and its gene transcripts in rat brain, Neuroscience 114 (2002) 173-193.
250. Z. Pirnik, D. Jezova, J.D. Mikkelsen, A. Kiss, Xylazine activates oxytocinergic but not vasopressinergic hypothalamic neurons under normal and hyperosmotic conditions in rats, Neurochem. Int. 47 (2005) 458-465.
251. T.A. Ponzio, Y-F. Wang, G.I. Hatton, Activation of adenosine A2A receptors alters postsynaptic currents and depolarises neurons of the supraoptic nucleus, Am. J. Physiol. Regul. Integr. Comp. Physiol. 291 (2006) 359-366.
252. C.J. Price, Q.J. Pittman, Dopamine D4 receptor activation inhibits presynaptically glutamatergic neurotransmission in the rat supraoptic nucleus, J. Neurophysiol. 86 (2001) 1149-1155.
253. F. Qadri, EC Schwartz, W. Häuser, O. Jöhren, W. Müller-Esterl, P. Dominiak, Kinin B2 receptor localization and expression in the hypothalamo–pituitary–adrenal axis of spontaneously hypertensive rats, Int. Immunopharmacol. 3 (2003) 285–292.
254. F. Qadri, T. Waldmann, A. Wolf, S. Hohle, W. Rascher, T. Unger, Differential contribution of angiotensinergic and cholinergic receptors in the hypothalamic paraventricular nucleus to osmotically induced AVP release, J. Pharmacol. Exp. Ther. 285 (1998) 1012-1018.
255. D.L. Qiu, C.P. Chu, T. Shirasaka, T. Nabekura, T. Kunitake, K. Kato, M. Nakazato, T. Katoh, H. Kannan, Neuromedin U depolarises rat hypothalamic paraventricular nucleus neurons in vitro by enhancing *I*_H_ channel activity, J. Neurophysiol. 90 (2003) 843-850.
256. D.L. Qiu, C.P. Chu, H. Tsukino, T. Shirasaka, H. Nakao, K. Kato, T. Kunitake, T. Katoh, H. Kannan, Neuromedin U receptor subtype -2 mRNA and HCN channels mRNA expression NMU-sensitive neurons in rat hypothalamic paraventricular nucleus, Neurosci. Lett. 374 (2005) 69-72.
257. D.S. Richards, R.M. Villalba, F.J Alvarez, J.E. Stern, Expression of GABAB receptors in magnocellular neurosecretory cells of male, virgin female and lactating rats, J. Neuroendocrinol. 17 (2005) 413-423.
258. M. Rigby, R. O'Donnell, N.M. Rupniak, Species differences in tachykinin receptor distribution: further evidence that the substance P (NK1) receptor predominates in human brain, J. Comp. Neurol.490 (2005) 335-353.
259. S.A. Rivkees, S.L. Price, F.C. Zhou, Immunohistochemical detection of A 1 adenosine receptors in rat brain with emphasis; on localization in the hippocampal formation, cerebral cortex, cerebellum, and basal ganglia, Brain Res. 677 (1995) 193-203.
260. K.V. Rogers, C.K. Dunn, S.C. Hebert, E.M. Brown, Localization of calcium receptor mRNA in the adult rat central nervous system by in situ hybridization, Brain Res. 744 (1997) 47-56.
261. B.L. Roland, S.W. Sutton, S.J. Wilson, L. Luo, J. Pyati, R. Huvar, M.G. Erlander, T.W. Lovenberg, Anatomical distribution of prolactin-releasing peptide and its receptor suggests additional functions in the central nervous system and periphery, Endocrinology 140 (1999) 5736-5745.
262. I.V. Romanova, E.J. Ramos, Y. Xu, R. Quinn, C. Chen, Z.M. George, A. Inui, U. Das, M.M Meguid, Neurobiologic Changes in the Hypothalamus Associated with Weight Loss after Gastric Bypass, J. Am. Coll. Surg. 199 (2004) 887-895.
263. D.L. Rosin, A. Robeva, R.L. Woodward, P.G. Guyenet, J. Linden, Immunohistochemical localization of adenosine A2A receptors in the rat central nervous system, J. Comp. Neurol. 401 (1998) 163-186.
264. N.F. Rossi, Regulation of vasopressin secretion by ETA and ETB receptors in compartmentalized rat hypothalamo-neurohypophysial explants, Am. J. Physiol. Endocrinol. Metab. 286 (2004) 535-541.
265. M. Rossi, S.A. Beak, S.J. Choi, C.J. Small, D.G. Morgan, M.A. Ghatei, D.M. Smith, S.R. Bloom, Investigation of the feeding effects of melanin concentrating hormone on food intake – action independent of galanin and the melanocortin receptors, Brain Res. 846 (1999) 164-170.
266. D.V. Rossi, Y. Dai, P. Thomas, G.A. Carrasco, L.L. DonCarlos, N.A. Muma, Q. Li, Estradiol-induced desensitization of 5-HT1A receptor signaling in the paraventricular nucleus of the hypothalamus is independent of estrogen receptor-beta, Psychoneuroendocrinology 7 (2010) 1023-1033.
267. W. Rowe, V. Viau, M.J. Meaney, R. Quirion, Stimulation of CRF-mediated ACTH secretion by central administration of neurotensin: evidence for the participation of the paraventricular nucleus, J. Neuroendocrinol. 7 (1995) 109-117.
268. S.H. Russell, C.J. Small, C.L. Dakin, C.R. Abbott, D.G. Morgan, M.A. Ghatei, S.R. Bloom, The central effects of orexin-A in the hypothalamic-pituitary-adrenal axis in vivo and in vitro in male rats, J. Neuroendocrinol. 13 (2001) 561-566.
269. N. Sabatier, C.H. Brown, M. Ludwig, G. Leng, Phasic spike patterning in rat supraoptic neurones in vivo and in vitro, J. Physiol. 558 (2004) 161-180.
270. N. Sabatier, C. Caquineau, G. Dayanithi, P. Bull, A.J. Douglas, X.M. Guan, M. Jiang, L. Van der Ploeg, G. Leng, α-Melanocyte-stimulating hormone stimulates oxytocin release from the dendrites of hypothalamic neurons while inhibiting oxytocin release from their terminals in the neurohypophysis, J. Neurosci. 23 (2003) 10351-10358.
271. N. Sabatier, G. Leng, Presynaptic action of endocannabinoids mediate α-MSH-induced inhibition of oxytocin cells, Am. J. Physio. Integr. Comp. Physiol. 290 (2006) 577-584.
272. R. Sah, L.M. Pritchard, N.M. Richtand, R. Ahlbrand, K. Eaton, F.R. Sallee, J.P. Herman, Expression of the glucocorticoid-induced receptor mRNA in rat brain, Neuroscience 133 (2005) 281-292.
273. T. Sakamoto, K. Mori, M. Miyazato, K. Kangawa, H. Sameshima, K. Nakahara, N. Murakami, Involvement of neuromedin S in the oxytocin release response to suckling stimulus, Biochem. Biophys. Res. Comm. 375 (2008) 49-53.
274. W.K. Samson, J.R. Baker, C.K. Samson, H. Samson, M.M Taylor, Central neuropeptide B administration activates stress hormone secretion and stimulates feeding in male rats, J. Neuroendocrinol. 16 (2004) 842-849.
275. S.A. Sands, D.A. Morilak, Expression of alpha1D adrenergic receptor messenger RNA in oxytocin- and corticotropin-releasing hormone-synthesizing neurons in the rat paraventricular nucleus, Neuroscience 91 (1999) 639–649.
276. K. Sango, H. Yanagisawa, E. Kawakami, S. Takaku, K. Ajiki, K. Watabe, Spontaneously immortalized Schwann cells from adult Fischer rat as a valuable tool for exploring neuron-Schwann cell interactions, J. Neurosci. Res (2011) In Press.
277. L.A. Schrader, J.G. Tasker, Presynaptic modulation by metabotropic glutamate receptors of excitatory and inhibitory synaptic inputs to hypothalamic magnocellular neurons, J. Neurophysiol. 77 (1997) 527-536.
278. V.S. Setiadji, I. Shibuya, N. Kabashima, N. Ibrahim, N. Harayama, Y. Ueta, H. Yamashita, Actions of prostaglandin E2 on rat supraoptic neurons, J. Neuroendocrinol. 10 (1998) 927-936.
279. S.G. Shelat, L.P. Reagan, J.L. King, S.J. Fluharty, L.M. Flanagan-Cato, Analysis of angiotensin type 2 receptors in vasopressinergic neurons and pituitary in the rat, Regul. Pept. 73 (1998) 103-112.
280. W.J. Sheward, E.M. Lutz, A.J. Harmar, The expression of the calcitonin receptor gene in the brain and pituitary gland of the rat, Neurosci. Lett. 181 (1994) 31-34.
281. J. Shi, M. Landry, G.A. Carrasco, G. Battaglia, N.A. Muma, Sustained treatment with a 5-HT2A receptor agonist causes functional desensitisation and reductions in agonist-labeled 5-HT2A receptors despite increases in receptor protein levels in rats, Neuropharmacology 55 (2008) 687-692.
282. I. Shibuya, S.V. Setiadji, N. Ibrahim, N. Harayama, T. Maruyama, Y. Ueta, H. Yamashita, Involvement of postsynaptic EP4 and presynaptic EP3 receptors in actions of prostaglandin E2 in rat supraoptic nucleus, J. Neuroendocrinol. 14 (2002) 64-72.
283. N. Shibuya, N. Kabashima, K. Tanaka, S. Setiadji, J. Noguchi, N. Harayama, Y. Ueta, H. Yamashita, Patch-clamp analysis of the mechanism of PACAP-induced excitation in rat supraoptic neurons, J. Neuroendocrinol. 10 (1998) 759-768.
284. R. Shigemoto, S. Nakanishi, N. Mizuno, Distribution of the mRNA for a metabotropic glutamate receptor (mGluR1) in the central nervous system: an in situ hybridization study in adult and developing rat, J. Comp. Neurol. 322 (1992) 121-135.
285. Y.B. Shrestha, K. Wickwire, S. Giraudo, Action of MT-II on ghrelin-induced feeding in the paraventricular nucleus of the hypothalamus, NeuroReport 15 (2004) 1365-1367.
286. Y.B. Shrestha, K. Wickwire, S. Giraudo, Effect of reducing ghrelin receptor gene expression on energy balance, Peptides 30 (2009) 1336-1341.
287. B.N. Smith, W.E. Armstrong, The ionic dependence of the histamine-induced depolarization of vasopressin neurones in the rat supraoptic nucleus, J. Physiol. 495 (1996) 465-478.
288. K.L. Smith, M. Patterson, W.S. Dhillo, S.R. Patel, N.M Semjonous, J.V. Gardiner, M.A. Ghatei, S.R. Bloom, Neuropeptide S stimulates the hypothalamo-pituitary-adrenal axis and inhibits food intake, Endocrinology 147 (2006) 3510-3518.
289. Z. Song, D.A. Gomes, W. Stevens, Role of purinergic P2Y1 receptors in regulation of vasopressin and oxytocin secretion, Am. J. Physiol. Regul. Integr. Comp. Physiol. 297 (2009) 478-484.
290. Z. Song, D.A. Gomes, W. Stevens, C.D. Sladek, Multiple α1 adrenergic receptor subtypes support synergistic stimulation of vasopressin and oxytocin release by ATP and phenylephrine, Am. J. Physiol. Regul. Integr. Comp. Physiol. 299 (2010) 1529-1537.
291. Z. Song, S. Vijayaraghavan, C.D. Sladek, Simultaneous exposure to ATP and phenylephrine induces a sustained elevation in the intracellular calcium concentration in supraoptic neurons, Am. J. Physiol. Regul. Integr. Comp. Physiol. 291 (2006) 37-45.
292. Z. Song, S. Vijayaraghavan, C.D. Sladek, ATP increases intracellular calcium in supraoptic neurons by activation of both P2X and P2Y purinergic receptors, Am. J. Physiol. Regul. Integr. Comp. Physiol. 292 (2007) 423-431.
293. S. Succu, F. Sanna, T. Melis, A. Boi, A. Argiolas, M.R. Melis, Stimulation of dopamine receptors in the paraventricular nucleus of the hypothalamus of male rats induces penile erection and increases extra-cellular dopamine in the nucleus accumbens: involvement of central oxytocin, Neuropharmacology 52 (2007) 1034-1043.
294. T. Suda, F. Tozawa, I. Iwai, Y. Sato, T. Sumitomo, Y. Nakano, M. Yamada, H. Demura, Neuropeptide Y increases the corticotropin-releasing factor messenger ribonucleic acid level in the rat hypothalamus, Brain Res. Mol. Brain Res. 18 (1993) 311-315.
295. Y. Sugimura, T. Murase, S. Ishizaki, K. Tachikawa, H. Arima, Y. Miura, T.B. Usdin, Y. Oiso, Centrally administered tuberoinfundibular peptide 39 residues inhibits arginine vasopressin release in conscious rats, Endocrinology 144 (2003) 2791-2796.
296. S.W. Sutton, P. Bonaventure, C. Kuei, B. Roland, J. Chen, D. Nepomuceno, T.W. Lovenberg, C. Liu, Distribution of G-protein-coupled receptor (GPCR) 135 binding sites and receptor mRNA in the rat brain suggests a role for relaxin-3 in neuroendocrine and sensory processing, Neuroendocrinology 80 (2004) 296-307.
297. A. Takahashi, H. Ishimaru, Y. Ikarashi, E. Kishi, Y. Maruyama, Opposite regulation of body temperature by cholinergic input to the paraventricular nucleus and supraoptic nucleus in rats, Brain Res. 909 (2001) 102-111.
298. Y. Takano, Y. Nakayama, T. Matsumoto, R. Saito, H.O. Kamiya, The mechanism of central pressor actions of tachykinin NK-3 receptor in the paraventricular nucleus of the hypothalamus in rats, Regul. Peptides. 46 (1993) 360-363.
299. M. Tanaka, Relaxin-3/insulin-like peptide 7, a neuropeptide involved in the stress response and food intake, FEBS J. 277 (2010) 4990-4997
300. J.B. Tatro, M.L. Entwistle, Heterogeneity of brain mealnocortin receptors suggested by diferential ligand binding in situ, Brain Res. 635 (1994) 148-158.
301. M.M. Taylor, E.A. Yuill, J.R. Baker, C.C. Ferri, A.V. Ferguson, W.K. Samson, Actions of neuropeptide W in paraventricular hypothalamus: implications for the control of stress hormone secretion, Am. J. Physiol. Regul. Integr. Comp. Physiol. 288 (2005) 270-275.
302. V.A. Tobin, P.M. Bull, S. Arunachalam, A-M. O’Carroll, Y. Ueta, M. Ludwig, The Effects of Apelin on the Electrical Activity of Hypothalamic Magnocellular Vasopressin and Oxytocin Neurons and Somatodendritic Peptide Release, Endocrinology 149 (2008) 6136–6145.
303. P. Trivedi, H. Yu, D.J. MacNeil, L.H. Van der Ploeg, X.M. Guan, Distribution of orexin receptor mRNA in the rat brain, FEBS Lett. 438 (1998) 71-75.
304. S. Tsagarakis, L.H. Rees, G.M. Besser, A. Grossman, Neuropeptide-Y stimulates CRF-41 release from rat hypothalami in vitro, Brain Res. 502 (1989) 167-170.
305. K. Tsuda, S. Tsuda, I. Nishio, Y. Masuyama, M. Goldstein, Synergistic effects of Bay K8644 and bradykinin on norepinephrine release in the hypothalamus of spontaneously hypertensive rats, Clin. Exp. Pharmacol. Physiol. Suppl. 22 (1995) 54-57.
306. Y. Ueta, R. Serino, I. Shibuya, K. Kitamura, K. Kangawa, J.A. Russell, H. Yamashita, A physiological role for adenomedullin in rats: a potent hypotensive peptide in the hypothalamo-neurohypophysial system, Exp. Physiol. 85 (2000) 163-169.
307. J.H. Urban, R.J. Leitermann, M.R. DeJoseph, S.J. Somponpun, M.L. Wolak, C.D. Sladek, Influence of Dehydration on the Expression of Neuropeptide Y Y1 Receptors in Hypothalamic Magnocellular Neurons, Endocrinology 147 (2006) 4122–4131.
308. B.J. Van de Heijning, I. Koehhoek-Van den Herik, T.B. Van Wimersma Greidanus, The opioid receptor subtypes mu and kappa, but not delta, are involved in the control of the vasopressin and oxytocin release in the rat, Eur. J. Pharmacol. 17 (1991) 199-206.
309. E.H. van den Burg, I.D. Neumann, Bridging the Gap between GPCR activation and behaviour: oxytocin and prolactin signalling in the hypothalamus, J. Mol. Neurosci. 43 (2011) 200-208.
310. K. Van Pett, V. Viau, J.C. Bittencourt, R.K. Chan, H.Y. Li, C. Arias, G.S. Prins, M. Perrin, W. Vale, P.E. Sawchenko, Distribution of mRNAs encoding CRF receptors in brain and pituitary of rat and mouse, J. Comp. Neurol. 428 (2000) 191-212.
311. P. Vertongen, S.N. Schiffmann, P. Gourlet, P. Robberecht, Autoradiographic visualization of the receptor subclasses for vasoactive intestinal polypeptide (VIP) in rat brain, Peptides 18 (1997) 1547-1554.
312. C. Vuong, S.H. Van Uum, L.E. O’Dell, K. Lutfy, T.C. Friedman, The effects of opioids and opioid analogs on animal and human endocrine systems, Endocr. Rev. 31 (2010) 98-132.
313. E. Wada, J. Way, H. Shapira, K. Kusano, A.M. Lebacq-Verheyden, D. Coy, R. Jensen, J. Battery, cDNA cloning, characterization, and brain region-specific expression of a neuromedin-B-preferring bombesin receptor, Neuron 6 (1991) 421-430.
314. T. Wang, M. Palkovits, M. Rusnak, E. Mezey, T.B. Usdin, Distribution of parathyroid hormone 2 receptor-like immunoreactivity and messenger mRNA in the rat nervous system, Neuroscience 100 (2000) 629-649.
315. Y.F. Wang, T.A. Ponzio, G.I. Hatton, Autofeedback effects of progressively rising oxytocin concentrations on supraoptic oxytocin neuronal activity in slices from lactating rats, Am. J. Physiol. Regul. Integr. Comp. Physiol. 290 (2006) 1191-1198.
316. H.L. Ward, C.J. Small, K.G. Murphy, A.R. Kennedy, M.A. Ghatie, S.R. Bloom, The actions of tuberoinfundibular peptide on the hypothalamo-pituitary axes, Endocrinology 142 (2001) 3451-3456.
317. A.M. Watson, M.J. McKinley, C.N. May, Effect of central urotensin II on the heart rate, blood pressure and brain fos immunoreactivity in conscious rats, Neuroscience 155 (2008) 241-249.
318. D.R.Weaver, J.D Deeds, K. Lee, G.V.Segre, Localization of parathyroid hormone-related peptide (PTHrP) and PTH/PTHrP receptor mRNAs in rat brain, Mol. Brain. Res. 28 (1995) 296-310.
319. S.G. Wei, Y. Yu, Z.H. Zhang, R.B. Felder, Angiotensin II upregulates hypothalamic AT1 receptor expression in rats via the mitogen-activated protein kinase pathway, Am. J. Physiol. Heart Circ. Physiol. 296 (2009) 1425-1433.
320. D.M. Weiner, A.I. Levey, R.K. Sunahara, H.B. Niznik, B.F. O’Dowd, P. Seeman, M.R. Brann, D1 and D2 dopamine receptor mRNA in rat brain, Proc. Natl. Acad. Sci. USA 88 (1991) 1859-1863.
321. A.M. Williams, D.A. Morilak, alpha1B adrenoceptors in rat paraventricular nucleus overlap with, but do not mediate, the induction of c-Fos expression by osmotic or restraint stress, Neuroscience 76 (1997) 901-913.
322. J.O. Willoughby, W.W. Blessing, Neuropeptide Y injected into the supraoptic nucleus causes secretion of vasopressin in the unanesthetized rat, Neurosci. Lett. 75 (1987) 17-22.
323. M.L. Wolak, M.R. deJoseph, A.D. Cator, A.S. Mokashi, M.S. Brownfield, J.H. Urban, Comparative distribution of neuropeptide Y Y1 and Y5 receptors in the rat brain by using immunohistochemistry, J. Comp. Neurol. 464 (2003) 285-311.
324. C.T. Wotjak, M. Kubota, G. Liebsch, A. Montkowski, F. Holsboer, I. Neumann, R. Landgraf, Release of vasopressin within the rat paraventricular nucleus in response to emotional stress: a novel mechanism or regulating adrenocorticotropin hormone secretion, J. Neurosci. 16 (1996) 7725-7732.
325. C.T. Wotjak, M. Ludwig, R. Langraf, Vasopressin facilitates its own release within the rat supraoptic nucleus in vivo, Neuroreport 5 (1994) 1181-1184.
326. A.M. Wren, C.J. Small, C.R. Abbott, P.H. Jethwa, A.R. Kennedy, K.G. Murphy, S.A. Stanley, A.N. Zollner, M.A. Ghatei and S.R. Bloom, Hypothalamic Actions of Neuromedin U, Endocrinology 143 (2002) 4227-4234.
327. D.E. Wright, K.B. Seroogy, K.H. Lundgren, B.M. Davis, L. Jennes, Comparative localization of serotonin 1A, 1C, and 2 receptor subtype mRNAs in rat brain, J. Comp. Neurol. 351 (2004) 357-373.
328. M. Wu, M. Tang, D. Adriaensen, I. Depoortere, T.L. Peeters, J.P. Timmermans, Central, but not peripheral application of motilin increases c-Fos expression in hypothalamic nuclei in the rat brain, Histochem. Cell Biol. 123 (2005) 139-145.
329. Y.L. Xu, C.M. Gall, V.R. Jackson, O. Civelli, R.K Reinscheid, Distribution of Neuropeptide S Receptor mRNA and Neurochemical Characteristics of Neuropeptide S Expressing Neurons in the Rat Brain, J. Comp. Neurol. 500 (2007) 84-102.
330. H. Xu, S. Qin, G.A. Carrasco, Y. Dai, E.J. Filardo, E.R. Prossnitz, G. Battaglia, L.L. Doncarlos, N.A. Muma, Extra-nuclear estrogen receptor GPR30 regulates serotonin function in rat hypothalamus, Neuroscience 158 (2009) 1599-1607.
331. T. Yamada, A. Mochiduki, Y. Sugimoto, Y. Suzuki, K. Itoi, K. Inoue, Prolactin-releasing peptide regulates the cardiovascular system via corticotrophin-releasing hormone, J. Neuroendocrinol. 21 (2009) 586-593.
332. H. Yamashita, K. Inenaga, H. Kannan, Depolarizing effect of noradrenaline on neurons of the rat supraoptic nucleus in vitro, Brain Res. 405 (1987) 348-352.
333. S.A. Yasin, A. Costa, G.M. Besser, D. Hucks, A. Grossman, M.L. Forsling, Melatonin and its analogs inhibit the basal and stimulated release of hypothalamic vasopressin and oxytocin in vitro, Endocrinology 132 (1993) 1329-1336.
334. H. Yokoi, H. Arima, K. Kondo, T. Murase, Y. Iwasaki, H.Y. Yang, Y. Oiso, Antiserum against neuropeptide FF augments vasopressin release in conscious rats, Peptides 19 (1998) 393-395.
335. T. Yokoyama, T. Saito, T. Ohbuchi, H. Suzuki, H. Otsubo, T. Okamoto,H. Fujihara, T. Nagatomo, Y. Ueta, Ghrelin potentiates miniature excitatory postsynaptic currents in supraoptic magnocellular neurons, J. Neuroendocrinol. 21 (2009) 910-920.
336. E.A. Yuill, T.D. Hoyda, C.C. Ferri, Q.Y. Zhou, A.V. Ferguson, Prokineticin 2 depolarizes paraventricular nucleus magnocellular and parvocellular neurons, Eur. J. Neurosci. 25 (2007) 425-434.
337. Y. Zhang, KJ Damjanoska, G.A. Carrasco, B. Dudas, D.N. D'Souza, J. Tetzlaff, F. Garcia, N.R. Hanley, K. Scripathirathan, B.R. Petersen, T.S. Gray, G. Battaglia, N.A. Muma, L.D. Van de Kar, Evidence that 5-HT2A receptors in the hypothalamic paraventricular nucleus mediate neuroendocrine responses to (-)DOI, J. Neurosci. 22 (2002) 9635-9642.
338. S.J. Zhang, Y.M. Deng, Y.L. Zhu, X.W. Dong, J.X. Jiang, Q.M. Xie, Intracerebroventricular injection of leukotriene B4 attenuates antigen-induced asthmatic response via BLT1 receptor stimulating HPA-axis in sensitized rats, Respir. Res. 11 (2010) 39-47.
339. Y. Zhang, T.S. Gray, D.N. D'Souza, G.A. Carrasco, K.J. Damjanosk, B. Dudas, F. Garcia, G.M. Zainelli, N.R. Sullivan Hanley, G. Battaglia, N.A. Muma, L.D.Van de Kar, Desensitization of 5-HT1A receptors by 5-HT2A receptors in neuroendocrine neurons in vivo, J. Pharmacol. Exp. Ther. 310 (2004) 59-66.
340. J Zhang, S. Rivest, Distribution, regulation and colocalization of the genes encoding the EP2- and EP4-PGE2 receptors in the rat brain and neuronal responses to systemic inflammation, Eur. J. Neurosci. 11 (1999) 2651-2668.
341. J. Zhang, S. Rivest, A functional analysis of EP4 receptor expressing neurons in mediating the action of prostaglandin E2 within specific nuclei of the brain in response to circulating interleukin-1β, J. Neurochem. 74 (2000) 2134-2145.
342. X.J. Zhou, J. Yang, F.L. Yan, D.X. Wang, X.Y. Li, X.Q. Fan, F. Hao, X.Q. Yan, X.P. Li, H. Li, W.Y. Liu, B.C. Lin, Norepinephrine plays an important role in antinociceptive modulation of hypothalamic paraventricular nucleus in the rat, Int. J. Neurosci. 120 (2010) 428-438.
343. G.Q. Zhu, L. Gao, Y. Li, K.P. Patel, I. H. Zucker, W. Wang, AT1 receptor mRNA antisense normalizes enhanced cardiac sympathetic afferent reflex in rats with chronic heart failure, Am. J. Physiol. Heart Circ. Physiol. 287 (2004) 1828-1835.
344. G.Q. Zhu, K.P. Patel, I.H. Zucker, W. Wang, Microinjection of ANG II into paraventricular nucleus enhances cardiac sympathetic afferent reflex in rats, Am. J. Physiol. Heart Circ. Physiol. 282 (2002) 2039-2045.
345. J.M. Zigman, J.E. Jones, C.E. Lee, C.B. Saper, J.K. Elmquist, Expression of the ghrelin receptor mRNA in the rat and the mouse brain, J. Comp. Neurol. 494 (2006) 528-548.
